# Designer Dynamic DNA Nanoaggregate in Living Cell for Mitochondrial Energy Restriction

**DOI:** 10.1002/advs.76061

**Published:** 2026-06-09

**Authors:** Ruijia Deng, Jing Sheng, Ben Niu, Wenjuan Fu, Yingjie Yang, Zuowei Xie, Shuang Xie, Yunxuan He, Meilin Gong, Jue Wang, Yan Pi, Ming Chen, Kai Chang

**Affiliations:** ^1^ Department of Clinical Laboratory Medicine Southwest Hospital Third Military Medical University (Army Medical University) Chongqing China; ^2^ Department of Rehabilitation Medicine The First Affiliated Hospital of Chongqing Medical University Chongqing China; ^3^ State Key Laboratory of Trauma and Chemical Poisoning Army Medical University Chongqing China

**Keywords:** artificial nanoaggregates, DNA tetrahedron, mitochondrial energy restriction, telomerase‐responsive assembly

## Abstract

Artificial intracellular aggregations of exogenous molecules are powerful tools for modulating cellular processes and fate; however, controlling spatiotemporal assembly in a subcellular‐specific manner remains challenging. In this study, a designer dynamic DNA nanoaggregate system called Tech‐tetrahedron (telomerase‐controllable hand‐in‐hand tetrahedron) based on a trinity‐functionalized DNA tetrahedron with tailored vertexes was constructed to achieve spatiotemporal mitochondrial energy restriction. The navigation function of the tech‐tetrahedron enables precise mitochondrial anchoring via the triphenylphosphine modification of a single vertex. The enzymatic control function deploys a telomerase‐gated latch that opens upon primer elongation to temporarily release a self‐assembly initiator. The self‐assembly function uses this initiator to trigger catalytic hairpin self‐assembly at two hairpin‐decorated vertices, generating spatial “hand‐in‐hand” DNA nanoaggregates. These DNA nanoaggregates serve as polyanionic barriers that disrupt physiological traits and mitochondrial‐cytoplasmic metabolite exchange, thereby impairing aerobic respiration and glycolysis, and reducing ATP production. Transcriptomic analysis revealed that the increased expression of polycystin‐1 forms a feedback loop with Tech‐tetrahedron, highlighting potential interference with nicotinamide nucleotide transhydrogenase activity. The resulting bioenergetic collapse led to a 24.67% increase in tumor apoptosis and a 63.16% tumor growth inhibition. Overall, Tech‐tetrahedron offers a precise strategy for subcellular energy intervention and a transformative approach to targeted cellular regulation via artificial nanoaggregates.

## Graphical abstract

This study presents the Tech‐tetrahedron, a designer dynamic DNA nanoaggregate engineered for precise mitochondrial energy restriction. Its trinity‐functionalized design integrates navigable unit, telomerase‐activated latch, and self‐assembly module. Upon activation in tumor cells, it forms “hand‐in‐hand” nanoaggregates on mitochondria, creating a barrier that disrupts energy metabolism. This induced bioenergetic collapse significantly inhibits tumor growth, presenting a novel targeted therapy.

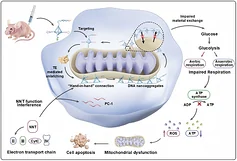

## 1 Introduction

Aggregates within living cells assemble in an orderly manner to execute diverse physiological functions, whereas aberrant aggregation frequently precipitates cellular dysfunction [1, 2, 3]. To capitalize on the natural evolution of functional regulation, artificial aggregate systems have been engineered to modulate biological processes and cellular functions by clustering exogenous molecules [4, 5]. Carbon nanozyme aggregates have been designed to recapitulate peroxidase‐like activity and provide multiple enzyme‐like functions [6, 7]. Furthermore, amino acid‐based nanovehicles have been constructed to enable real‐time monitoring of intracellular structural changes [8, 9]. These advances have substantiated the development of artificial aggregate systems as a potent and viable strategy for cellular function regulation. However, the broader translation of these systems is hindered by the poor cellular adaptability and limited modifiability of the synthetic materials [10, 11]. Consequently, biocompatible and programmable synthetic materials that remain functional in the intracellular environment must be developed.

DNA is known for its ability to achieve dynamic and controlled intracellular aggregation because of its inherent biocompatibility and precise structural programmability [12]. As natural macromolecules, DNA strands can be rationally designed from four nucleotide monomers to form programmable architectures with responsive functionalities [13, 14]. Various DNA precursors, including linear strands, branched structures, and hybrid nanocomplexes, have been engineered using sequence‐specific designs to enable stimulus‐responsive assembly [15, 16, 17]. These systems leverage programmable Watson–Crick pairing or structural motifs to initiate “monomer‐aggregate” transformations under specific triggers like pH and K^+^ changes [18, 19, 20]. Nevertheless, the insufficient rigidity of these DNA precursors can compromise structural integrity, making the resulting aggregate assemblies prone to degradation under varying cellular conditions [21, 22, 23]. Furthermore, the limited spatial structural modifiability often results in a single trigger form and poor addressing capabilities [24, 25, 26]. Therefore, a DNA precursor with enhanced rigidity and diverse structural modifications is needed.

In stark contrast, the DNA tetrahedron stands out because of its unique tetrahedral geometry, which confers unparalleled intrinsic stability and mechanical resilience [27, 28, 29]. This tetrahedral structure renders it significantly more resistant to environmental degradation and structural collapse than other DNA precursors [30, 31]. As a programmable assembly unit, a DNA tetrahedron exploits spatially precise and independently addressable vertices to enable versatile structural modifications with high topological control [32, 33]. These modifications can be achieved through sequence‐dependent strategies, such as base‐pair extension and sequence‐specific hybridization [34], as well as non‐covalent interactions, including the intercalation of functional moieties or encapsulation of payloads [35, 36], collectively supporting dynamic topological adaptations [37]. Such precise engineering enables multimodal trigger responses while offering addressable anchoring points for the assembly of artificial aggregates. Therefore, DNA tetrahedra (the plural form of “tetrahedron”) can be prime and superior structural precursors for constructing versatile artificial aggregate systems.

In this study, we designed a dynamic DNA nanoaggregate system named Tech‐tetrahedron based on a trinity‐functionalized DNA tetrahedron precursor for mitochondria‐targeted energy restriction. This system establishes a paradigm of structure‐driven organelle interference, distinguishing it from traditional DNA nanodevices that serve as passive carriers for exogenous drugs. Tech‐tetrahedron is a payload‐free platform that achieves therapeutic efficacy through a telomerase‐triggered, in situ structural transformation. Specifically, it utilizes a dual‐state regulation logic: in the “latched” state, catalytic hairpin assembly (CHA) initiators are physically sequestered; upon sensing high telomerase expression, a primer‐elongation‐induced conformational shift “unlocks” the system. This releases initiators to drive the hand‐in‐hand assembly of dense DNA nanoaggregates directly on mitochondrial membranes. In this paradigm, the assembly event itself is the functional effector: the resulting polyanionic barrier physically disrupts mitochondrial‐cytoplasmic metabolite exchange, inducing a severe bioenergetic crisis through the dual inhibition of aerobic respiration and glycolysis. Transcriptomic analysis further revealed that this structure‐mediated intervention triggers a self‐reinforcing feedback loop via polycystin‐1 and NNT modulation. By demonstrating that programmed structural reconfiguration can directly regulate organelle function, this work provides a conceptual blueprint for the targeted regulation of cellular processes and fate.

## 2 Results

### 2.1 Operating Principle of the Tech‐Tetrahedron

Tech‐tetrahedron is assembled from a trinity‐functionalized DNA tetrahedron, with each of its four vertices modified with a distinct functional element: (i) triphenylphosphine (TPP) for navigating mitochondrial function, (ii) a telomerase‐gated latch for enzymatic control, and (iii) two hairpins (H1 and H2) that participate in CHA reactions to facilitate self‐assembly. Specifically, the TPP group is conjugated to the P3 strand via a click reaction, while the telomerase primer (TP) is incorporated at the terminus of the P4 strand. The telomerase‐gated latch comprises of two strands: P5, which acts as a bridge by hybridizing with partial sequences of both TP and P6, thereby linking P4 and P6. The freed P6 strand then binds to the toehold domain of H1, initiating its conformational opening.

**FIGURE 1:**
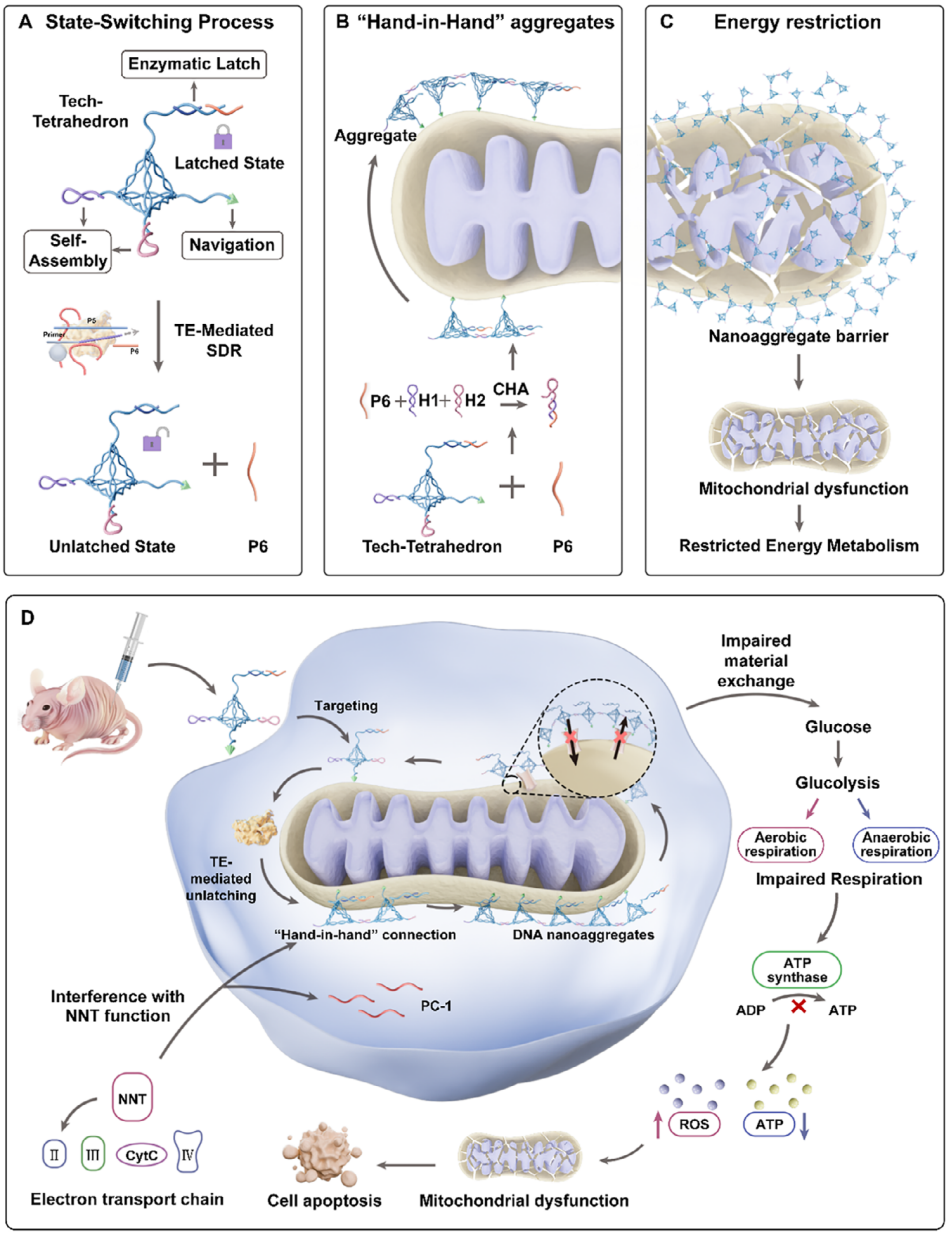
Operating Principle of the Tech‐Tetrahedron. (A) State‐switching process. Tech‐tetrahedron was assembled on trinity‐functionalized tetrahedral DNA: (i) TPP for navigating mitochondrial function, (ii) telomerase‐gated latch for enzymatic control (primer P4 + bridge P5 + blocker P6) and (iii) two CHA hairpins (H1, H2) for CHA reactions. Tech‐tetrahedron resides in a latched (inactive) state after cellular uptake. In telomerase‐rich tumor cytoplasm, primer elongation generates GGGTTA repeats that displace P6 through SDR, converting the construct into its unlatched (active) state and releasing free P6. (B) Formation of “hand‐in‐hand” aggregates. Bared P6 binds to the toehold of H1 on neighboring Tech‐tetrahedra, initiates catalytic hairpin assembly with H2 and covalently links successive tetrahedra, giving rise to spatially confined DNA nanoaggregates on the outer mitochondrial membrane. (C) Energy restriction by the nanoaggregate barrier. The polyanionic network sterically blocks metabolite trafficking across the mitochondrial surface, finally precipitating energy metabolic collapse. (D) Integrated cellular consequences. Guided by TPP, Tech‐tetrahedra accumulate on mitochondria, undergo telomerase‐mediated unlatching and aggregate formation, thereby (i) elevating reactive oxygen species, (ii) depleting ATP, (iii) suppressing nicotinamide‐nucleotide transhydrogenase (NNT) activity and (iv) up‐regulating polycystin‐1 (PC‐1). The combined bioenergetic crisis triggers cell apoptosis, culminating in marked inhibition of tumor growth in vivo.

Upon entering tumor cells with high telomerase activity, Tech‐tetrahedra (the plural form of “Tech″tetrahedron”) are guided by TPP to target and accumulate on the mitochondrial membrane. There, in the presence of telomerase (TE), the TP is elongated, generating repetitive GGGTTA sequences. These elongated sequences competitively bind to P5, triggering a SDR between P5 and P6 that leads to the dissociation of P6 (Figure 1A). The released P6 strand then binds to the toehold of H1, initiating a CHA cascade between H1 and H2. This cascade drives the “hand‐in‐hand” assembly of Tech‐tetrahedra into DNA nanoaggregates on the mitochondrial surface (Figure 1B). These DNA nanoaggregates form a physical barrier around the mitochondria that impedes mitochondrial‐cytoplasmic metabolite exchange, resulting in the inhibition of mitochondrial respiration and a decrease in ATP production (Figure 1C). Ultimately, a bioenergetic crisis that suppresses cell proliferation, migration, and invasion while promoting apoptosis. Transcriptomic analysis further indicated an involvement of polycystin‐1 and nicotinamide nucleotide transhydrogenase (NNT), suggesting a feedback loop. The therapeutic efficacy was subsequently validated in a mouse model (Figure 1D).

### 2.2 Establishment, Characterization and TE‐Response Performance of Tech‐Tetrahedron

**FIGURE 2:**
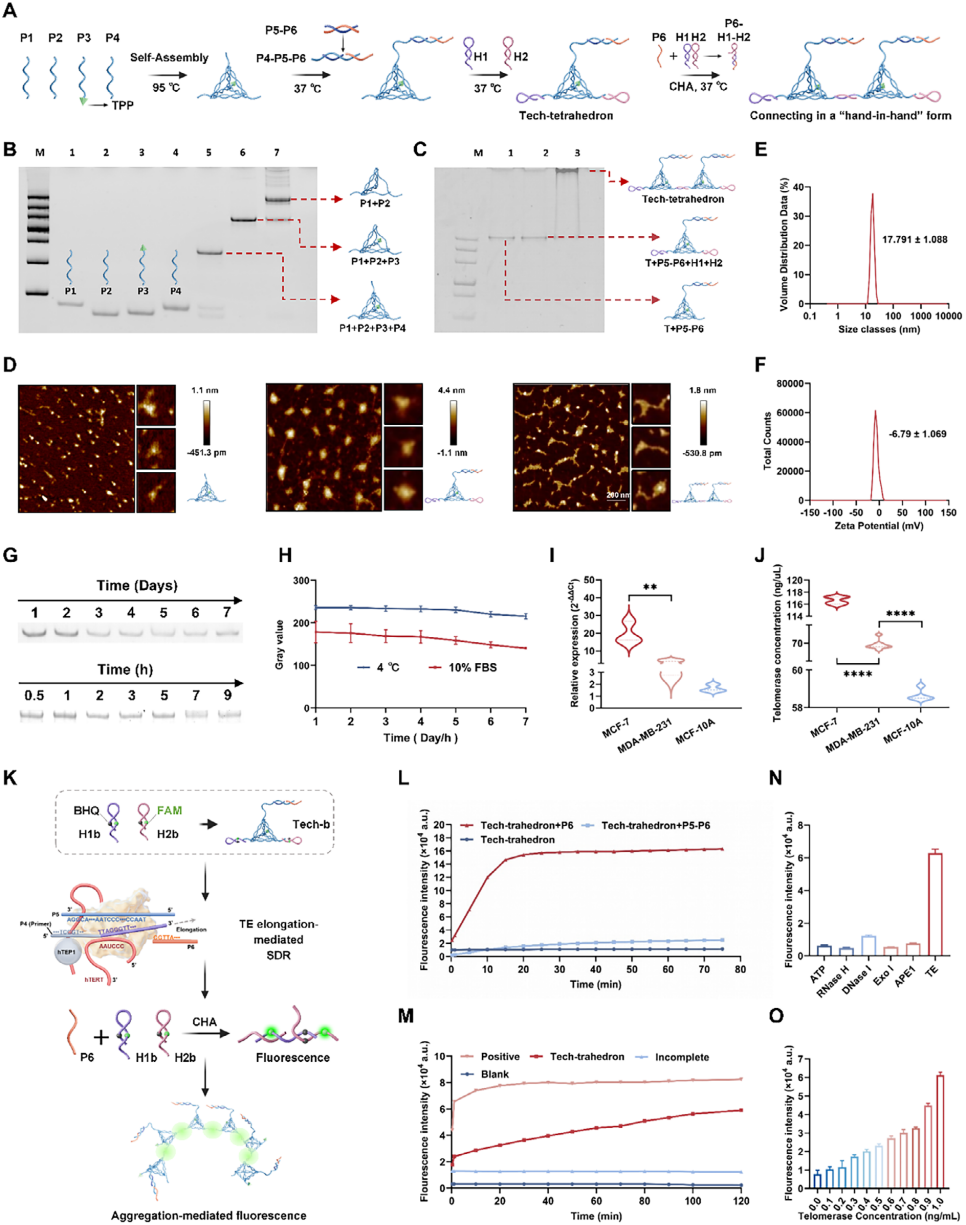
Operating Principle and characterization of Tech‐Tetrahedron. (A) Step‐wise assembly and working principle. Four DNA strands (P1–P4) form a tetrahedral framework; P5‐P6 creates a telomerase‐gated “latch” at one vertex; hairpins H1 and H2 are attached to other vertices. Telomerase elongation generates GGGTTA repeats that displace P6, triggering the strand displacement reaction (SDR). Released P6 activates catalytic hairpin assembly (CHA), connecting neighboring tetrahedra and generating fluorescence. (B) 12% native PAGE analysis tracking hierarchical assembly. Lane M: 50‐bp DNA ladder; lanes 1‐4: individual strands P1‐P4; lane 5: P1‐P6 TPP‐modified bara DNA tetrahedron; lane 6: P1 + P2 + P3 tetrahedron; lane 7: P1 + P2 complex. (C) 8% native PAGE confirmation of complete construct assembly. Lane 1: DNA tetrahedron ‐P5‐P6; lane 2: Tech‐tetrahedron; lane 3: connected Tech‐tetrahedrons. (D) Atomic force microscopy (AFM) topography images (scan area: 1 × 1 µm). Left to right: bare DNA tetrahedral, complete Tech‐tetrahedron, and DNA nanoaggregates. (E) Dynamic light scattering size distribution of Tech‐tetrahedra showing hydrodynamic diameter of 17.79 ± 1.08 nm. (F) Zeta‐potential distribution (−6.79 ± 1.06 mV) of Tech‐tetrahedron. (G) PAGE‐based stability assay following incubation in PBS (4 °C) or 10% FBS (37 °C) for 0–7 days. (H) Densitometric analysis of gel band intensities. (I) Relative expression levels of the TERT gene in three cell types, as determined by PCR analysis. (J) Telomerase activity in corresponding cell lines determined by TRAP‐ELISA. (K) Molecular mechanism illustrating telomerase extension‐mediated SDR and CHA amplification yielding fluorescence output (FAM/BHQ quencher pair) and inter‐tetrahedral aggregation. (L) Real‐time fluorescence kinetics comparing the Tech‐tetrahedron + P6 with control lacking Tech‐tetrahedron alone. Excitation/emission wavelengths: 488/520 nm. (M) Fluorescence generation of different groups in the presence of telomerase extracted from MCF‐7; Positive: extra P6; Incomplete: Tech‐tetrahedron without P6; Blank: PBS. (N) Fluorescence response intensity of the Tech‐tetrahedron upon the addition of other biological enzymes. (O) Calibration curve for telomerase quantification (concentration range: 0–3 nM). All error bars represent mean ± SD (n = 3 unless otherwise indicated).

Tech‐tetrahedron was first constructed using four DNA chains (P1‐P4) through a stepwise annealing process (Table S1), followed by the hybridization of thermally annealed hairpins (H1 and H2) (Table S1) to their respective vertices (P1 and P2). Concurrently, a telomerase‐gated latch, formed by pre‐hybridizing strands P5 and P6, was attached to the P4 vertex (Figure 2A). The successful synthesis at each stage was verified by 5% native polyacrylamide gel electrophoresis (PAGE). As expected, the electrophoretic mobility decreased progressively with the addition of each new component, consistent with increases in molecular mass and structural complexity (Figure 2B). A chain‐exclusion experiment confirmed that all four core strands (P1‐P4) were indispensable for the formation of the intact tetrahedron, as the final product band was absent when any single strand was omitted (Figure S1). The incorporation of P6 resulted in the lowest migration rate, indicating that its successful integration into the final construct (Figure 2C). Structural characterization by atomic force microscopy (AFM) confirmed the formation of well‐defined tetrahedral structures. While the small and dynamic hairpins were not visible at the vertices, the characteristic rigid tetrahedral morphology was evident. Upon addition of P6, AFM imaging revealed multiple Tech‐tetrahedra connecting in a “hand‐in‐hand” manner, forming elongated, band‐shaped nanoaggregates (Figure 2D). Dynamic light scattering (DLS) further corroborated the successful assembly, showing an increased hydrodynamic diameter (17.79 ± 1.09 nm) and a negative zeta potential (−6.79 ± 1.07 mV) for the Tech‐tetrahedron (Figure 2E,F, Figure S2). To evaluate the structural and functional stability of the Tech‐tetrahedron, we first examined its storage stability in 1× TM buffer at 4°C, where PAGE analysis showed that the construct remained well preserved for up to 4 days (Figure 2G). We then extended the analysis to biologically relevant conditions, including 10% fetal bovine serum (FBS), 20% FBS, 50% FBS, DMEM containing 10% FBS, and cell lysate at 37°C. Native PAGE revealed gradual time‐dependent attenuation in these complex media, indicating partial degradation under nuclease‐containing conditions (Figure 2H, Figure S3). Nevertheless, the construct remained detectable during the early time window relevant to the cellular experiments, and lysate‐pretreated samples retained measurable activation capability despite progressive signal decline. These findings indicate that the Tech‐tetrahedron maintains sufficient short‐term structural and functional integrity for intracellular activation and mitochondrial perturbation. Inaddition, comparison with a linear double‐hairpin construct carrying the same TPP modification and CHA‐responsive sequence but lacking the tetrahedral scaffold showed reduced nuclease resistance, weaker mitochondrial localization, and attenuated downstream biological effects, supporting the tetrahedral framework as a structurally advantageous scaffold in this system (Figures S4–S6; Table S2). Given the system's reliance on telomerase (TE) as a trigger, telomerase expression was validated in various cell types. Telomerase reverse transcriptase (TERT), a key component of telomerase activity and expression, was first amplified by PCR in three cell lines: MCF‐7, MDA‐MB‐231, and MCF‐10A. Telomerase proteins levels were quantified by ELISA. The relative expression levels in MCF‐7 cells were 4.75‐ and 11.94‐fold higher than those in 231 and 10A cells, respectively (Figure 2I, Figure S7). Following standard curve analysis, actual expression levels of TE were assessed in three cell types (Figure S8). TE protein expression was 116.60 ng/uL in MCF‐7, which was 1.67‐fold higher than in 231 cells and 1.99‐fold higher than in MCF‐10A cells (Figure 2J, Figure S9). Although telomerase expression in MDA‐MB‐231 cells was lower than that in MCF‐7 cells, it was still significantly different from that in MCF‐10A cells. Overall, telomerase expression and activity in breast cancer tumor cells were significantly higher than in normal breast epithelial cells.

Then the core functionality of the CHA reaction and the TE response was evaluated next. Hairpins H1b and H2b, labeled with fluorophore (FAM) and quencher (BHQ), exhibited a significant increase in fluorescence upon addition of P6, confirming the CHA reaction's successful initiation and efficacy (Figure S10). When integrated into the Tech‐tetrahedron, the addition of P6 induced a rapid fluorescence increase, plateauing within 20 min, demonstrating efficient inter‐tetrahedron linking (Figure 2K,L). The reaction conditions of the Tech‐tetrahedron were optimized further, including the vertex linker sequence, hairpin‐to‐tetrahedron ratio, and hairpin stem length, achieving maximal signal under a 2:1 molar ratio with H1 and H2 stem lengths of 15 and 14 nucleotides, respectively (Figures S11–S13, Table S1). The performance of the tech‐tetrahedron in response to TE was then investigated. TE was first extracted from the three cell types using the CHAPS lysis buffer method and the selectivity of the responsive assembly in tumor cells was assessed against other key biomolecules. FAM‐ and BHQ‐labeled Tech‐tetrahedra were incubated with ATP, ribonuclease H (RNase H), deoxyribonuclease I (DNase I), exonuclease I (Exo I), apurinic/apyrimidinic endonuclease 1 (APE1), and TE. A significant fluorescence increase was observed exclusively in the presence of TE, with signal intensities 5.380–13.468 times higher than that of other biomolecules, demonstrating good resistance and high specificity to potential interferents. For comparison, the fluorescence intensity of Tech‐tetrahedra treated with TE and direct P6 mediation was detected simultaneously. The kinetics of the TE‐triggered response were gradual, showing a continuous increase over 2 h without a clear plateau, in contrast to the rapid saturation seen with direct P6 addition (Figure 2 M,N), although the fluorescence intensity at the endpoint mediated directly by P6 was 1.396 times higher than that mediated by telomerase. This difference can be attributed to the time‐consuming process of TE‐mediated primer elongation. Finally, the fluorescence response showed a positive correlation with increasing TE concentration, confirming its potential for quantitative detection (Figure 2O).

### 2.3 Intracellular Assembly and Mitochondrial Targeting of Tech‐Tetrahedron

**FIGURE 3:**
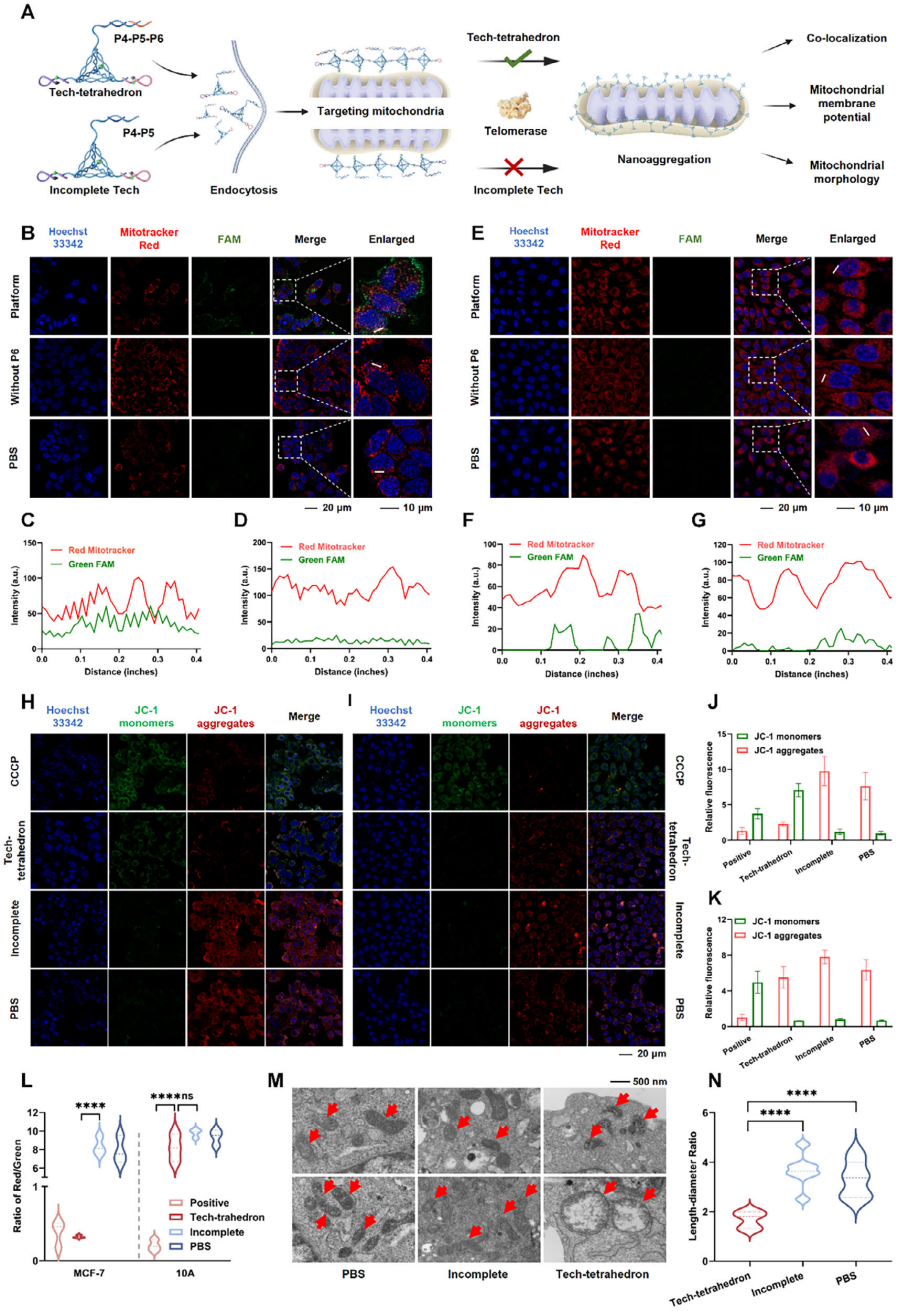
Intracellular Assembly and Mitochondrial Targeting of Tech‐tetrahedron. (A) Working principle of the two groups. Following endocytosis, TPP‐decorated Tech‐tetrahedra accumulate on the outer mitochondrial membrane. The P6‐deficient construct (Incomplete‐Tech) cannot undergo aggregation. (B) Confocal microscopy images of MCF‐7 cells following 4‐h incubation with FAM‐labeled Tech‐tetrahedron, Incomplete‐Tech (Without P6), or PBS control. Blue: nuclei (Hoechst 33342); red: mitochondria (MitoTracker Red); green: Tech‐tetrahedra (FAM). Boxed regions are magnified in adjacent panels. (C,D) Fluorescence intensity line profiles corresponding to white lines in (B). (E) Corresponding confocal images obtained from telomerase‐low MCF‐10A cells. Scale bars as in (B). (F,G) Line‐scan analyses for conditions shown in (E), confirming minimal colocalization in telomerase‐low cells. (H,I) JC‐1 staining assessment of mitochondrial membrane potential (MMP). Representative images of MCF‐7 (H) and MCF‐10A (I) cells treated with protonophore CCCP (positive control), Tech‐tetrahedron, Incomplete‐Tech, or PBS control. Red: JC‐1 aggregates (high ΔΨm); green: JC‐1 monomers (low ΔΨm). Scale bars: 25 µm. (J,K) Quantitative analysis of red and green JC‐1 fluorescence intensities for treatments shown in (H) and (I). (L) Red‐to‐green fluorescence intensity ratios in MCF‐7 and MCF‐10A cells. (M) Transmission electron microscopy images of mitochondrial ultrastructure following the indicated treatments. Red arrowheads indicate swollen mitochondrial matrices and disrupted cristae in Tech‐tetrahedron‐treated cells. (N) Statistical analysis of mitochondrial length‐to‐width ratios derived from TEM sections. Statistical significance determined by one‐way ANOVA. All error bars represent mean ± SD unless otherwise indicated. ns: not significant. ****p < 0.0001.

Having established its in vitro performance, the intracellular assembly and mitochondrial targeting of the Tech‐tetrahedron were proceeded to be validated. Given that nanoscale DNA constructs are predominantly internalized via endocytosis, whether the internalized Tech‐tetrahedron can escape endo‐lysosomal compartments was first assessed. Time‐dependent colocalization analysis with LysoTracker revealed an initial overlap between the FAM‐labeled construct and lysosomal signals, which progressively decreased over time (Figure S14). This spatial divergence suggests that a fraction of the internalized Tech‐tetrahedron undergoes endo‐lysosomal escape and becomes available in the cytosol, enabling interaction with intracellular targets such as telomerase. In addition, cytosolic access of the construct was functionally confirmed by telomerase inhibition using BIBR1532. Under telomerase inhibition, both the downstream assembly signal and the associated mitochondrial dysfunction were markedly attenuated (Figure S15), thereby providing functional evidence that a cytosol‐accessible fraction of the internalized Tech‐tetrahedron escapes endo‐lysosomal sequestration and is processed by endogenous telomerase. As designed, upon entering telomerase‐high MCF‐7 cells, the TPP‐guided Tech‐tetrahedra are expected to accumulate on mitochondria, where TE‐triggered primer elongation leads to P6 release and subsequent “hand‐in‐hand” nanoaggregate formation, potentially disrupting mitochondrial function (Figure 3A). Confocal laser scanning microscopy (CLSM) confirmed this mechanism. In MCF‐7 cells treated with the complete Tech‐tetrahedron (labeled with FAM), strong colocalization was observed between the green fluorescence of the tetrahedra and the red fluorescence of MitoTracker Red‐stained mitochondria. In contrast, cells treated with the incomplete Tech‐tetrahedron (lacking P6) showed minimal colocalization (Figure 3B–D, Figure S16). To further verify that the mitochondrial localization was mediated by TPP rather than indirect intracellular association, a TPP‐free construct and a mitochondrial depolarization model were examined. Both removal of TPP and dissipation of mitochondrial membrane potential markedly weakened mitochondrial localization of the Tech‐tetrahedron (Figure S17), indicating that its accumulation is TPP‐dependent and membrane‐potential‐sensitive, consistent with direct mitochondrial anchoring. Notably, two distribution patterns were observed: discrete spots and large aggregated clusters. In areas with aggregates, the mitochondrial red fluorescence was dramatically reduced or absent, suggesting local membrane potential loss and dysfunction (Figure S18). We hypothesize that local concentration gradients dictate assembly efficiency, with higher concentrations promoting aggregation and more severe damage. As expected, in telomerase‐low MCF‐10A cells, negligible green fluorescence was observed around mitochondria across all treatment groups, confirming the telomerase‐dependence of the assembly process (Figure 3E–G).

To assess functional consequences, we measured mitochondrial membrane potential (MMP) using the JC‐10 assay, in which JC‐10 forms red‐fluorescent aggregates under high MMP conditions and is converted to green‐fluorescent monomers when the MMP is compromised. Then MCF‐7 and MCF‐10A cells were treated with CCCP (positive control for MMP loss), PBS, incomplete Tech‐tetrahedron, or complete Tech‐tetrahedron for 8 h. The results showed that Tech‐tetrahedron treatment significantly decreased the red/green fluorescence ratio, indicative of MMP loss, to a level comparable to the CCCP group. This effect was not observed in cells treated with PBS or the incomplete tetrahedron, which exhibited decreases of 24.373‐fold and 25.886‐fold, respectively. MCF‐10A cells showed no significant changes in MMP across treatment groups, underscoring the specificity for telomerase‐positive cells (Figure 3H–L). These results demonstrate that Tech‐tetrahedra specifically compromise MMP in telomerase‐positive MCF‐7 cells.

Further evidence came from transmission electron microscopy (TEM), which revealed pronounced mitochondrial swelling, shortening (decreased length/diameter (L/D) ratio), and cristae disruption in Tech‐tetrahedron‐treated MCF‐7 cells compared to controls (Figure 3 M, Figure S19). Quantitative analysis revealed that the L/D ratio decreased by 2.172‐fold and 1.968‐fold compared to those in the incomplete Tech‐tetrahedron and PBS groups, respectively (Figure 3N). In additionally, we observed severe mitochondrial swelling and membrane rupture in a subset of organelles, which may be attributed to the highly dynamic nature of mitochondria. Collectively, these findings demonstrate that Tech‐tetrahedra successfully assemble on mitochondria in telomerase‐positive cells, forming aggregates that physically and functionally compromise mitochondrial integrity.

### 2.4 Tech‐tetrahedron Disrupts Mitochondrial Energy Metabolism

**FIGURE 4:**
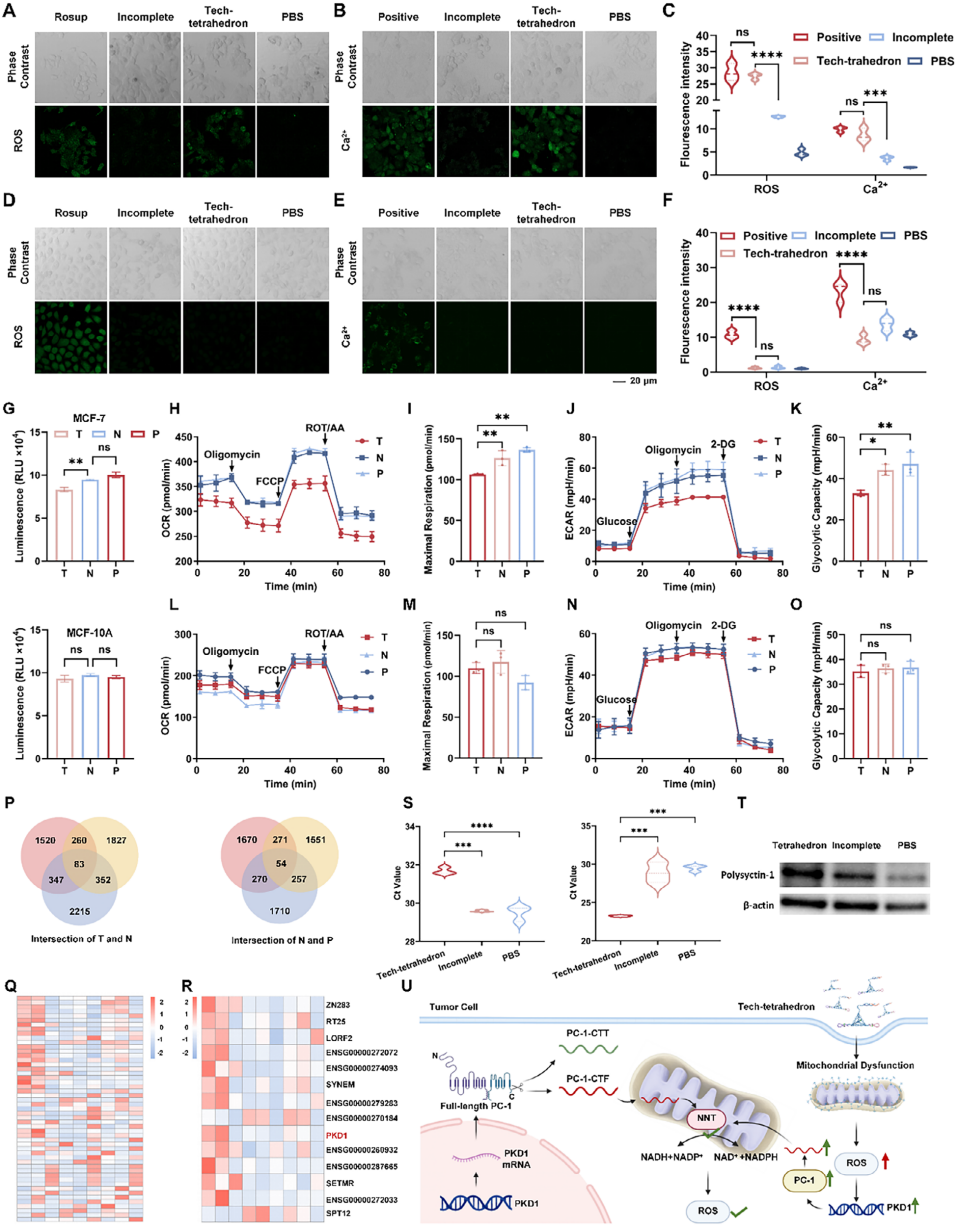
Tech‐tetrahedron Disrupts Mitochondrial Energy Metabolism. (A) Intracellular reactive oxygen species (ROS) generation visualized using DCFH‐DA fluorescence (green) in MCF‐7 cells following 8‐h treatment with ROS inducer Rosup (positive control), Tech‐tetrahedron, P6‐deficient Incomplete‐Tech, or PBS control. Upper panel: phase‐contrast images. (B) Cytosolic Ca^2^^+^ levels measured using Fluo‐4 AM fluorescence (green) in MCF‐7 cells under identical treatment conditions as shown in (A). (C) Quantitative analysis of mean fluorescence intensities for ROS and Ca^2^^+^ measurements in MCF‐7 cells. (D,E) Corresponding analysis of intracellular ROS generation (D) and cytosolic Ca^2^^+^ levels (E) in MCF‐10A cells under identical treatment conditions. (F) Quantitative analysis for ROS and Ca^2^^+^ measurements in MCF‐10A cells. (G) Cellular ATP levels determined in MCF‐7 and MCF‐10A cells with different treatments. Tech‐tetrahedron treatment significantly reduces ATP levels. T: Tech‐tetrahedron; N: Incomplete; P: PBS. (H) Mitochondrial respiration assessment in MCF‐7 cells. OCR were recorded following injections of oligomycin, FCCP, and rotenone/antimycin A (ROT/AA). (I) Quantitative analysis of maximal respiration capacity extracted from data shown in (H). (J) Extracellular acidification rates (ECAR) measured by Glycolysis Stress Test in MCF‐7 cells following sequential injections of glucose, oligomycin, and 2‐deoxyglucose (2‐DG). (K) Glycolytic capacity derived from (J). (L‐O) Corresponding metabolic analysis in telomerase‐low MCF‐10A cells. (M) Quantitative analysis of maximal respiration capacity (N), and extracellular acidification rates (O). T: Tech‐tetrahedron; N: Incomplete; P: PBS. (P) RNA‐sequencing analysis. Left panel: Venn diagram showing overlap of differentially expressed genes (DEGs) between Tech‐tetrahedron vs. Incomplete‐Tech (T vs. N) and Tech‐tetrahedron vs. PBS (T vs. P) comparisons. Right panel: corresponding overlap analysis for N vs. P comparison. (Q) Heatmap displaying the top 51 upregulated DEGs common to both comparison sets. The color scale represents z‐score‐normalized log_2_‐fold changes. (R) Heatmap displaying the top 14 DEGs common to both comparison sets. (S) Quantitative RT‐PCR validation of PKD1 transcript levels. (T) Immunoblot analysis of Polycystin‐1 (PC‐1) protein levels in cell lysates from indicated treatment groups. β‐actin serves as a loading control. (U) Proposed molecular mechanistic model. All the data represent mean ± SD. **p < 0.01, ****p < 0.0001, ***p < 0.001; statistical analysis by one‐way ANOVA with Tukey's multiple comparison test.

Mitochondrial dysfunction typically manifests through elevated reactive oxygen species (ROS), disrupted calcium homeostasis, and impaired ATP production. Thus, we systematically evaluated these parameters to comprehensively assess the bioenergetic crisis induced by Tech‐tetrahedron. MCF‐7 and MCF‐10A cells were treated with Tech‐tetrahedra, incomplete Tech‐tetrahedra, or PBS for 8 h, followed by staining with DCFH‐DA and Rhod‐2 AM to detect the intracellular ROS and free Ca^2^^+^ levels, respectively. In MCF‐7 cells, treatment led to a substantial increase in both ROS and cytosolic Ca^2^^+^ levels, comparable to positive controls, while control treatments (PBS and incomplete tetrahedron) showed no such effect (Figure 4A‐C, Figures S20 and S21). In contrast, MCF‐10A cells showed no significant elevation in either ROS or Ca^2^^+^ levels across the treatment groups, with responses markedly different from those of the positive controls (Figure 4D–F; Figures S22 and S23). Consistent with this, cellular ATP levels were significantly reduced in Tech‐tetrahedron‐treated MCF‐7 cells. As anticipated, MCF‐10A cells exhibited no significant changes in ATP levels across all groups (Figure 4G, Figure S24). These findings suggest that Tech‐tetrahedron‐induced mitochondrial dysfunction is likely due to impaired mitochondrial respiratory function, resulting disrupt calcium homeostasis and promote oxidative stress.

To further define the metabolic impairment, we analysed the oxygen consumption rate (OCR) and extracellular acidification rate (ECAR) to assess oxidative phosphorylation and glycolytic capacity, respectively. In MCF‐7 cells, Tech‐tetrahedron treatment substantially impaired mitochondrial function, reducing the maximum respiratory capacity by 15.664% and 21.912% compared to the incomplete Tech‐tetrahedron and PBS treatments, respectively (Figure 4H,I). Basal respiration decreased by 10.866% and 13.304%, while ATP‐linked respiration declined by 20.523% and 19.191%, respectively (Figure S25), confirming significant inhibition of oxidative phosphorylation and aligning with earlier measurements of reduced ATP levels. Notably, glycolytic capacity was also impaired in Tech‐tetrahedron‐treated MCF‐7 cells, with maximum glycolytic capacity reduced by 25.407% and 29.951% relative to the incomplete tetrahedron and PBS controls (Figure 4J,K). Basal glycolysis similarly decreased by 24.797% and 26.216%, respectively, whereas glycolytic reserve capacity remained unchanged (Figure S26). In contrast, no significant alterations in oxidative phosphorylation or glycolytic parameters were observed in MCF‐10A cells across treatment groups (Figure 4L–O, Figures S27 and S28). Together, these data demonstrate that the Tech‐tetrahedron selectively disrupts both mitochondrial respiration and downstream metabolic adaptation in MCF‐7 cells.

To determine whether this effect depends specifically on ordered CHA‐mediated aggregate formation rather than nonspecific mitochondrial localization or general mitochondrial stress, we introduced a mutant hairpin control (mut‐CHA) that retained the same overall composition and mitochondrial targeting capability but was unable to undergo the designed ordered aggregation. Importantly, mut‐CHA caused markedly weaker mitochondrial membrane potential loss and EdU suppression than the complete Tech‐tetrahedron (Figure S29, Table S3), indicating that the severe mitochondrial and antiproliferative effects require higher‐order aggregate formation rather than merely the presence of TPP‐modified nucleic acid components on mitochondria.

To further probe whether the mitochondrial defect reflects restricted substrate utilization rather than a nonspecific stress response, we performed substrate‐dependent OCR analysis using pyruvate, succinate plus rotenone, and glutamate as respiratory fuels (Figure S30). Across all tested substrates, the complete Tech‐tetrahedron caused a broad suppression of maximal respiration, ATP‐linked OCR, and ADP‐linked OCR (Figure S31). Although the incomplete and mutant controls showed minor, substrate‐dependent baseline inhibition, the fully assembled construct consistently produced a much stronger reduction in mitochondrial respiratory competence. These results suggest that the Tech‐tetrahedron does not merely trigger generalized mitochondrial stress, but more profoundly interferes with mitochondrial metabolic flux and substrate‐supported respiration.

To explore the molecular underpinnings, we conducted transcriptome sequencing. Differential gene expression analysis, controlling for non‐specific effects by comparing the Incomplete‐Tech group with the PBS group, identified 51 genes specifically responsive to complete aggregate formation (Figure 4P,Q, Tables S4–S6). Then, we specifically isolated the PKD1/NNT axis based on three criteria: significant differential expression (Figure 4R), functional correlation with mitochondrial stress, and strict phenotypic alignment with our observed ROS elevation and ATP depletion. PKD1 encodes polycystin‐1 (PC‐1), a large membrane glycoprotein that, upon cleavage, generates C‐terminal fragments (PC1‐CTF) that can translocate to the mitochondria [38, 39]. Biologically, the C‐terminal tail of PC‐1 specifically binds to nicotinamide nucleotide transhydrogenase (NNT) located on the inner mitochondrial membrane [40]. This interaction is crucial for maintaining mitochondrial energy metabolism and redox stability. Specifically, NNT facilitates the transhydrogenation between NADH and NADP^+^ to generate NADPH, which is essential for supporting cellular antioxidant defenses [41].

qPCR and western blot analyses confirmed upregulation of PKD1 mRNA and PC‐1 protein levels in Tech‐tetrahedron‐treated MCF‐7 cells (Figure 4S,T). In the context of our system, we interpret this upregulation of PC‐1 as a compensatory cellular response attempting to restore redox buffering via NNT in the face of the bioenergetic crisis induced by the Tech‐tetrahedron. However, the persistent elevation of ROS levels indicates that this compensatory mechanism is overwhelmed. The coexistence of increased PC‐1 (compensatory response) and elevated ROS (ongoing damage) supports our hypothesis that the tetrahedral aggregates create an insurmountable physical barrier, leading to a sustained bioenergetic crisis (Figure 4U).

### 2.5 Tech‐Tetrahedron Suppresses Tumor Cell Proliferation, Migration, and Invasion

**FIGURE 5:**
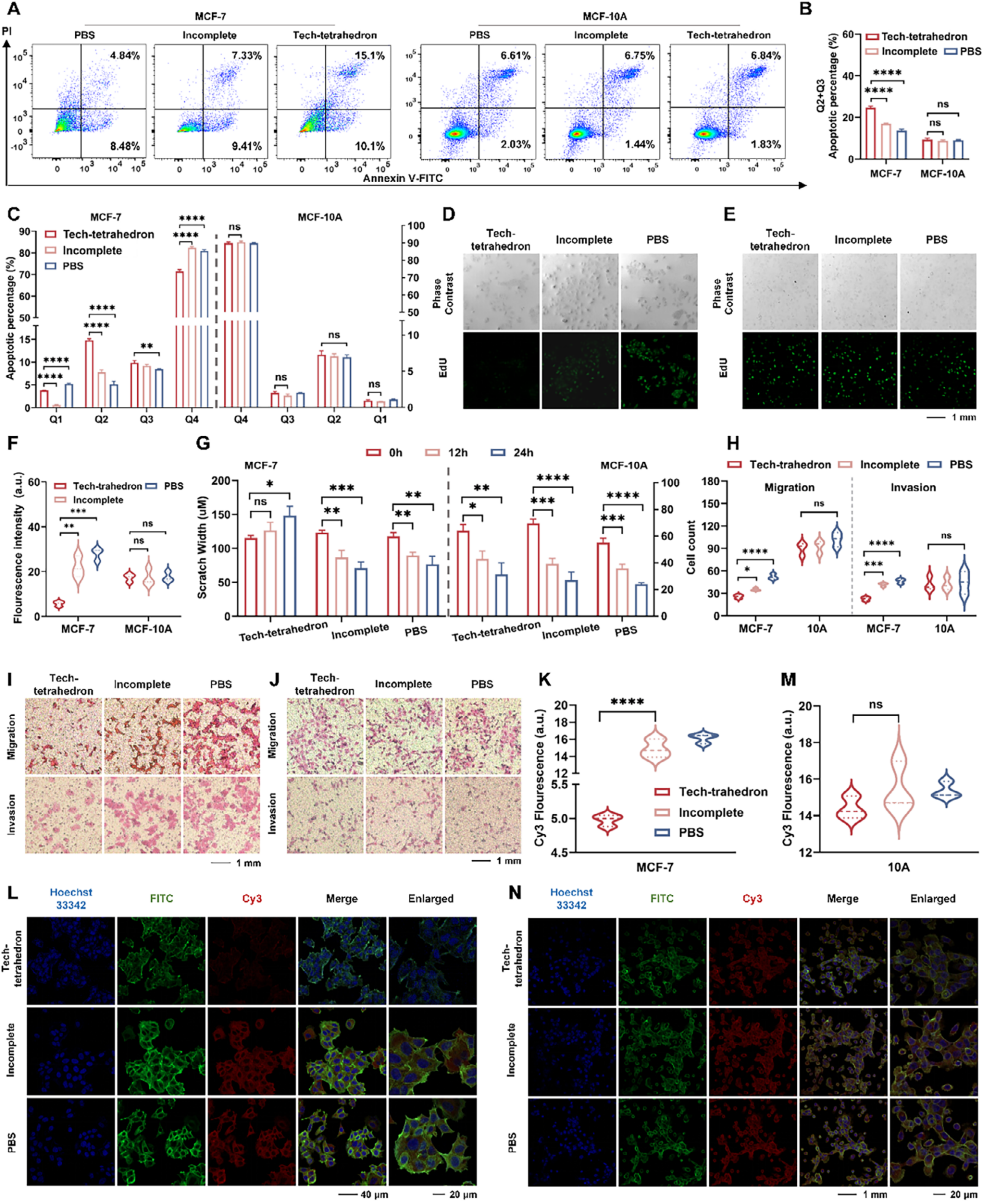
Tech‐tetrahedron Suppresses Tumor Cell Proliferation, Migration, and Invasion. (A) Representative Annexin V‐FITC/propidium iodide flow cytometry plots of MCF‐7 (left) and MCF‐10A (right) cells following 24‐h exposure to PBS control, P6‐deficient Incomplete‐Tech, or complete Tech‐tetrahedron (T). Q1: necrotic; Q2: late apoptotic; Q3: early apoptotic; Q4: viable cells. (B) Percentage of total apoptotic cells (Q2 + Q3) quantified from (A). (C) Detailed quadrant statistics for MCF‐7 and MCF‐10A cells. (D,E) Cell proliferation assessment. Phase‐contrast (upper) and EdU‐labeled DNA synthesis (green, lower) images of MCF‐7 (D) and MCF‐10A (E) cultures following 8‐h treatment. (F) Violin plots of EdU fluorescence intensities in both MCF‐7 and MCF‐10A cell lines. (G) Quantification of scratch widths from Scratch‐wound assay showing remaining gap width in MCF‐7 and MCF‐10A at 12 and 24 h with statistical analysis as indicated. (H) Transwell migration and invasion assay. Number of migratory or invasive cells per field after 48 h. (I,J) Representative crystal violet‐stained transwell inserts showing reduced migration and invasion in MCF‐7 (I) but not MCF‐10A (J) cells upon Tech‐tetrahedron treatment. (K,M) Mean cytoplasmic cortactin (Cy3) fluorescence intensities in MCF‐7 (K) and MCF‐10A (M) cells. (L,N) Confocal immunofluorescence of F‐actin (FITC‐phalloidin, green) and cortactin (Cy3, red) with nuclei counterstained using Hoechst 33342 (blue) in MCF‐7 (L) and MCF‐10A (N) cells. Dashed outlines indicate cell boundaries; magnified views shown at right. Statistical analysis performed by one‐way ANOVA followed by Tukey's multiple comparison test. ****p < 0.0001, ***p < 0.001, **p < 0.01, *p < 0.05; ns: not significant. Error bars represent mean ± SD.

To evaluate the therapeutic potential of Tech‐tetrahedron, we systematically assessed its effects on the key hallmarks of cancer cell behavior, including apoptosis, proliferation, migration, and invasion. Apoptosis was quantified using Annexin V‐FITC/propidium iodide staining, followed by flow cytometry. Tech‐tetrahedron treatment significantly increased the apoptosis rates in MCF‐7 cells to 24.67% compared to the control treatments, whereas MCF‐10A cells showed minimal apoptosis (9.29%) across all treatment groups (Figure 5A–C). This selective pro‐apoptotic effect demonstrates the specificity of Tech‐tetrahedron for telomerase‐positive cancer cells. Cell proliferation was assessed using an EdU incorporation assay to detect DNA synthesis in actively dividing cells. Tech‐tetrahedron treatment dramatically reduced EdU‐positive cells in MCF‐7 cultures, with green fluorescence intensity decreasing by 74.022% compared to incomplete Tech‐tetrahedron treatment (Figure 5D–F). In contrast, MCF‐10A cells showed no significant changes in proliferation across the treatment groups. These results confirmed that the Tech‐tetrahedron effectively promoted apoptosis and suppress proliferation, specifically in cancer cells.

Cell migration was evaluated using wound healing (scratch) and transwell migration assays with the scratch assay interpreted as a composite readout influenced by both migratory activity and cell viability. In the scratch assay, while control MCF‐7 cells progressively closed the wound gap, Tech‐tetrahedron‐treated cells showed delayed wound closure, with the scratch width remaining 28.671% wider after 24 h compared to the initial measurement (Figure 5G; Figure S32). Notably, numerous floating dead cells were observed in the Tech‐Tetrahedron treatment group because the scratch‐wound assay was not normalized for cell viability. This delayed closure should be interpreted as a combined effect of reduced cell motility and treatment‐associated cytotoxicity, rather than as a pure measure of migration inhibition. MCF‐10A cells demonstrated robust wound closure across all treatment groups, with the tech‐tetrahedron group achieving 51.083% closure by 24 h, comparable to the control treatments (Figure 5G; Figure S33). Transwell migration assays provided complementary evidence supporting a motility‐suppressive effect of Tech‐tetrahedron. After 48 h of treatment, significantly fewer MCF‐7 cells successfully migrated through the membrane in the Tech‐Tetrahedron group than in the control group (Figure 5H,I; Figure S34). MCF‐10A cells showed no treatment‐related differences in their migration capacity (Figure 5H,J, Figure S35). Similarly, transwell invasion assays with Matrigel‐coated membranes revealed that Tech‐tetrahedron treatment substantially reduced the invasive capacity of MCF‐7 cells, with markedly fewer cells penetrating the extracellular matrix barrier compared to the control group (Figure 5I; Figure S39). Again, the MCF‐10A cells showed no significant differences across the treatment groups (Figure 5J; Figure S36).

To understand the molecular basis of the impaired migration and invasion, we examined the actin cytoskeleton, which is fundamental to cell motility. Cells were stained for nuclei (Hoechst 33342, blue), F‐actin (FITC‐phalloidin, green), and cortactin (anti‐cortactin antibody, red) following 8‐h treatments. CLSM imaging revealed that while F‐actin levels remained unchanged, cortactin expression was significantly reduced in tech‐tetrahedron‐treated MCF‐7 cells compared to controls (Figure 5K,L; Figure S37). Cortactin is a critical actin‐binding protein that promotes actin polymerization and stabilizes branched actin networks that are essential for lamellipodia formation and cell motility. Its downregulation provides a molecular explanation for defects observed in cell migration and invasion. MCF‐10A cells showed no significant alterations in F‐actin or cortactin expression across the treatment groups (Figure 5M,N; Figure S38), which was consistent with their maintained migratory capacity. The reduction in cortactin expression and impaired lamellipodia formation may result from ATP depletion, as shown in our earlier metabolic studies. Actin remodeling requires substantial ATP for polymerization, myosin activity, and maintaining cellular protrusions. The bioenergetic crisis caused by Tech‐tetrahedron treatment likely disrupts these processes, leading to defective migration and invasion. Together, these findings show that the tetrahedron exerts anti‐cancer effects by promoting apoptosis, inhibiting proliferation, and suppressing metastasis through disruption of mitochondrial metabolism and cytoskeletal organization.

### 2.6 In Vivo Therapeutic Efficacy

**FIGURE 6:**
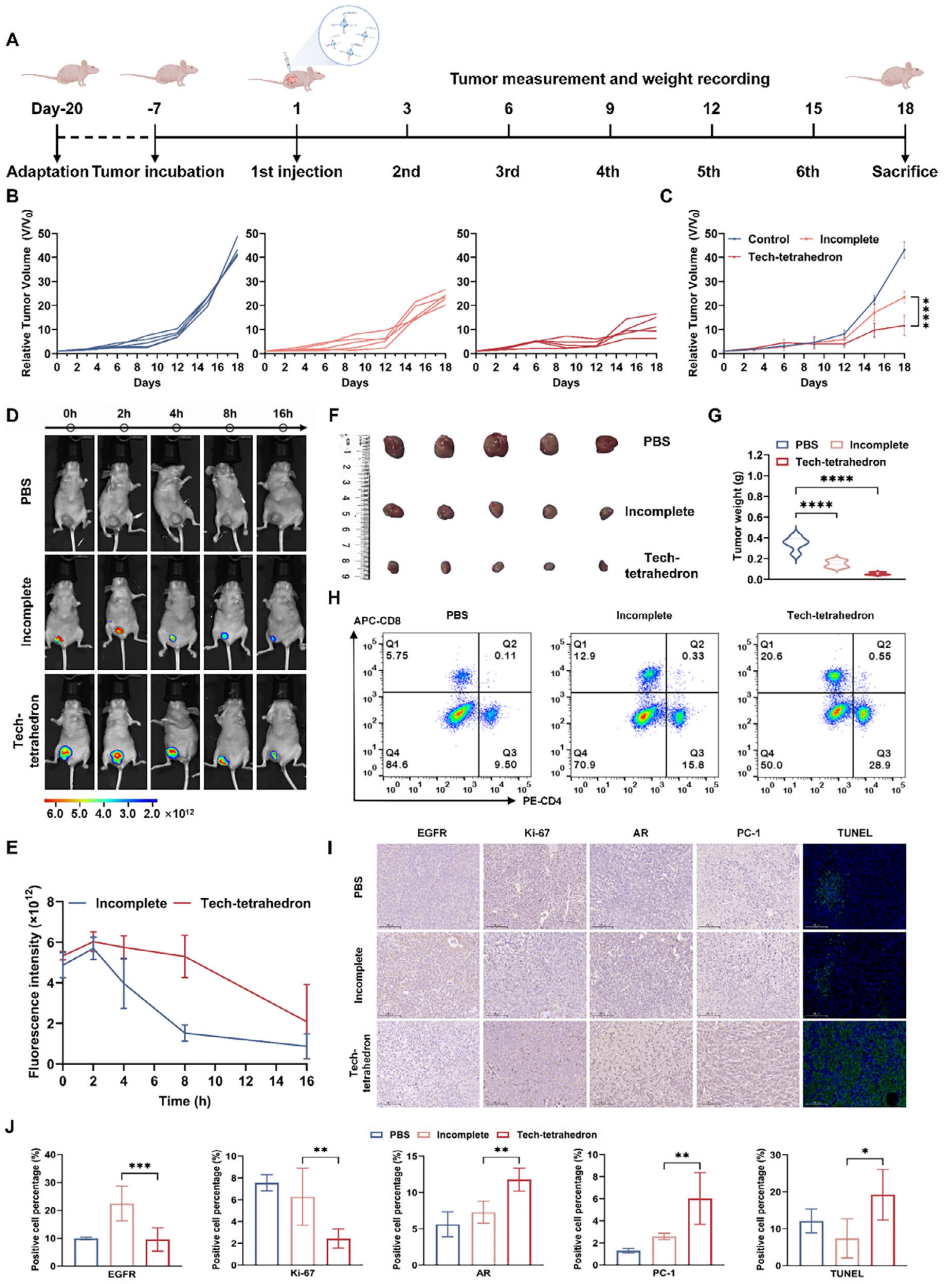
In Vivo Therapeutic Efficacy. (A) Treatment schedule. Female BALB/c nude mice were acclimatized for 7 days, subcutaneously inoculated with MCF‐7 cells (day ‐7), and randomly assigned to three groups (n = 5). Intra‐tumoral injections of Tech‐tetrahedron, Incomplete‐Tech (P6‐deficient), or PBS were administered on days 1, 3, 6, 9, 12, and 15. Tumor volume and body weight were recorded every 3 days; animals were sacrificed on day 18. (B) Individual tumor growth curves for the three groups (left, PBS; middle, Incomplete; right, Tech‐tetrahedron). (C) Mean relative tumor volume (RTV = V/V_0_) vs. time (mean ± SD, n = 5). Tech‐tetrahedron achieved 63.2% growth inhibition compared with PBS on day 18. (D) In vivo bioluminescence imaging of representative mice at the indicated time points after the 5th injection. (E) Photographs of excised tumors on day 18; scale bar in centimeters. (F) Tumor weights of three different treatment groups. (G) Flow cytometry analysis of splenic T cell subsets. (H) Quantification of splenic T cell populations (violin plots, n = 3). (I) Representative micrographs and quantitative histograms of day‐18 MCF‐7 tumors illustrate. From left to right: EGFR, Ki‐67, AR, PD‐L1, and TUNEL staining; boxed regions are magnified in adjacent panels. (J) Quantitative histograms of day‐18 MCF‐7 tumors illustrate. Data are mean ± SD; n = 5; **p < 0.01, ***p < 0.001 by one‐way ANOVA with Tukey's post‐test.

To assess the therapeutic potential and safety profile of Tech‐tetrahedron in vivo, subcutaneous MCF‐7 tumor xenografts were established in immunodeficient mice. Tumor‐bearing mice were randomly allocated to three groups (n = 5 per group) and received intra‐tumoral injections of Tech‐tetrahedron, Incomplete‐tetrahedron, or PBS control. The treatment was administered twice weekly for 18 days according to the schedule depicted in Figure 6A. Throughout the 18‐day treatment period, all mice in the three treatment groups maintained stable body weights, indicating good tolerance to the administered formulations (Figure S39). Comprehensive safety assessment, including routine hematological analysis and serum biochemical parameters (liver and kidney function markers), revealed no significant abnormalities in any treatment group compared to PBS controls (Figures S40 and S41), suggesting minimal systemic toxicity of the Tech‐tetrahedron.

Tumor volumes were measured every three days using digital calipers, and the relative tumor growth curves demonstrated distinct treatment responses among groups (Figure 6B,C). Real‐time fluorescence imaging showed that these treatment responses coincided with prolonged intratumoral retention. The signal from Tech‐tetrahedron remained intense at 16 h, whereas Incomplete was largely cleared by 8 h (Figure 6D,E; Figure S42). The Tech‐tetrahedron group exhibited the most pronounced tumor growth inhibition throughout the treatment period. Visual inspection of excised tumors on day 18 clearly demonstrated substantial size reduction in the Tech‐tetrahedron‐treated group compared to the control groups (Figure 6F). Quantitative analysis of tumor weights at the experimental endpoint revealed that Tech‐tetrahedron treatment achieved a significant 63.15% reduction compared to PBS control and a 50.26% reduction compared to Incomplete‐tetrahedron (Figure 6G). When calculated as tumor growth inhibition (TGI) rate based on tumor volume progression, the Tech‐tetrahedron group achieved 63.16% growth suppression, significantly outperforming both Incomplete‐tetrahedron (TGI = 28.43%, p < 0.05) and PBS control groups.

To investigate the potential immunomodulatory mechanism underlying the anti‐tumor efficacy, T cell subpopulations were analyzed in the spleen at the experimental endpoint. Splenocytes were isolated, gated on CD3^+^ cells, and co‐stained with CD4‐PE and CD8‐APC antibodies for flow cytometric analysis. Tech‐tetrahedron treatment expanded the splenic CD3^+^ compartment to 91.2 %, compared with 73.6 % in the Incomplete group and 49.9 % in PBS controls. (Figure S43). Quadrant Q1 represents CD3^+^CD8^+^ cytotoxic T lymphocytes (CTLs), and quadrant Q3 represents CD3^+^CD4^+^ T helper cells (Th cells) (Figure 6H). Flow cytometry results demonstrated that Tech‐tetrahedron treatment markedly elevated the percentages of both CD3^+^CD4^+^ Th cells (38.19 ± 3.24% vs. 22.15 ± 2.87% in Incomplete‐tetrahedron) and CD3^+^CD8^+^ CTLs (46.38 ± 4.11% vs. 28.76 ± 3.55% in Incomplete‐tetrahedron) in the spleen (Figure S44). These results suggest that Tech‐tetrahedron may exerts part of its anti‐tumor effects through modulation of residual adaptive immune responses.

Histopathological analysis of day‐18 MCF‐7 xenografts identified the molecular basis for Tech‐tetrahedron's therapeutic superiority (Figure 6I). Immunohistochemistry showed reduced EGFR membrane expression, indicating inhibited EGFR‐driven proliferation (Figure S45). Ki‐67 labeling decreased to 2.431 ± 0.878% vs. 6.279 ± 2.616% in controls, confirming cell cycle arrest (Figure S46). Tech‐tetrahedron‐treated tumors exhibited higher AR nuclear positivity (11.769 ± 1.584%), suggesting hormone pathway involvement and possible prognostic benefit (Figure S47). PC‐1 expression increased significantly compared to controls, aligning with prior RNA‐seq and WB data (Figure S48). TUNEL assay confirmed apoptosis (19.197% positive nuclei, 2.6‐fold higher than controls) (Figure S49, Figure 6J). These findings demonstrate Tech‐tetrahedron inhibits EGFR signaling, reduces proliferation and metabolism, and induces cell death, achieving potent tumor suppression with low toxicity in vivo.

## 3 Conclusion

In this study, Tech‐tetrahedron was developed based on a trinity‐functionalized DNA tetrahedron to address fundamental challenges in artificial intracellular aggregation systems and realize mitochondrial energy restriction, demonstrating significant advantages across structural innovation, spatiotemporal control, and energy restriction mechanisms.

Structurally, Tech‐tetrahedron exploits DNA tetrahedra's tetrahedral geometry and mechanical resilience to overcome limitations of conventional DNA precursors. Its trinity‐functionalized design integrates navigation, enzymatic control, and self‐assembly functions into a biocompatible platform, featuring independently addressable vertices for enhanced stability and programmability in complex intracellular environments. For spatiotemporal control, Tech‐tetrahedron achieves precision subcellular regulation through telomerase‐responsive dual‐state switching between latched and unlatched conformations. Enzymatic gating enables temporal control while TPP modification ensures mitochondrial targeting specificity, resulting in “hand‐in‐hand” nanoaggregate formation at mitochondrial membranes under high telomerase conditions. Regarding energy restriction, Tech‐tetrahedron implements a comprehensive bioenergetic intervention by forming polyanionic barriers that disrupt mitochondrial‐cytoplasmic metabolite exchange. This achieved dual inhibition of aerobic respiration and glycolysis that leads to bioenergetic collapse with 24.67% increased apoptosis and 63.16% tumor growth inhibition, while transcriptomic analysis revealed polycystin‐1 upregulation and potential NNT modulation establishing a self‐reinforcing feedback loop.

In conclusion, Tech‐tetrahedron defines a distinct intervention logic that links endogenous signal sensing, organelle‐localized assembly, and structure‐driven dysfunction. By moving beyond the “DNA‐as‐carrier” model, this study proves that the programmed physical state of DNA nanostructures can serve as a potent biological regulator. This payload‐free, structure‐mediated strategy not only achieves efficient mitochondrial energy restriction and tumor inhibition but also expands the role of DNA nanotechnology from passive cargo delivery toward active, structure‐driven regulation of cellular fate.

There remain important open questions regarding the molecular permeation behavior of DNA aggregates in biological barriers, for which systematic quantitative data are still limited. Thus, future research needs to integrate multi‐scale computational models, high‐precision experimental platforms, and standardized permeability assessment guidelines to clarify the permeation laws of DNA aggregates in complex biological environments, and provide a theoretical basis for gene delivery, nanomedicine, and synthetic biology applications.

## 4 Experimental Section

### 4.1 Materials

All oligonucleotides (Table S1), purified via high‐performance liquid chromatography, were obtained from Sangon Biotech Co., Ltd. (Shanghai, China) and Hongxun Biotechnologies (Suzhou, China). Tris (hydroxymethyl) aminomethane (Tris), hydrochloric acid (HCl), NaCl, EDTA, and MgCl2 were purchased from Sangon Biotech Co., Ltd. (Shanghai, China). TM Buffer was obtained from Beyotime Biotech Co., Ltd. (Shanghai, China). Ethylene glycol tetraacetic acid (EGTA) was obtained from Sigma‐Aldrich (St. Louis, MO, USA). Human telomerase (TE) Quantitative Detection Kit (ELISA) was obtained from Ruixin Biotech Co., Ltd. (Hubei, China). Mito‐Tracker Deep Red FM, Fluo‐4 calcium ion detection kit, Reactive oxygen species detection kit, ATP detection kit, and Enhanced mitochondrial Membrane potential Detection kit (JC‐1) were obtained from Beyotime Biotech Co., Ltd. (Shanghai, China). Hoechst 33342‐ Trihydrocht was obtained from Invitrogen Biotech Co., Ltd. (Beijing, China). FITC Phalloidin was obtained from Yeasen Biotech Co., Ltd. (Shanghai, China). Oxygen Consumption Rate (OCR) Fluorometric Assay Kit and Extracellular acidification rate (ECAR) fluorescence assay kit were obtained from Elabscience Biotech Co., Ltd. (Hubei, China). Annexin V‐FITC/PI apoptosis detection kit and Yefluor 488 EdU Imaging Kit were obtained from Yeasen Biotech Co., Ltd. (Shanghai, China). SV Total RNA Isolation System was obtained from Promega Biotech Co., Ltd. (Madison, US), miRNAFirst Strand cDNA Synthesis (Stem‐loop Method) kit and TransScript All‐in‐One First‐Strand cDNA Synthesis SuperMix for qPCR (One‐Step gDNA Removal) were purchased from Sangon Biotech Co., Ltd. (Shanghai, China). FOXD4 Polyclonal Antibody and Polycystin‐1 Polyclonal Antibody were obtained from Invitrogen Biotech Co., Ltd. (Beijing, China). Goat Anti‐Rabbit IgG H&L (HRP) preadsorbed, Recombinant Anti‐Cortactin Antibody were obtained from Abcam Biotech Co., Ltd. (Cambridge, UK). Cy3‐conjugated Affinity Purified Goat Anti‐Rabbit IgG (H+L) was obtained from APExBIO Biotech Co., Ltd. (Houston, USA). A nested transwell with a 6‐well plate (PC membrane, 24 mm, 8.0 µm) was obtained from corning (New York, USA). DEME medium and FBS were acquired from VivaCell (Shanghai, China), and penicillin/streptomycin solution and PBS were purchased from Gibco (NY, USA). MCF‐10A cell medium was acquired from Pricella Biotech Co., Ltd. (Wuhan, China). MCF‐7, MDA‐MB‐231, and MCF‐10A cell lines were obtained from Meisen CTCC (Hangzhou, China).

### 4.2 Apparatus

Fluorescence spectra were recorded by the F‐7000 fluorescence spectrophotometer (Hitachi Co. Ltd., Japan). Native polyacrylamide gel electrophoresis (PAGE) was performed using an electrophoresis system (Bio‐Rad, CA, USA). The gels were imaged using the ChemiDoc XRS system (Bio‐Rad, CA, USA). Fluid‐mode atomic force microscopy (AFM) was performed using a Bruker MultiMode 8 AFM (Bruker, MA, USA) with a PEAKFORCE‐HIRS‐F‐A tip. TEM was performed using Tal os F200X (FEI, USA). Cells were cultured by CO2 cell culture (ESCO Celculture, Singapore) and counted by LUNA‐II (Logos Biosystems, Korea). CCK‐8 assay and ELISA was performed by Thermo Scientific Varioskan Flash (MA, USA). NanoDrop 2000 spectrophotometer (Thermo Fisher Scientific, USA). Flow cytometry was performed by BD FACSCanto II flow cytometer (BD, USA). The florescence staining of cells were recorded by a fluorescence microscope (Olympus, Japan). The reverse transcription quantitative polymerase chain reaction (RT‐qPCR) analysis was carried on by CFX96 real‐time system (Bio‐Rad, USA). The CLSM imaging was conducted utilizing a ZEISS LSM 880 (Zeiss, Germany). All animal experiments were approved by the Institutional Animal Welfare Ethics Committee and the Ethics Committee of the Army Medical University, and were conducted in accordance with the Guide for the Care and Use of Laboratory Animals (Approval Number: AMUWEC20225084).

### 4.3 Methods

#### 4.3.1 Construction and Characterization of Tech‐tetrahedron

Tech‐tetrahedron was built with a tetrahedral framework, two CHA hairpins (H1, H2), and the linear DNA branching (P5, P6) as a response to telomerase activity. The H1 and H2 (4 µM; 5 µL) were added to the TM buffer (20 mM Tris‐HCl; 50 mM MgCl2; pH 8.0), respectively, and denatured at 95 °C, then slowly cooled down to 4°C to construct the hairpin structure. P5 and P6 (4 µL; 5 µL) were mixed into the TM buffer and heated for 40 min at 37°C to construct the P5‐P6 complex. The S1, S2, S3, and S4 (4 µM; 5 µL) were mixed into the TM buffer and heated for 5 min at 95°C, 5 min at 75°C, 5 min at 55°C, 5 min at 37°C, 5 min at 25°C, and maintained at 4°C for at least 2 h to architect the tetrahedral framework. The synthesis process of Tech‐tetrahedron was verified utilizing 5% PAGE. Fluid‐mode atomic force microscopy (AFM) was performed using a Bruker MultiMode 8 AFM (Bruker, MA, USA) with a PEAKFORCE‐HIRS‐F‐A tip.

#### 4.3.2 Stability and Biological Resistance of the Tech‐Tetrahedron

The Tech‐tetrahedron solution was adjusted to a final concentration of 400 nM. Subsequently, an equal volume of 1× TM solution and 10% fetal bovine serum (FBS) were added separately. PAGE analysis was conducted following incubation at 4°C and 37°C for 0, 0.5, 1, 2, 5, 8, and 10 h for investigating the stability and biological resistance of Tech‐tetrahedron, respectively.

#### 4.3.3 Cell Culture and Telomerase Extraction

MCF‐7, MDA‐MB‐231, and MCF‐10A cells were cultured in high‐glucose Dulbecco's modified Eagles medium supplemented with 10% (v/v) FBS, 100 µg mL−1 streptomycin, and the cells were maintained at 37°C in a moist atmosphere containing 5% CO_2_. Three types of cells (1 × 10^6^) were collected in the exponential phase of growth and washed twice with ice‐cold PBS. Then, the collected cells were resuspended in 100 µL of ice‐cold (3‐((3‐cholamidopropyl) dimethylammonio)‐1‐propanesulfonate) (CHAPS) lysis buffer (0.5%(w/v) CHAPS, 10 mM Tris−HCl, pH 7.5, 1 mM MgCl2, 1 mM ethylene glycol tetraacetic acid (EGTA), 0.1 mM phenylmethylsulfonyl fluoride, 10% glycerol). The cell lysate was incubated on ice for 30 min and centrifuged at 13 780 g for 20 min at 4°C. The supernatant was collected and stored at −80°C for further use.

#### 4.3.4 PCR of the Telomerase Reverse Transcriptase (TERT)

The Spark easy cell RNA kit was used to rapidly extract the total RNA of the collected cells (the total number of cells was ≈1 ‐ 2 × 10^6^). The steps were carried out according to the operation manual, and then the nanodrop was used to determine the parameters of A260/A280 and A260/230 to determine whether the extraction was pure. For the miRNA assay, Reverse transcription was performed according to the operating instructions (miRNAFirst Strand cDNA Synthesis (Stem‐loop Method) kit), the cDNA concentration was determined again using Nanodrop. The TERT expression level was determined by PCR using TB Green Advantage qPCR Premix. GAPDH was utilized as the internal reference gene for normalization in the quantitative analysis.

#### 4.3.5 Human Telomerase ELISA Detection

Three types of cells were gently washed with cold PBS, then digested with trypsin, and collected by centrifugation at 1200 × g for 3 min. The collected cells were washed three times with cold PBS. 150‐200 µL of PBS was added to each 1×10^6^ cells for resuspension and the cells were disrupted by repeated freeze‐thawing (the volume of PBS could be reduced if the content was very low). The extract was centrifuged at 1500 × g for 10 min and the supernatant was taken for ELISA investigation of telomerase. The extract was added into the microplate wells pre‐coated with anti‐human telomerase (TE) antibody (solid‐phase antibody), followed by the addition of another HRP‐labeled anti‐human telomerase (TE) antibody (enzyme‐labeled antibody). After incubation 60 min at 37 °C and thorough washing to remove unbound components, substrate of HRP was added. After the action of the stop solution, the absorbance (OD value) is measured at a wavelength of 450 nm on an enzyme reader. By fitting the calibration curve, the concentration of human telomerase (TE) in the sample can be calculated.

#### 4.3.6 In Vitro Telomerase Extension Reaction

The telomerase extracts (20 µL) were mixed with 1 mM deoxynucleotide triphosphate (5 µL), 0.5 µM Tech‐tetrahedron (5 µL, H1 and H2 were modified with fluorescent moiety FAM), and an extension solution containing reaction buffer (20 mM Tris−HCl, pH 8.3, 1.5 mM MgCl_2_, 63 mM KCl, 0.005% Tween 20, 1 mM EGTA, and 0.1 mg/mL bovine serum albumin). The mixture was reacted at 37°C for 2 h, and the fluorescence intensity was detected during the reaction process as time changed.

#### 4.3.7 Colocalization Analysis

The cells were seeded into confocal dishes and cultured overnight (≈1 × 10^5^ cells per dish), then the medium was discarded and added 200 uL fresh medium, 500 µL incomplete Tech‐tetrahedra, and 500 µL Tech‐tetrahedra (1 µM incomplete Tech‐tetrahedra/Tech‐tetrahedra with two hairpins modified with FAM) separately. Incubated at 37°C for 2, 4, 6, and 8 h. After that all groups were incubated with a fresh medium containing 50 nM Tracker green for 30 min. Subsequently, the cells were stained by Hoechst (10 µg/mL) for another 15 min using a standard procedure. Finally, the samples were imaged by Confocal Laser Scanning Microscopy.

#### 4.3.8 Mitochondrial Membrane Potential (MMP) Analysis

MCF‐7 cells were seeded in confocal dishes (≈1 × 10^5^ cells per dish) and cultured overnight. Tech‐tetrahedra were added (500 µL per well,1 µM), and after 8 h of incubation, the medium was removed, washed with PBS once and 1 mL of fresh culture solution was added. Add 1 mL JC‐1 dyeing solution, incubate at 37 °C for 20 min, discard the dying solution and wash with JC‐1 buffer solution twice. After Hoechst staining, images were photographed and analyzed using CLSM.

#### 4.3.9 Bio‐TEM of MCF‐7 and MCF‐10A Cells

Cells were first treated by PBS, incomplete Tech‐tetrahedra, and Tech‐tetrahedra for 8 h, respectively. Cells were washed three times with PBS, and then centrifuged at 1200 × g for 3 min. After removing the supernatant, 2 mL 2.5% glutaraldehyde was added slowly to infiltrate cells, followed by 12 h storage at 4°C. The cells were further fixed with 1 mL 1% osmium tetroxide. The samples were then dehydrated with ethyl alcohol, and infiltrated by pure resin. The infiltrated samples were placed in a mold and polymerized at 60°C for 24 h, and then cut into 70 nm ultrathin sections using an ultramicrotome. The ultrathin sections were placed on copper grids and stained with uranyl acetate (2% in ethanol) and lead citrate. TEM images of MCF‐7 cells were observed using a transmission electron microscope.

#### 4.3.10 Measurement of the ROS Generation and Ca^2+^

MCF‐7 and MCF‐10A cells were seeded in a 6‐well plate at a density of 5×10^4^ cells per well and cultured for 12 h, respectively. Then, 500 µL of Tech‐tetrahedra, incomplete Tech‐tetrahedra, and PBS were added to the medium at the same concentration of 1 µM and incubated for 8 h. After washing with PBS for three times, the cells were stained with DCFH‐DA, Rhod‐2, AM or JC‐10 probe and detected by using Confocal Laser Scanning Microscopy.

#### 4.3.11 Detection of Intracellular Adenosine Triphosphate (ATP)

MCF‐7 and MCF‐10A cells were seeded in a 6‐well plate at a density of 5 × 10^5^ cells per well in 1 mL of DMEM and cultured for 12 h. Then, 500 µL of Tech‐tetrahedra, incomplete Tech‐tetrahedra, and PBS were added to the medium at the same concentration of 1 µM and incubated for 8 h. After washing with PBS for three times, 200 µL of lysis buffer were added to each well and mixed thoroughly. The supernatant was collected after centrifugation at 12 000 × g for 5 min at 4°C. The working solution was prepared by adding 100 microliters of ATP reagent. The relative light unit (RLU) values were measured in chemiluminescence mode. After testing the standard samples, linear regression equations were calculated to determine the ATP levels.

#### 4.3.12 Cellular Seahorse Energy Metabolism Assay

Analysis of MCF‐7 and MCF‐10A cells were plated in plates and 500 µL 1 µM of Tech‐tetrahedra, incomplete Tech‐tetrahedra and PBS were added to the medium and incubated for 8 h, respectively. Then, the excess medium was removed. The OCR and ECAR were measured, and measurements were performed according to the manufacturer's protocol.

#### 4.3.13 Cell Apoptosis Assay

The cells were plated in six‐well plates (5 × 10^5^ cells per well) and incubated overnight. After treatments (500 µL, 1 µM of Tech‐tetrahedra, incomplete Tech‐tetrahedra and PBS) for 8 h, the cells were incubated with 5 µL of annexin‐V and 1 µL of propidium iodide at room temperature away from light for 15 min. Then the stained cells were analyzed with FCM.

#### 4.3.14 Cell Proliferation Assay

Cell proliferation assay was performed using a Yefluor 488 EdU flow cytometry assay kit (Yeasen, 40278ES60). MCF‐7 and MCF‐10A cells were seeded into a 6‐well plate (5 × 10^5^ cells per well) and incubated with different samples at 37°C, 5% CO2 for 24 h. After treatments (500 µL, 1 µM of Tech‐tetrahedra, incomplete Tech‐tetrahedra, and PBS) for 5 h, cells were incubated with EdU for 3 h, fixed with 4% paraformaldehyde for 15 min, and permeabilized with 0.5% Triton X‐100 for 20 min. Cells were incubated with the click reaction mixture at room temperature in dark for 30 min. Images were obtained by using CLSM.

#### 4.3.15 In Vitro Cell Scratch Healing Assay

For the in vitro cell scratch healing assay, cells were seeded into a 6‐well plate and cultured for 12 h. Afterward straight lines were scratched across the surface of the well with a p200 pipette tip. The fluorescence was used to image the initial scratch. Then the well was gently washed twice with medium to remove the detached cells and replenished with fresh serum‐free medium. And then Tech‐tetrahedra, incomplete Tech‐tetrahedra and PBS were added to the well with a concentration of 1 nM, respectively. The images of scratch were collected after incubation with varied durations, and detected by using a fluorescence microscope. The areas of scratch were measured with ImageJ software.

#### 4.3.16 Transwell Migration Assays

MCF‐7 and MCF‐10A cells (5 × 10^4^) were seeded into the upper chamber of 6‐well transwell inserts and cultured at 37°C for 12 h. Then cells were incubated with 500 µL, 1 µM Tech‐tetrahedra, incomplete Tech‐tetrahedra, and PBS in 500 µL DEME medium without FBS for 48 h respectively. DEMD medium supplemented with 10% (v/v) FBS was added into the lower chamber. After fixation and staining, images were taken with an inverted microscope.

#### 4.3.17 Transwell Invasion Assays

Matrigel was diluted 1:4 in PBS at 4°C (on ice). Added 50–60 µL matrigel to the upper chamber surface of 6‐well transwell inserts, and incubated in the incubator at 37°C for 1 h to polymerize the matrigel into a gel film. MCF‐7 and MCF‐10A cells (5 × 10^4^) were seeded into the upper chamber with matrigel and cultured at 37°C for 12 h. Then cells were incubated with 500 µL, 1 µM Tech‐tetrahedra, incomplete Tech‐tetrahedra, and PBS in 500 µL DEME medium without FBS for 48 h, respectively. DEMD medium supplemented with 10% (v/v) FBS was added into the lower chamber. After fixation and staining, images were taken with an inverted microscope.

#### 4.3.18 Observation of Actin Cytoskeleton

MCF‐7 and MCF‐10A were cultured in confocal dishes and incubated for 12 h. After treatments (500 µL, 1 µM of Tech‐tetrahedra, incomplete Tech‐tetrahedra, and PBS) for 8 h, cells were permeabilized with 0.2% Triton X‐100 and blocked with 5% BSA. Cells were further incubated with anti‐cortactin antibody overnight, and incubated with Cy3‐labeled secondary antibody for 1 h. Then, cells were washed by PBS for three times and incubated with FTIC‐labeled phalloidin and DAPI. The images were collected by CLSM.

#### 4.3.19 Transcriptome Sequencing and Data Analysis

Total RNA was extracted from treated and control cells using the SV Total RNA Isolation System according to the manufacturer's instructions, and the quality and concentration of extracted RNA were assessed. Only RNA samples with A260/A280 ratios between 1.8 and 2.2, A260/A230 ratios >2.0, and RNA integrity number (RIN) values >7.0 were used for subsequent sequencing. The qualified RNA samples were then sent to Novogene Biotech Co., Ltd. (Beijing, China) for transcriptome sequencing. Gene Ontology (GO) and Kyoto Encyclopedia of Genes and Genomes (KEGG) were used to identify biological processes, cellular components, and molecular functions associated with DEGs. Protein functional annotation and classification were performed using the UniProt databases to obtain detailed protein information and functional domains.

#### 4.3.20 Western Blot

Cells was cultured in six‐well plates (≈5 × 10^5^ cells per well) for overnight. Next day, the cells were washed and incubated with Tech‐tetrahedron (500 µL, 1 mM, per well), Incomplete Tech‐tetrahedron, PBS for 24 h, respectively. After digestion, the cells were collected and the protein concentration was determined according to the steps of the BCA protein concentration test kit. The protein was then isolated using 10% SDS‐PAGE and transferred to a PVDF membrane, and then blocked with 5% BSA at room temperature for 2 h. After that, the membrane was washed with TBST for 5 min with three times. And incubated with anti‐polycystin‐1 and anti‐β‐actin antibody overnight at 4°C. Wash the membrane again with TBST for 5 min with three times and incubated with the second antibody labeled with horseradish peroxidase (HRP) for 2 h. Wash membrane with TBST for 10 min with 3 times again and reacted with chemiluminescence detection reagent for 2 min. Proteins were detected employing the chemiluminescent system.

#### 4.3.21 Establishment of Mice Model Bearing Breast Cancer Cell Lines and Animal Experiments

Fifteen 4‐week‐old female nude mice were randomly divided into three groups and inoculated with MCF‐7 breast cancer cells (4 × 10^6^ cells) after 1 week of environment adaptation. Then the tumor volume was monitored and measured. The tumor volume was calculated as: A × B^2^/2 (A was the long axis and B was the short axis). When the tumor volume grew to ≈50 mm^3^, 200 µL of the Tech‐tetrahedron (1 mM) using an unlabeled hairpin was intratumorally injected to the mice for diagnostic imaging. 3D live epi‐fluorescence imaging was performed at 0, 2, 4, 8, and 16 h after injection. For anti‐tumor evaluation, 200 µL of the Tech‐tetrahedron was intratumorally injected every 3 days, and the tumor volume and weight of mice were recorded. After 2 more weeks, the mice were sacrificed. Their blood was collected for blood routine and blood biochemistry tests. And tumors were excised, weighed, and photographed, followed by soaking in the 10% formalin for histological examinations by EGFR immunohistochemistry analysis, TUNEL immunohistochemistry analysis, Ki‐67 immunofluorescence analysis, and polycystin‐1 immunohistochemistry analysis. The positive cell rates of five indicators across the three treatment groups were determined by randomly selecting five fields of view under 20× magnification. Additionally, spleens were harvested from all groups, homogenized to obtain single‐cell suspensions, and subjected to flow cytometric analysis using fluorescently conjugated antibodies against CD3.CD4, and CD8 to quantify T lymphocyte subsets, including their percentages and absolute counts, for assessing systemic immune responses.

#### 4.3.22 Statistical Analyses

Statistical analyses were conducted via GraphPad Prism (version 10). All data were presented as mean ± standard deviation (SD). Two groups comparison was performed using independent‐samples tests, and multiple groups comparison was conducted with one‐way analysis of variance with post hoc multiple comparisons (least significant difference, LSD). The level of statistical significance was indicated on the graphs by asterisks (*, **, or ***) for *p*‐values of less than 0.05, 0.01, and 0.001, respectively.

## Author Contributions

Conceptualization: K. Chang, M. Chen, Y. Pi. Methodology: R. Deng, J. Sheng, B. Niu. Visualization: W. Fu, Y. Yang, Z. Xie. Funding acquisition: K. Chang, M. Chen. Project administration: K. Chang, M. Chen, Y. Pi. Writing – original draft: S. Xie, Y. He, M. Gong, J. Wang.

## Funding

National Science and Technology Major Project of China (2025ZD0551102). National Natural Science Foundation of China [82122042, 82030066, 82372352, U23A20477]. Chongqing Science Fund for Distinguished Young Scholars (CSTB2022NSCQ‐JQX0007).

## Conflicts of Interest

The authors declare that they have no conflicts of interest.

## Supporting information




**Supporting File**: advs76061‐sup‐0001‐SuppMat.docx.

## Data Availability

All data are available in the main text or the Supplementary Materials.
