# Supplementary material for: Designer Dynamic DNA Nanoaggregate in Living Cell for Mitochondrial Energy Restriction
Source: Adv Sci (Weinh). 2026 Jun 9:e76061. Online ahead of print. doi: 10.1002/advs.76061 (PMC13336737; doi:10.1002/advs.76061)
Supplement: Supplementary file 1 — Supporting File: advs76061‐sup‐0001‐SuppMat.docx. [file ADVS-9999-e76061-s001.docx]

**Supporting Information**

**Designer dynamic DNA nanoaggregate in living cell for mitochondrial energy restriction**

*Ruijia Deng^1†^, Jing Sheng^1†^, Ben Niu^1†^, Wenjuan Fu^1^, Yingjie Yang**^1^, Zuowei Xie^1^, Shuang Xie^1^, Yunxuan He^1^, Meilin Gong^1^, Jue Wang^1^, Yan Pi^2^*, Ming Chen^1^*, Kai Chang^1,3^**

**Contents**

Figure S1. Chain-exclusion experiment of Tech-tetrahedron

Figure S2. Physicochemical characterization of the step-wise assembly of the DNA tetrahedron by dynamic light scattering (DLS)

Figure S3. Stability of Tech-tetrahedron

Figure S4**.** Stability of DNA hairpins.

Figure S5. Time-dependent mitochondrial colocalization of transfected DNA hairpins in living cells.

Figure S6**.** Biological effects of DNA hairpins.

Figure S7. Quantification of telomerase reverse transcriptase (TERT) expression in three breast-derived cell lines

Figure S8. Standard calibration curve for TE quantification by fluorescence ELISA

Figure S9. The fluorescence intensity measured in the ELISA assay for TE levels

Figure S10. The fluorescence of CHA when H1b, H2b and P6 react alone

Figure S11. Optimization of the tetrahedral hairpin connector sequences

Figure S12. Optimization of the molar ratio between hairpins and tetrahedral during the conjugation process

Figure S13. Optimization of hairpin stem length

Figure S14**.** Time-dependent intracellular trafficking and lysosomal dissociation behavior of Tech-tetrahedron.

Figure S15**.** Effect of mitochondrial inhibition on intracellular mitochondrial localization of Tech-tetrahedron.

Figure S16**.** Role of TPP modification and mitochondrial membrane potential in Tech-tetrahedron localization.

Figure S17. Confocal microscopy images of MCF-7 cells following 4-h incubation with FAM-labeled Tech-tetrahedron, Incomplete-Tech (Without P6), or PBS control

Figure S18. Distinct distribution patterns of Tech-tetrahedra

Figure S19. Transmission electron microscopy (TEM) images of mitochondria in MCF-7 cells following different treatments

Figure S20. Intracellular reactive oxygen species (ROS) generation visualized in MCF-7 cells

Figure S21. Cytosolic Ca²⁺ levels measured in MCF-7 cells

Figure S22. Intracellular reactive oxygen species (ROS) generation visualized in MCF-10 cells

Figure S23. Cytosolic Ca²⁺ levels measured in MCF-10 cells

Figure S24. Standard curve for ATP quantification

Figure S25. Basal respiration and ATP production of MCF-7 (OCR)

Figure S26. Glycolysis and glycolytic reserve of MCF-7 (ECAR)

Figure S27. Basal Respiration and ATP Production of MCF-10A (OCR)

Figure S28. Glycolysis and Glycolytic Reserve of MCF-10A (ECAR)

Figure S29. Mitochondrial localization and biological effect of mut-CHA

Figure S30. OCR kinetic traces under different substrates

Figure S31. Quantification of maximal respiration, ATP-linked OCR, and ADP-linked OCR

Figure S32. Cell scratch healing assay of MCF-7

Figure S33. Cell scratch healing assay of MCF-10A

Figure S34. Transwell migration assays of MCF-7

Figure S35. Transwell migration assays of MCF-10A

Figure S36. Transwell invasion assays of MCF-7

Figure S37. Transwell invasion assays of MCF-10A

Figure S38. Confocal immunofluorescence of F-actin and cortactin in MCF-7 cells

Figure S39. Confocal immunofluorescence of F-actin and cortactin in MCF-10A cells

Figure S40. Time-dependent body weights alteration of tumor-bearing mice

Figure S41. Blood routine analysis of mice with different treatments

Figure S42. Serum Biochemical analysis of mice with different treatments

Figure S43. Time-dependent epi-ﬂuorescence monitoring results in living mice with diﬀerent treatments

Figure S44. Representative gating strategy for splenic T lymphocytes collected

Figure S45. Quantification of splenic T cell populations

Figure S46. Representative immunohistochemical micrographs of EGFR membrane staining in day-18 MCF-7 tumors

Figure S47. Representative immunohistochemical micrographs of Ki-67

Figure S48. Representative immunohistochemical micrographs of AR

Figure S49. Representative immunohistochemical micrographs of PC-1

Figure S50. Representative immunohistochemical micrographs of TUNEL

Table S1. Sequence of oligonucleotides used in Tech-tetrahedron

Table S2. Sequence of oligonucleotides used in linear double-hairpin DNA construct.

Table S3. Sequence of oligonucleotides used in mut-CHA.

Table S4. Differentially expressed genes identified as primary factors

Table S5. Differentially expressed genes identified as confounding factors

Table S6. Genes specifically responsive to the formation of complete aggregates


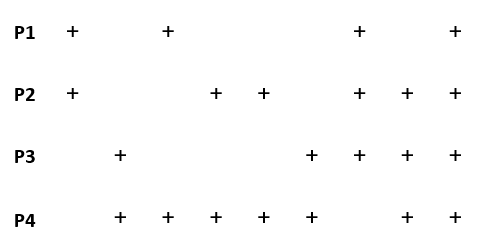

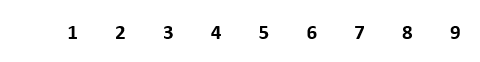

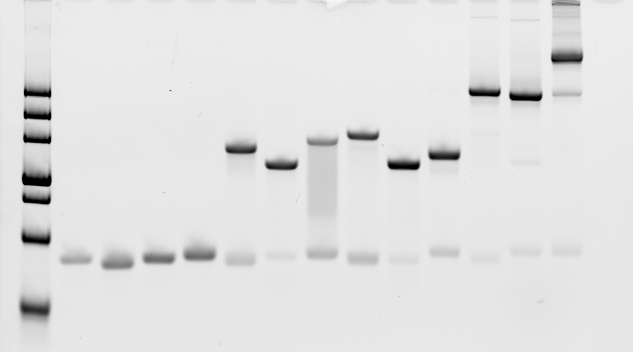


**1**

**2**

**3**

**4**

**5**

**6**

**7**

**8**

**9**

**S1**

**S2**

**S3**

**S4**

**Figure S1.** Chain-exclusion experiment of Tech-tetrahedron. Lane 1: P1+P2; Lane 2: P3+P4; Lane 3: P1+P4; Lane 5: P2+P4; Lane 6: P1+P3+P4; Lane 7: P1+P2+P3; Lane 8: P2+P3+P4; Lane 9: P1+P2+P3+P4. A tetrahedral structure exhibiting the lowest mobility and the highest molecular weight when all four chains participate in the reaction.


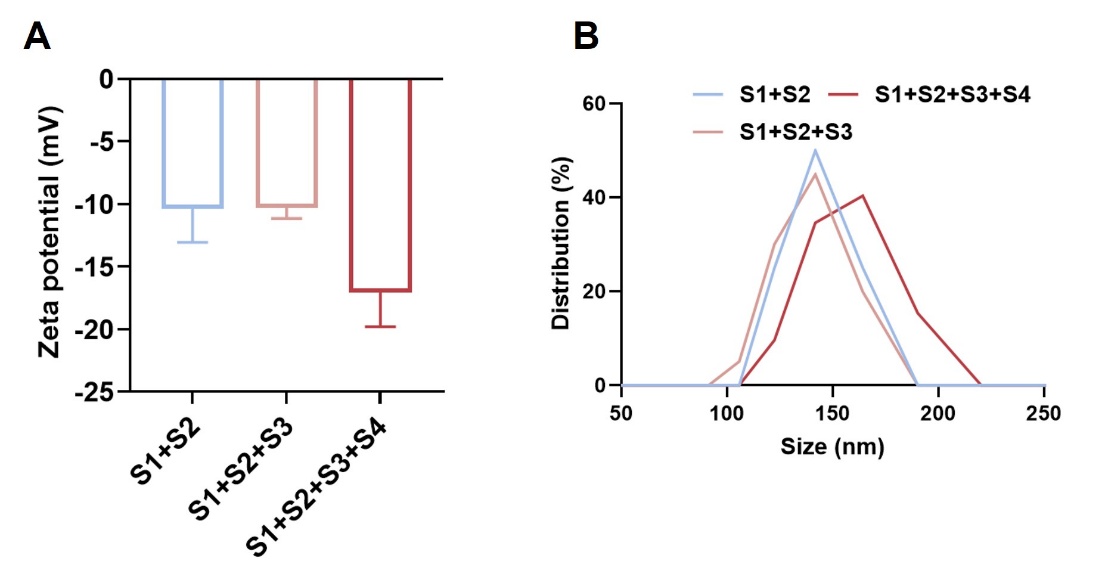


**Figure S2.** Physicochemical characterization of the step-wise assembly of the DNA tetrahedron by dynamic light scattering (DLS). A) Zeta potential of partially (S1 + S2; S1 + S2 + S3) and fully (S1 + S2 + S3 + S4) assembled structures. Progressive addition of the four oligonucleotide strands (P1–P4) shifts the surface charge to increasingly negative values, confirming successful hybridization and compaction. Data are shown as mean ± SD (n = 3). B) Corresponding volume-weighted hydrodynamic size distributions. The average diameter broadens and shifts toward larger sizes upon completion of the four-strand assembly, consistent with formation of the tetrahedral nanostructure.


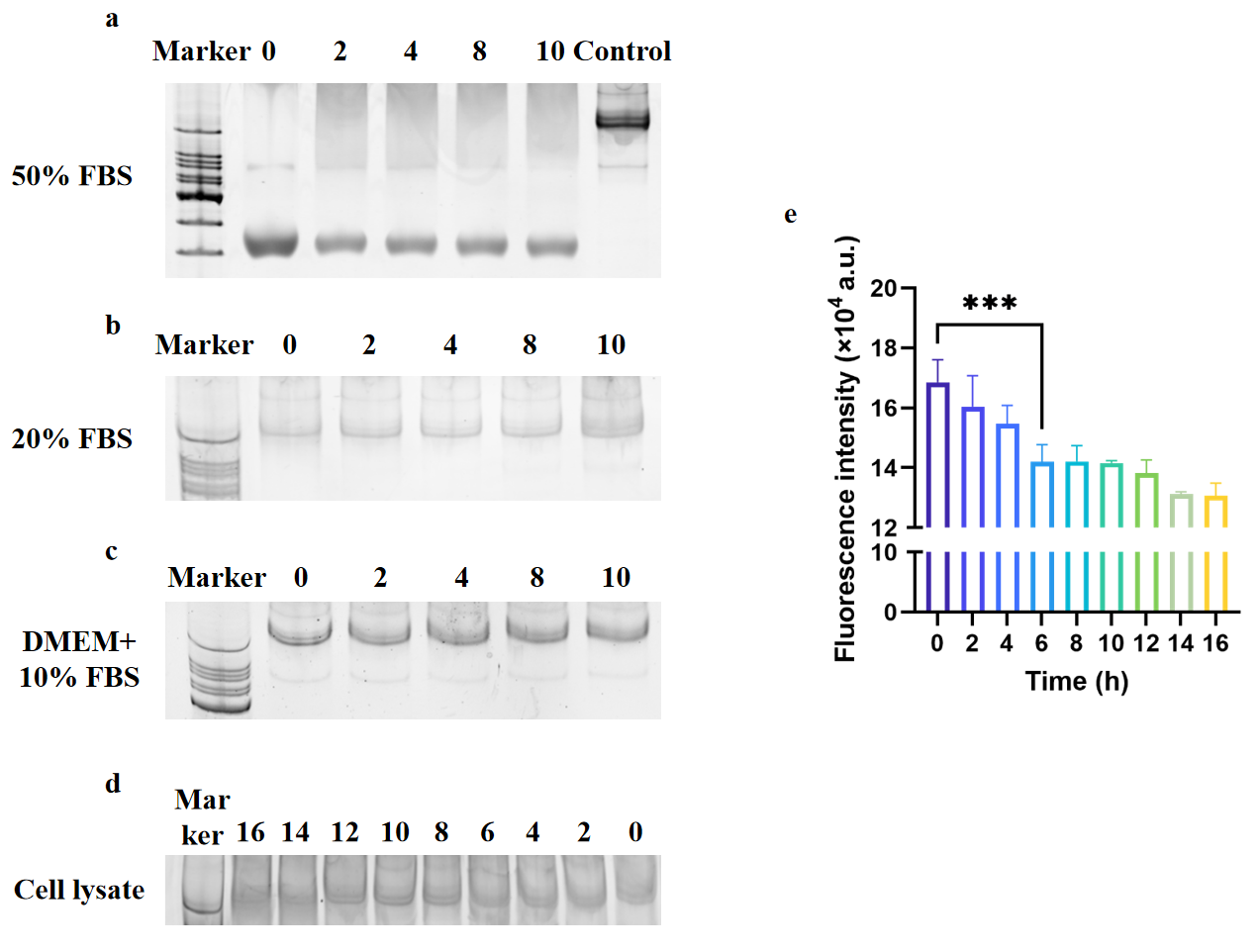


**500 bp**

**500 bp**

**500 bp**

**50 bp**

**Figure S3.** Stability of Tech-tetrahedron

(a) Native PAGE analysis of the Tech-tetrahedron after incubation in 50% FBS at 37 °C for the indicated time points.

(b) Native PAGE analysis of the Tech-tetrahedron after incubation in 20% FBS at 37 °C for the indicated time points.

(c) Native PAGE analysis of the Tech-tetrahedron after incubation in DMEM containing 10% FBS at 37 °C for the indicated time points.

(d) Native PAGE analysis of the Tech-tetrahedron after incubation in cell lysate at 37 °C for the indicated time points (0–16 h).

(e) Fluorescence response of the Tech-tetrahedron following cell lysate pretreatment for the indicated time period. Data are presented as mean ± SD. Statistical significance is indicated in the panel.


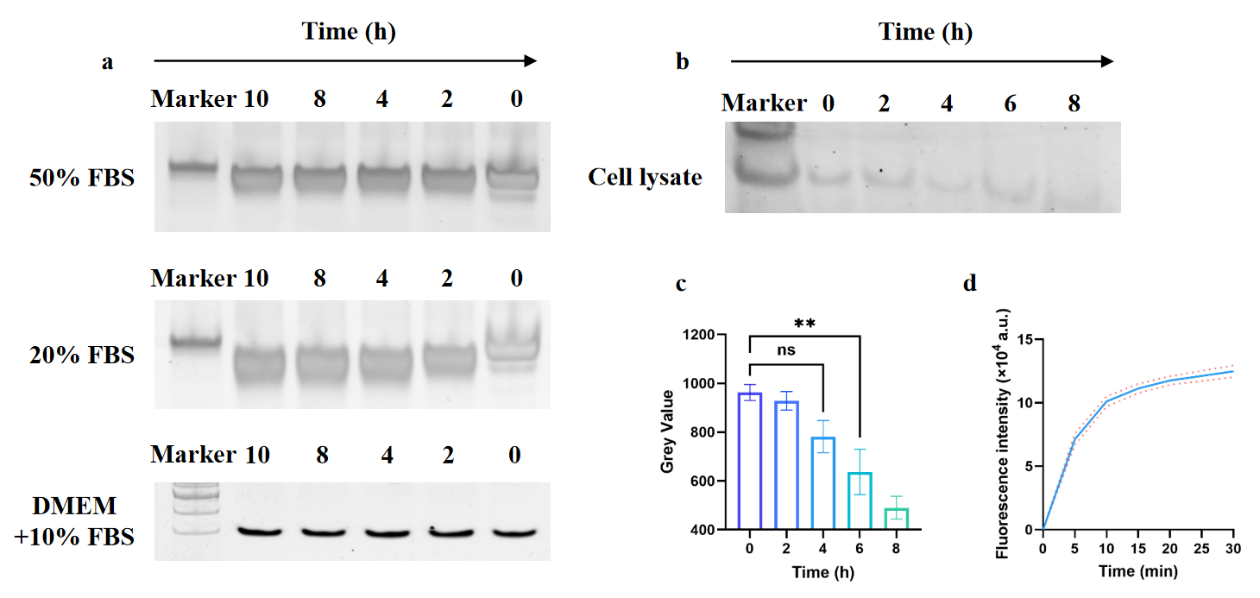


**40 bp**

**20 bp**

**20 bp**

**20 bp**

**Figure S4.** Stability of DNA hairpins.

(a) Native PAGE analysis of the structural stability of DNA hairpins under different extracellularly relevant conditions. A 50 bp DNA marker was used as the size standard.

(b) Native PAGE analysis of the stability of DNA hairpins in cell lysate.

(c) Quantification of PAGE band intensity for DNA hairpins incubated in cell lysate. Data are presented as mean ± SD. Statistical significance was determined as indicated.

(d) Fluorescence-based assessment of retained CHA activation capability of DNA hairpins before cellular application.


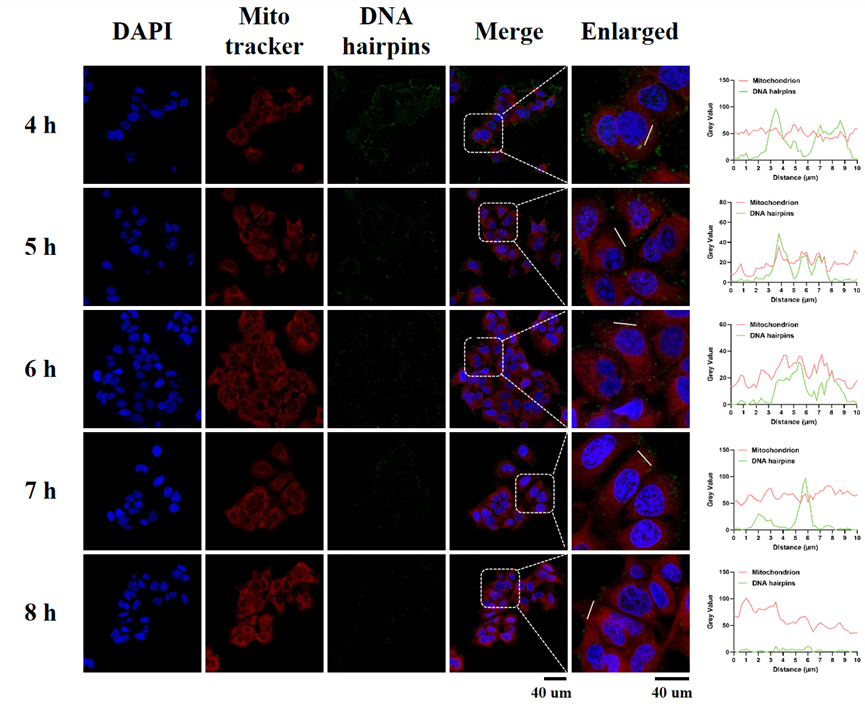


**Figure S5.** Time-dependent mitochondrial colocalization of transfected DNA hairpins in living cells. DNA hairpins (green) and MitoTracker (red) were incubate at 4, 5, 6, 7, and 8 h after lipofection. Nuclei were stained with DAPI (blue). Enlarged views and corresponding fluorescence intensity line profiles are shown on the right. Scale bars: 40 μm.

**
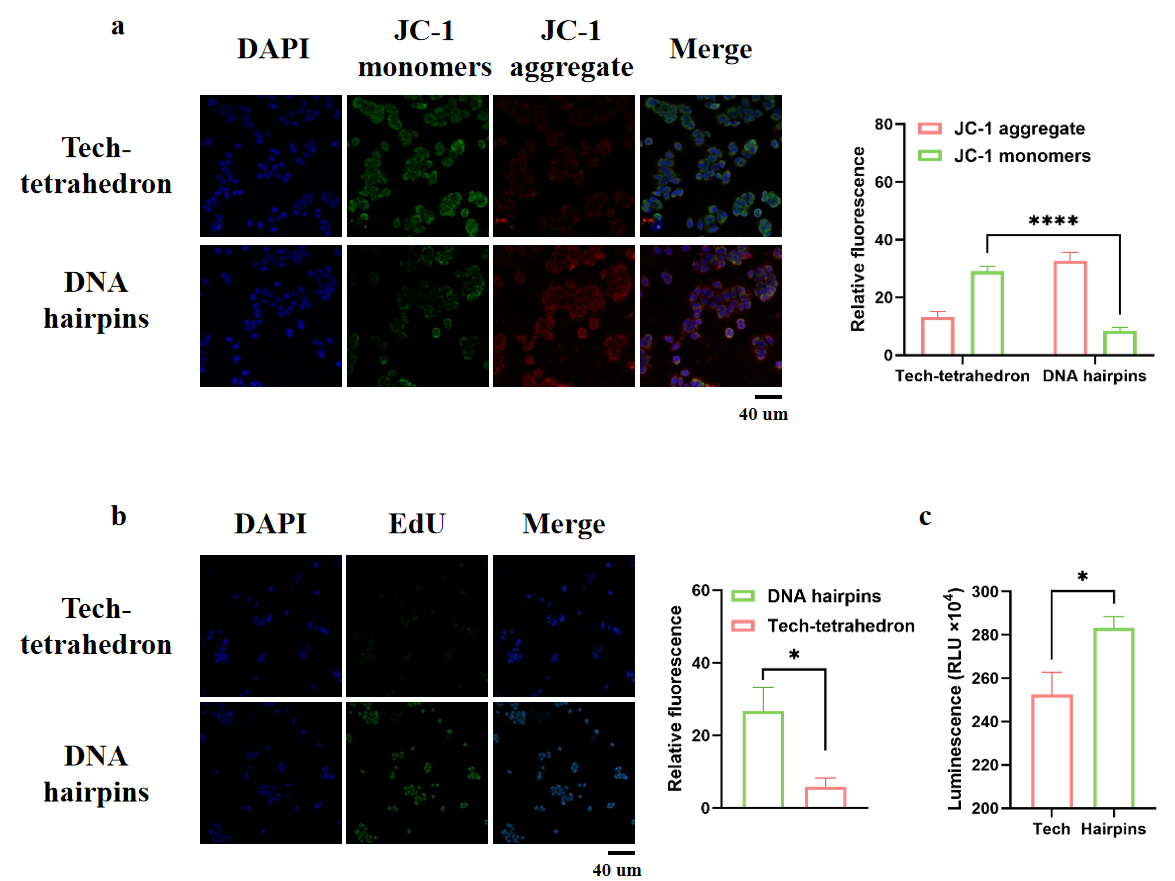
**

**Figure S6.** Biological effects of DNA hairpins.

(a) Assessment of mitochondrial membrane potential after treatment with Tech-tetrahedron or DNA hairpins. Nuclei were stained with DAPI (blue). The bar graph shows the quantitative analysis of JC-1 fluorescence signals. Scale bar: 40 μm.

(b) Evaluation of cell proliferation by EdU staining after treatment with Tech-tetrahedron or DNA hairpins. The bar graph shows the quantitative analysis of EdU fluorescence intensity. Scale bar: 40 μm.

(c) Intracellular ATP levels after treatment with Tech-tetrahedron or DNA hairpins. ATP production in cells treated with Tech-tetrahedron or DNA hairpins, measured by luminescence assay. Data are presented as relative luminescence units (RLU). Values are shown as mean ± SD, and statistical significance is indicated.


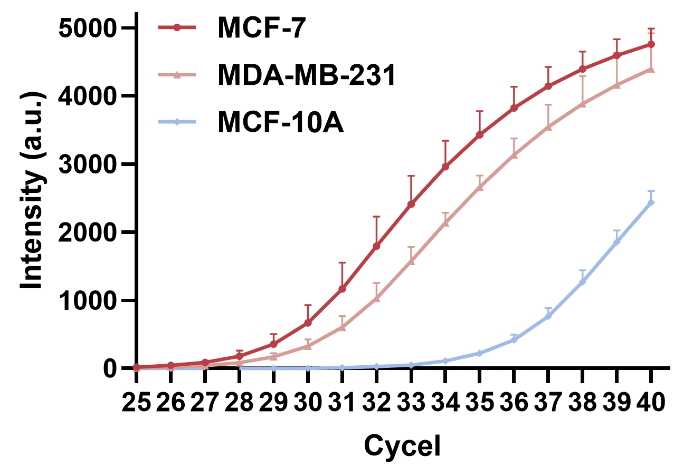


**Figure S7.** Quantification of telomerase reverse transcriptase (TERT) expression in three breast-derived cell lines. Real-time PCR amplification curves for TERT purified from MCF-7 (dark red), MDA-MB-231 (light red) and the non-tumorigenic MCF-10A (blue) cells. Fluorescence intensity was recorded after each cycle (25–40). Earlier onset of exponential amplification (lower Ct) and a steeper slope for MCF-7 indicate substantially higher TERT transcript abundance relative to the other two lines (4.75- and 11.94-fold versus MDA-MB-231 and MCF-10A, respectively). Data are shown as mean ± SD (n = 3).


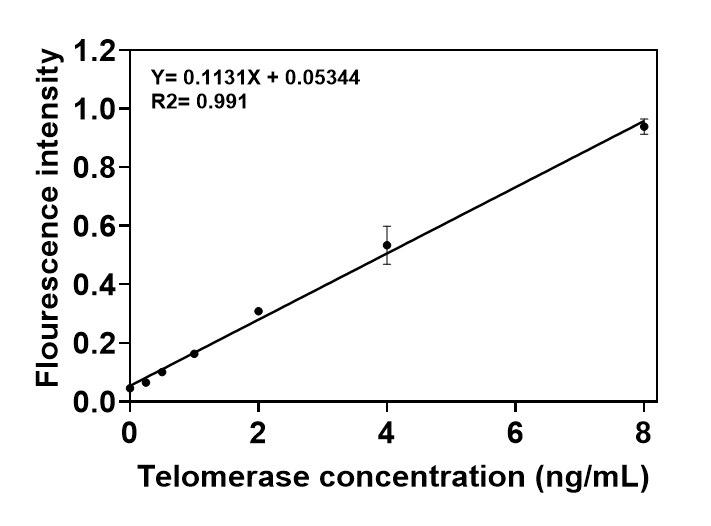


**Figure S8.** Standard calibration curve for TE quantification by fluorescence ELISA. Serial dilutions of TE (0–8 ng mL⁻¹) were analyzed under identical assay conditions. The resulting fluorescence intensity (mean ± SD, n = 3) increases linearly with protein concentration, yielding the regression equation Y = 0.1131 X + 0.0534 (R² = 0.991). This calibration was used to convert raw fluorescence signals from cell lysates into absolute TE concentrations.


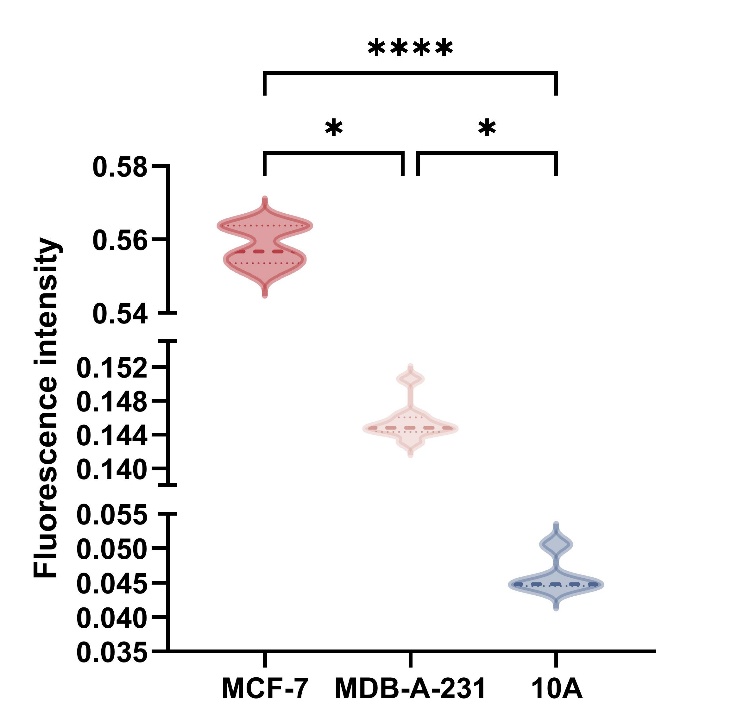


**Figure S9.** The fluorescence intensity measured in the ELISA assay for TE levels. Violin plots depict the distribution of fluorescence read-outs Calculated TE concentrations were 116.6 ± 4.3 ng µL⁻¹ for MCF-7, 69.8 ± 3.1 ng µL⁻¹ for MDA-MB-231, and 58.6 ± 2.7 ng µL⁻¹ for the non-tumorigenic MCF-10A cells.


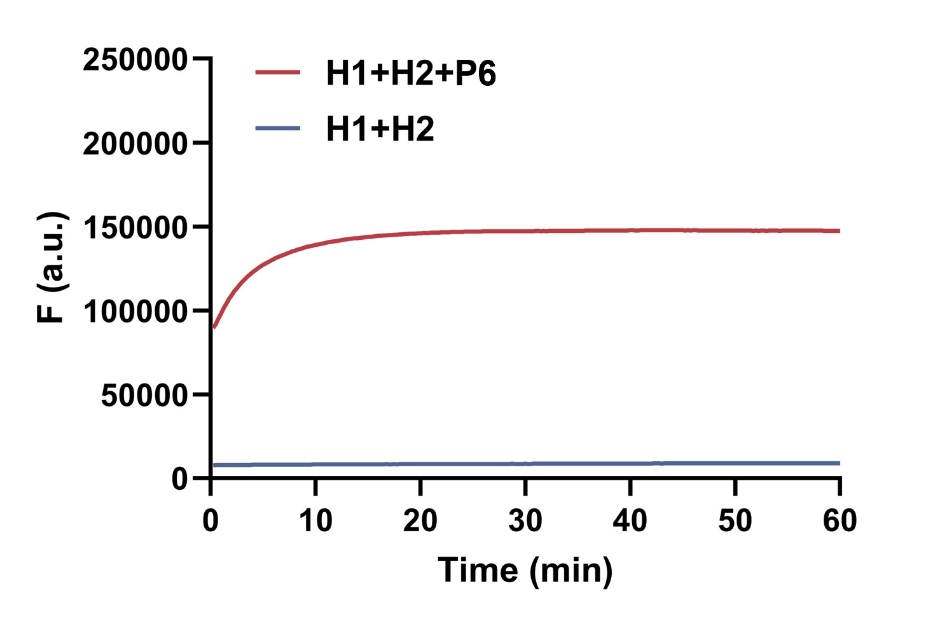


**Figure S10.** The fluorescence of CHA when H1b, H2b and P6 react alone.


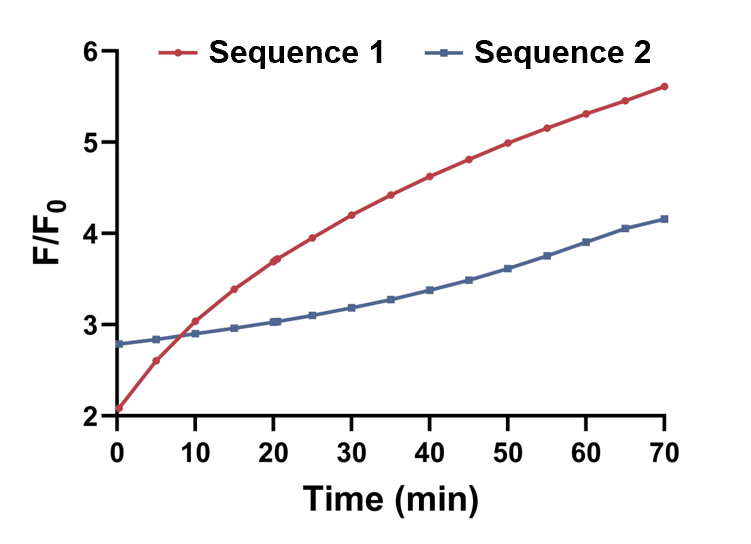


**Figure S11.** Optimization of the tetrahedral hairpin connector sequences. Sequence 1: The linker sequences between P1 and P2 are identical, with both composed of repeated thymine (T) bases; Sequence 2: The linker sequences between P1 and P2 are different, consisting of repeated thymine (T) bases and repeated adenine (A) bases respectively.


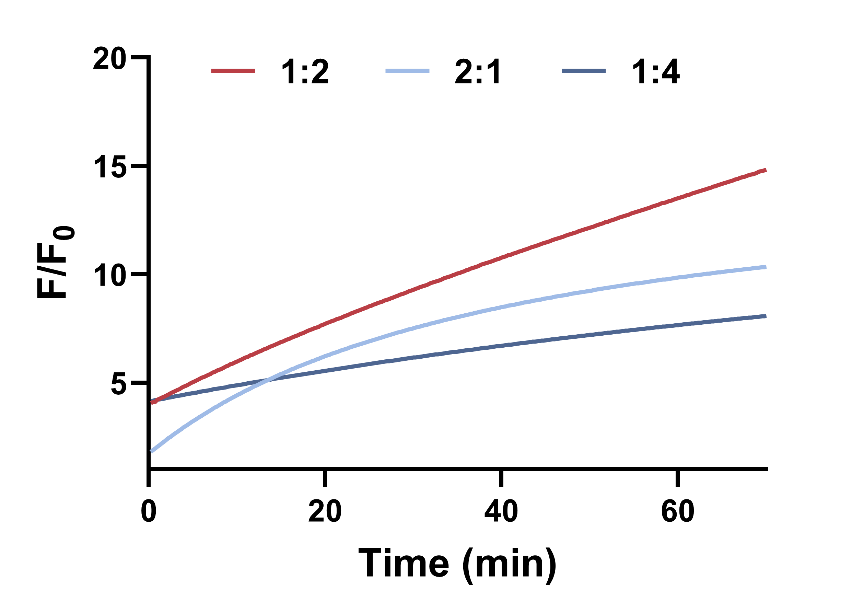


**Figure 12.** Optimization of the molar ratio between hairpins and tetrahedral during the conjugation process. The ratio is expressed as hairpin : tetrahedron (H1 or H2 : Tech-tetrahedron). Results demonstrated that efficient conjugation occurred at a tetrahedron-to-hairpin ratio of 1:2, yielding the highest fluorescence signal in the subsequent CHA reaction.

**
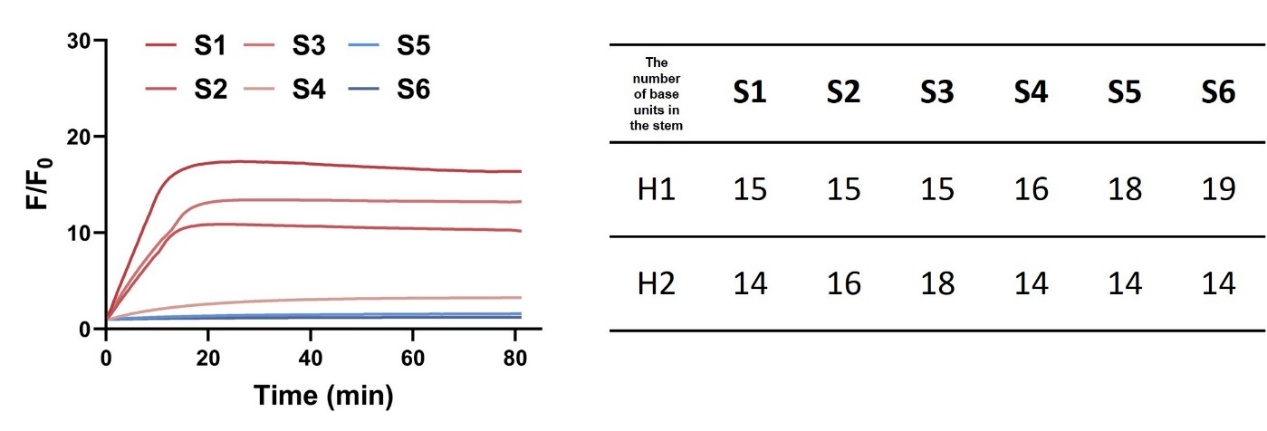
**

**Figure S13.** Optimization of hairpin stem length. Left: real-time fluorescence traces (F/F₀) collected at 37 °C for six hairpin pairs whose stem domains were systematically varied. Right: exact sequences and stem lengths. Tech-tetrahedra (50 nM) and hairpins were mixed at the previously optimized 2 : 1 molar ratio in reaction buffer and fluorescence was monitored for 80 min.


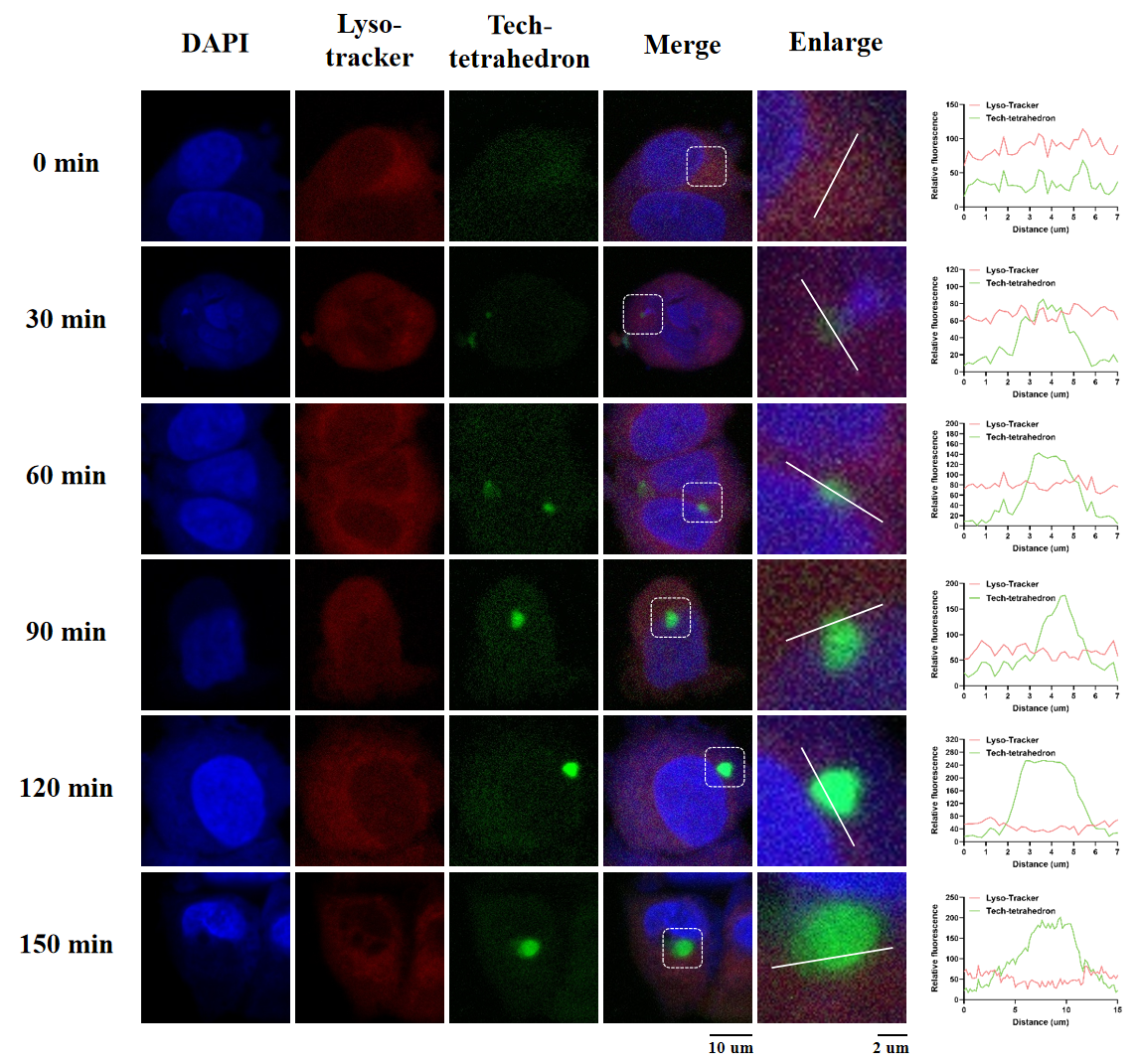


**Figure S14.** Time-dependent intracellular trafficking and lysosomal dissociation behavior of Tech-tetrahedron. Enlarged views and corresponding fluorescence intensity line profiles are shown on the right. Progressive reduction in colocalization with lysosomes was observed over time, suggesting dissociation from lysosome-associated compartments. Scale bars: 10 μm (main images) and 2 μm (enlarged views).


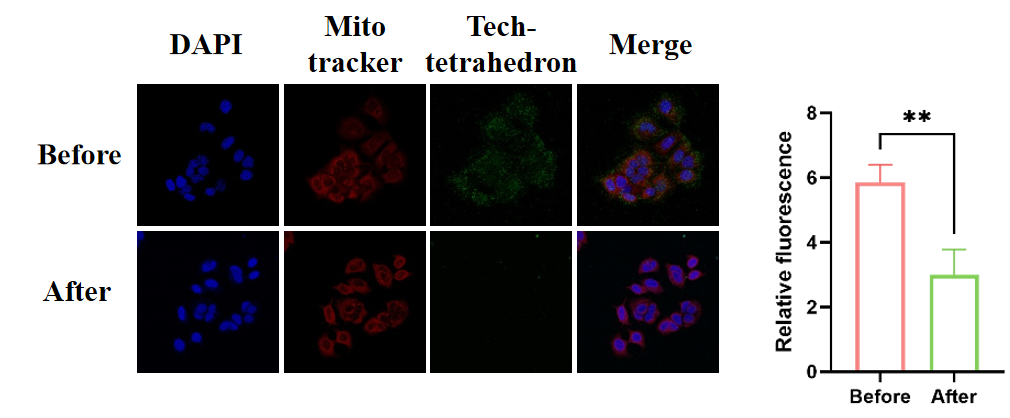


**Figure S15.** Effect of mitochondrial inhibition on intracellular mitochondrial localization of Tech-tetrahedron. The bar graph shows the quantitative analysis of mitochondrial colocalization-related fluorescence intensity. Scale bar: 40 μm.


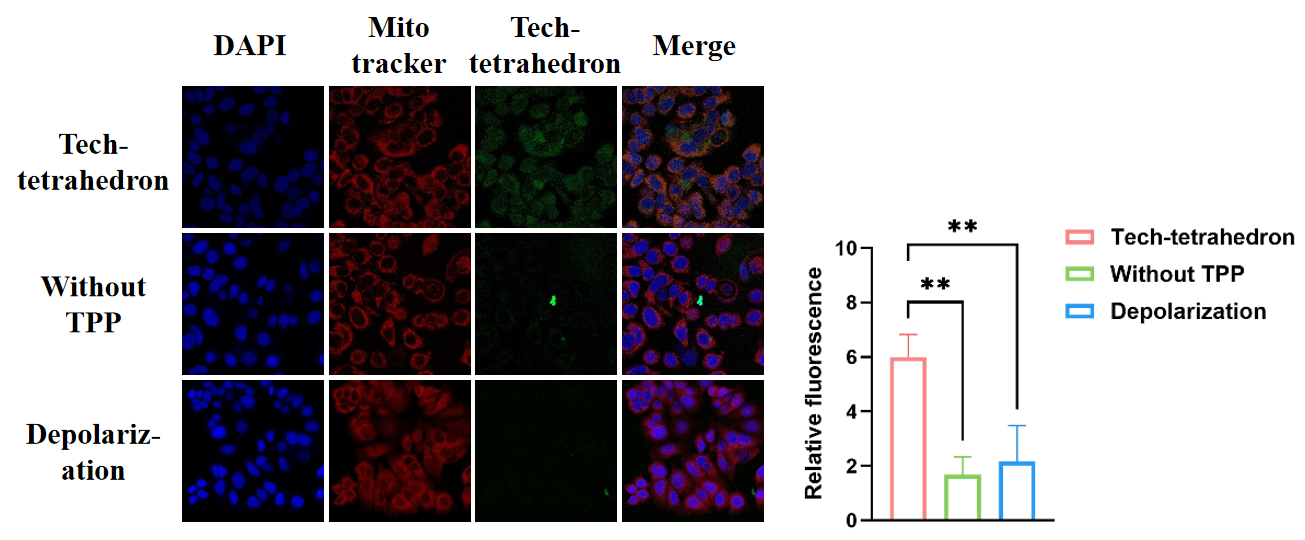


**Figure S16.** Role of TPP modification and mitochondrial membrane potential in Tech-tetrahedron localization. The bar graph shows the quantitative analysis of fluorescence intensity associated with mitochondrial localization. Scale bar: 40 μm.


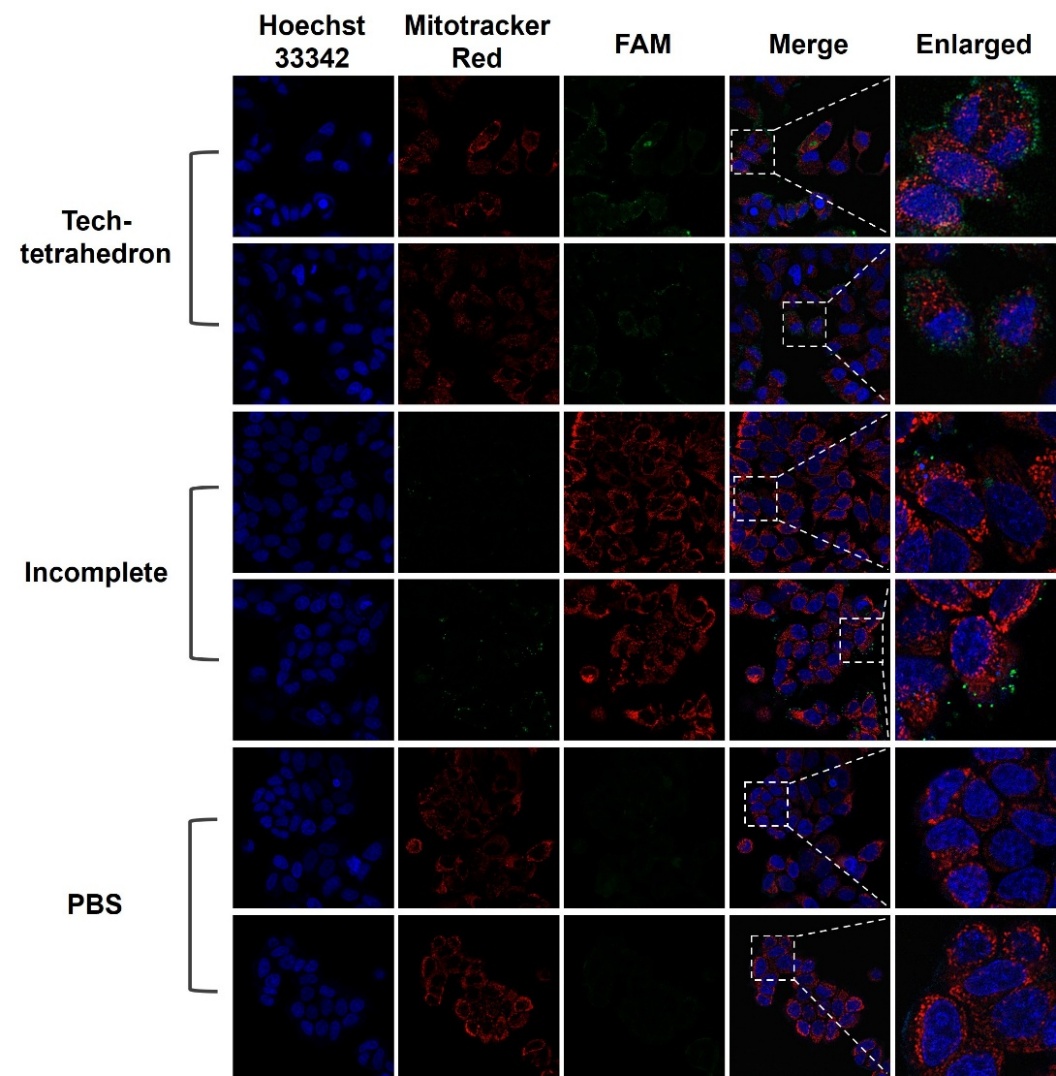


**Figure S17.** Confocal microscopy images of MCF-7 cells following 4-h incubation with FAM-labeled Tech-tetrahedron, Incomplete-Tech (Without P6), or PBS control. Blue: nuclei (Hoechst 33342); red: mitochondria (MitoTracker Red); green: tetrahedron (FAM).


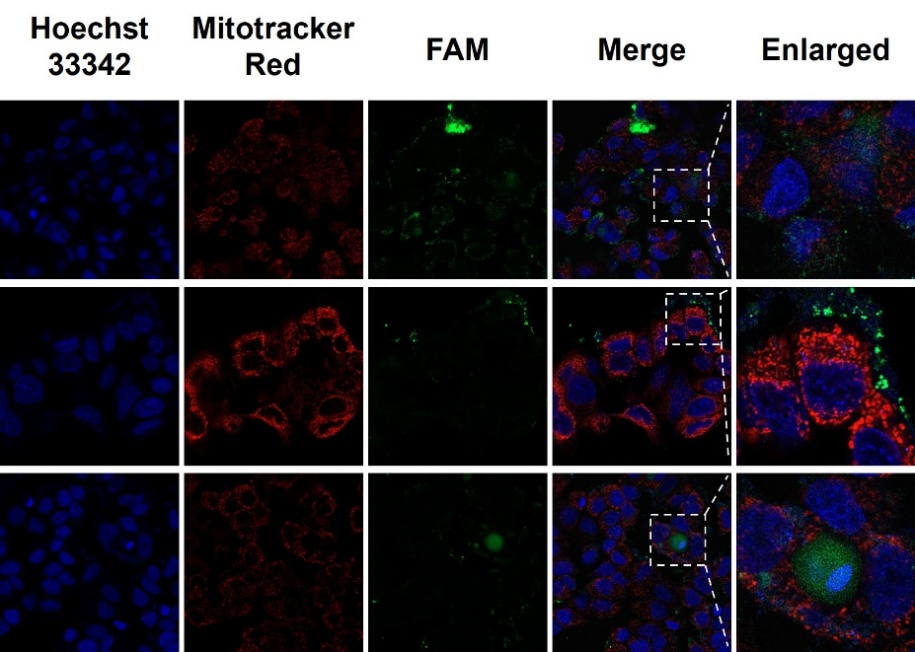


**Figure S18.** Distinct distribution patterns of Tech-tetrahedra. Confocal micrographs show the individual fluorescence channels, the merged image, and a magnified view of the region outlined by the dashed box. Green FAM signals reveal two characteristic localisation modes: dispersed puncta and larger aggregates.


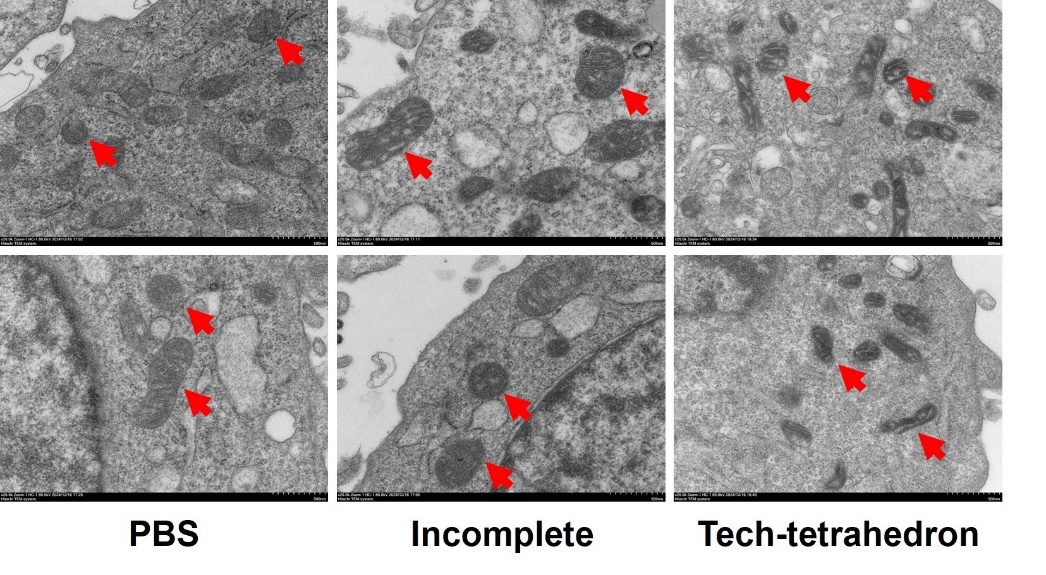


**Figure S19.** Transmission electron microscopy (TEM) images of mitochondria in MCF-7 cells following different treatments. Red arrows indicate representative mitochondria. Compared to the PBS and incomplete Tech-tetrahedron groups, Tech-tetrahedron treatment resulted in pronounced mitochondrial shrinking and morphological abnormalities.


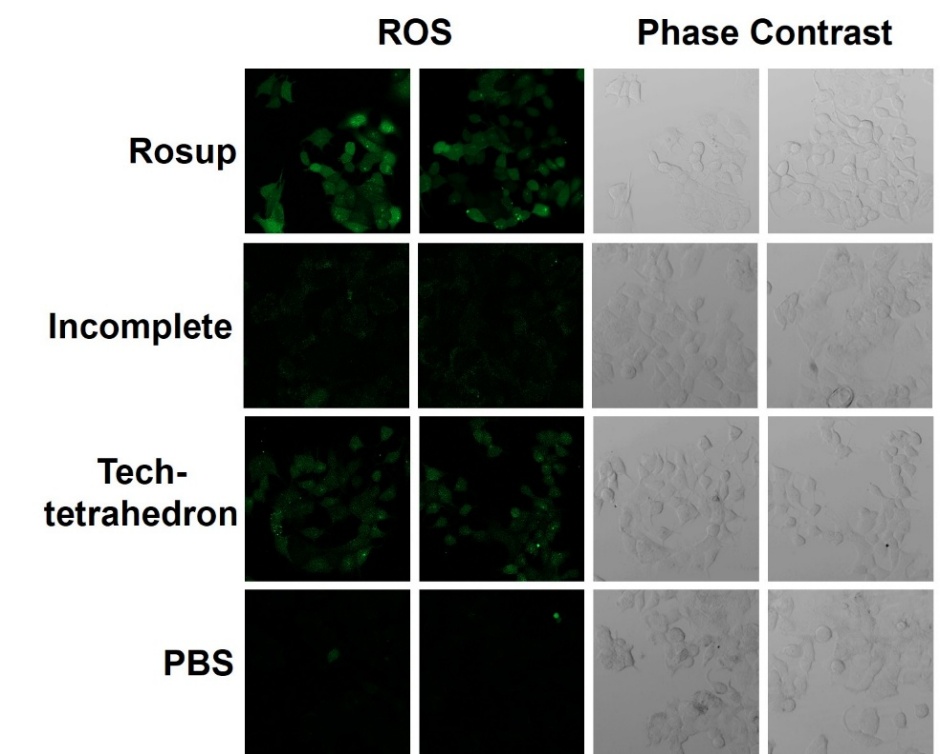


**Figure S20.** Intracellular reactive oxygen species (ROS) generation visualized in MCF-7 cells following 8-h treatment with ROS inducer Rosup (positive control), P6-deficient Incomplete-Tech, Tech-tetrahedron, or PBS control.


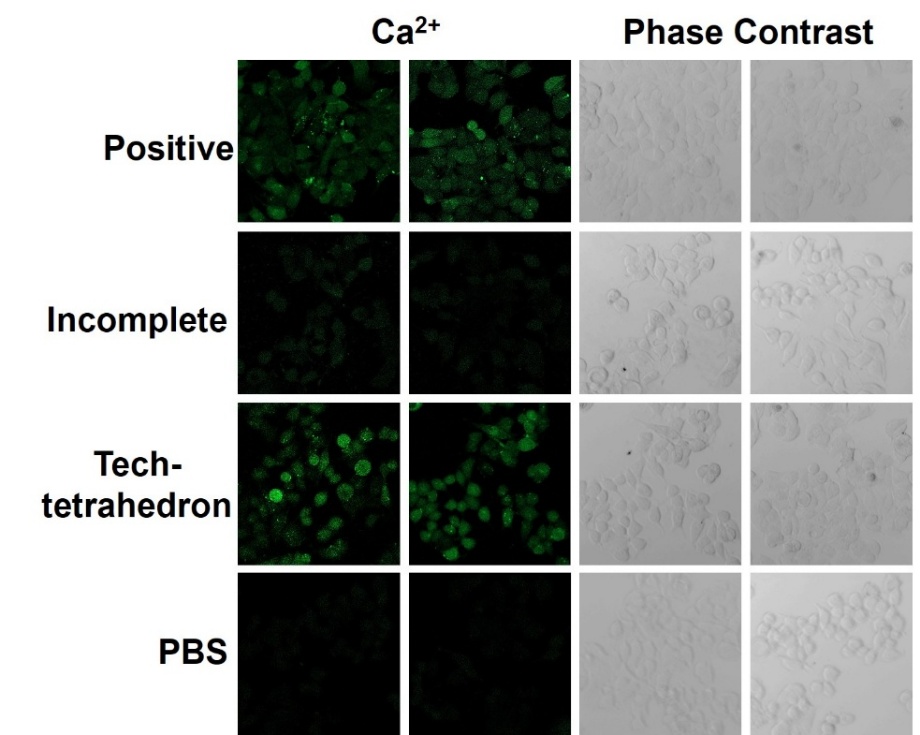


**Figure S21.** Cytosolic Ca²⁺ levels measured using Fluo-4 AM fluorescence (green) in MCF-7 cells under identical treatment with positive control, P6-deficient Incomplete-Tech, Tech-tetrahedron, or PBS control.


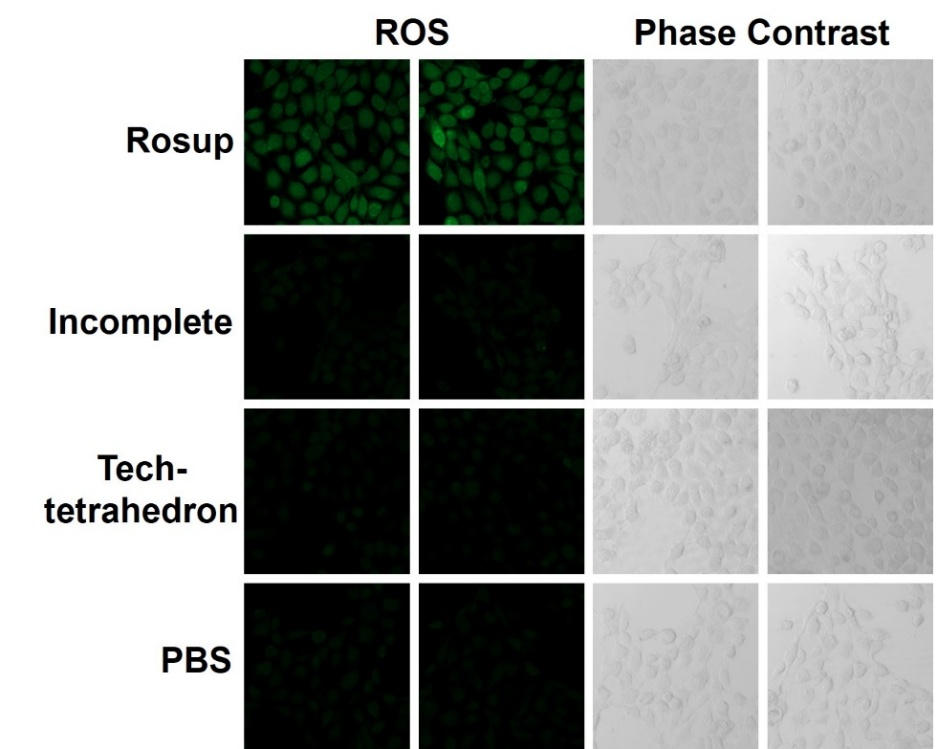


**Figure S22.** Intracellular reactive oxygen species (ROS) generation visualized in MCF-10 cells following 8-h treatment with ROS inducer Rosup (positive control), P6-deficient Incomplete-Tech, Tech-tetrahedron, or PBS control.


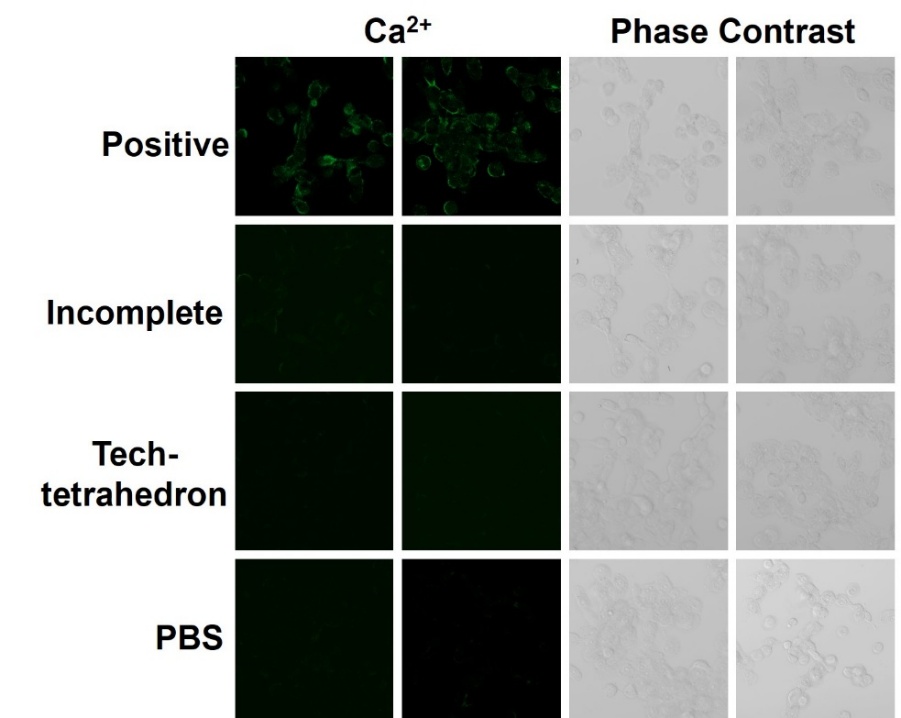


**Figure S23.** Cytosolic Ca²⁺ levels measured using Fluo-4 AM fluorescence (green) in MCF-10 cells under identical treatment with positive control, P6-deficient Incomplete-Tech, Tech-tetrahedron, or PBS control.


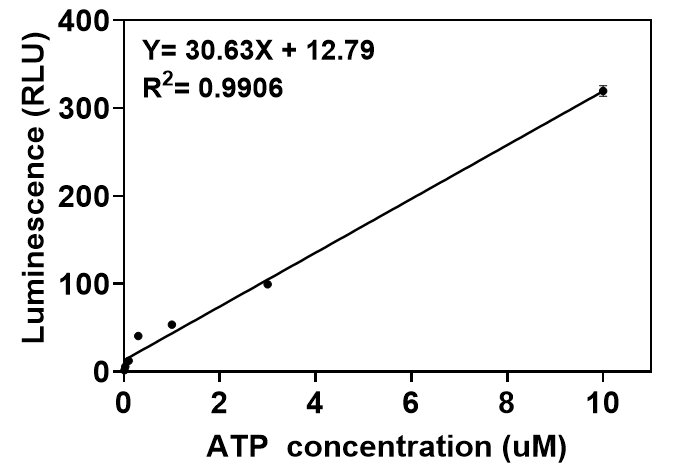


**Figure S24.** Standard curve for ATP quantification. ATP standard solutions at indicated concentrations (0–10 μM) were assessed, and luminescence intensity (RLU) was recorded. A strong linear correlation was observed between ATP concentration and luminescence signal (Y = 30.63X + 12.79, R² = 0.9906)


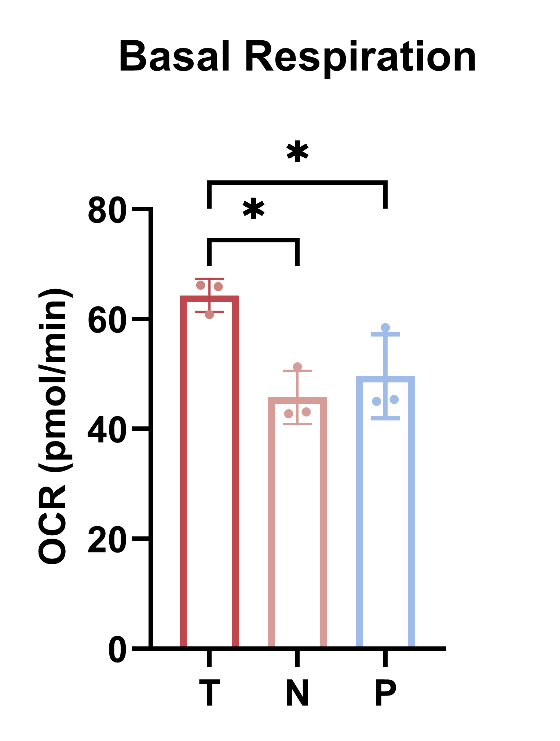


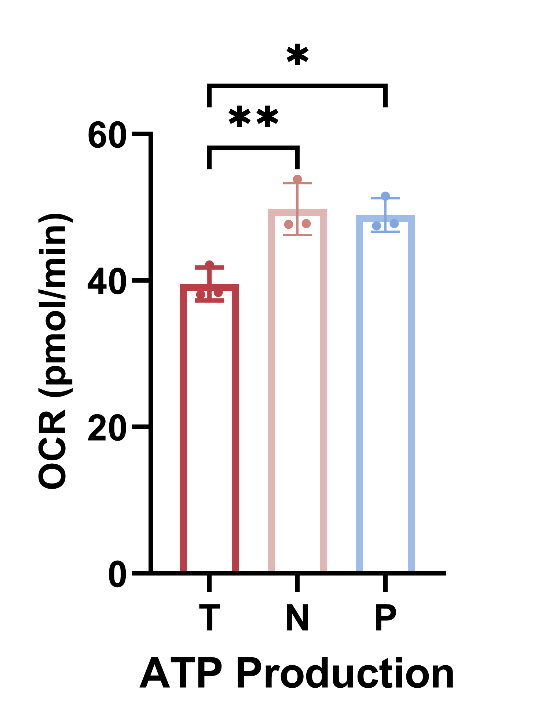

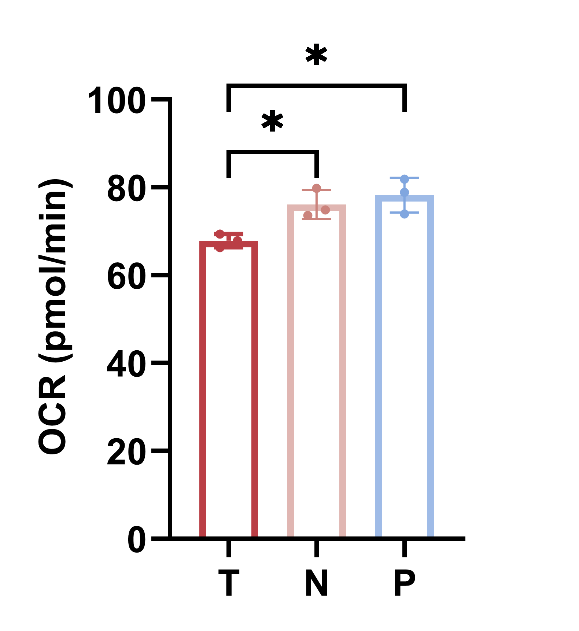


**Figure S25.** Basal respiration and ATP production of MCF-7 (OCR). T: Tech-tetrahedron; N: Incomplete; P: PBS.


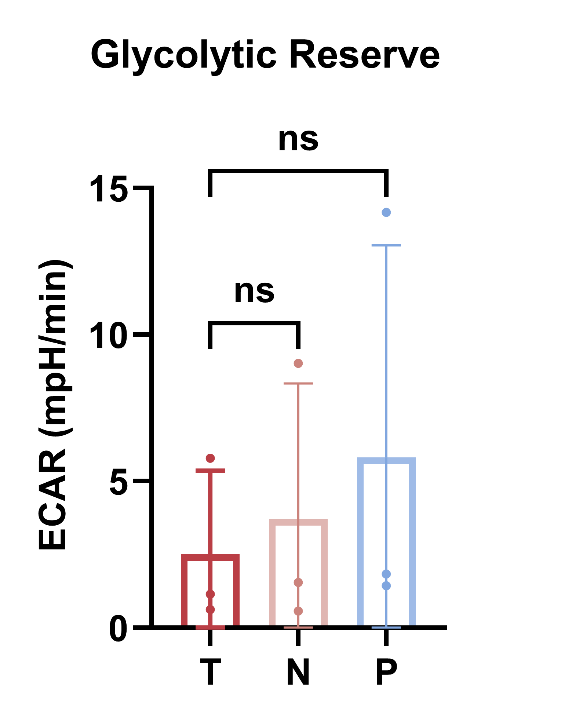

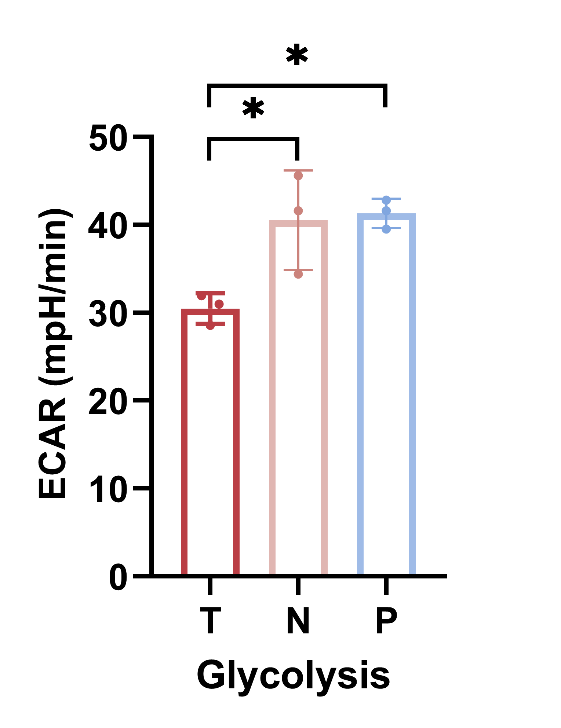


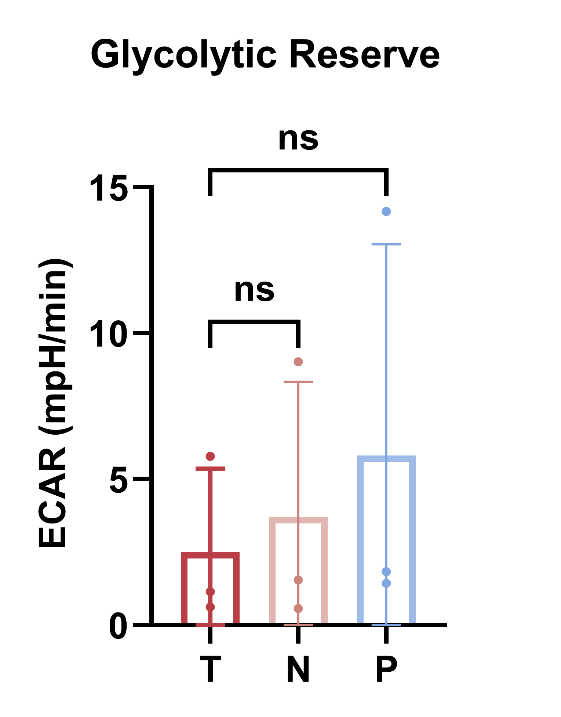


**Figure S26.** Glycolysis and glycolytic reserve of MCF-7 (ECAR). T: Tech-tetrahedron; N: Incomplete; P: PBS.


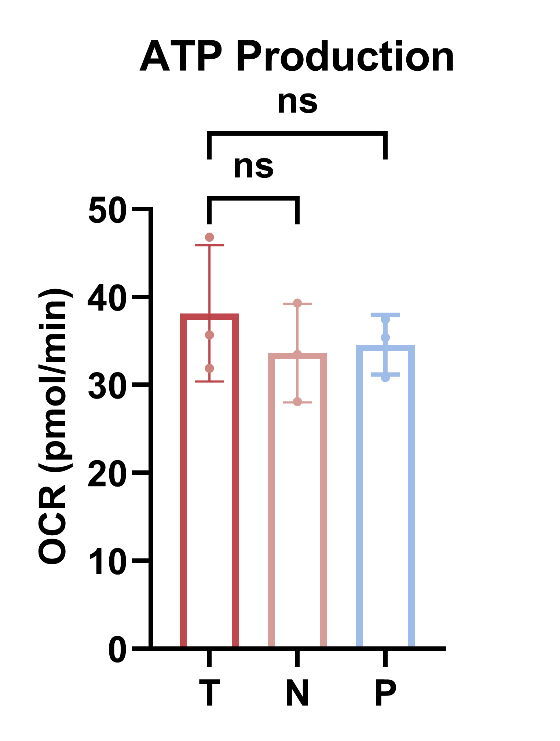

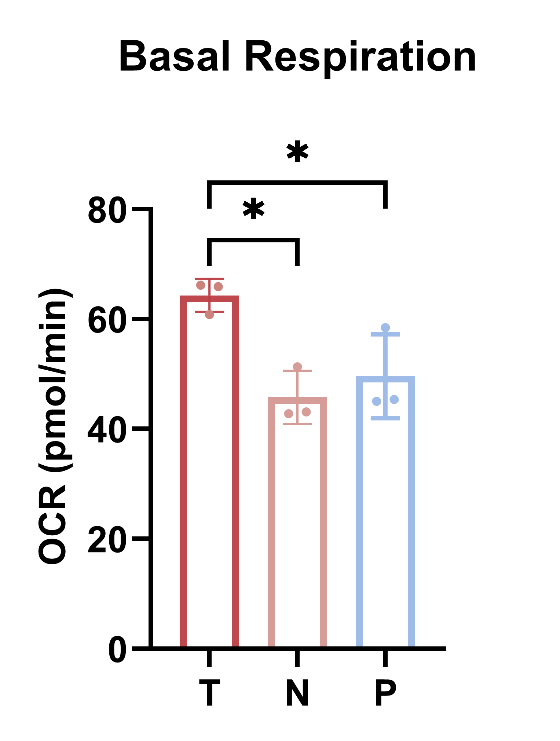


**Figure S27.** Basal Respiration and ATP Production of MCF-10A (OCR). T: Tech-tetrahedron; N: Incomplete; P: PBS.


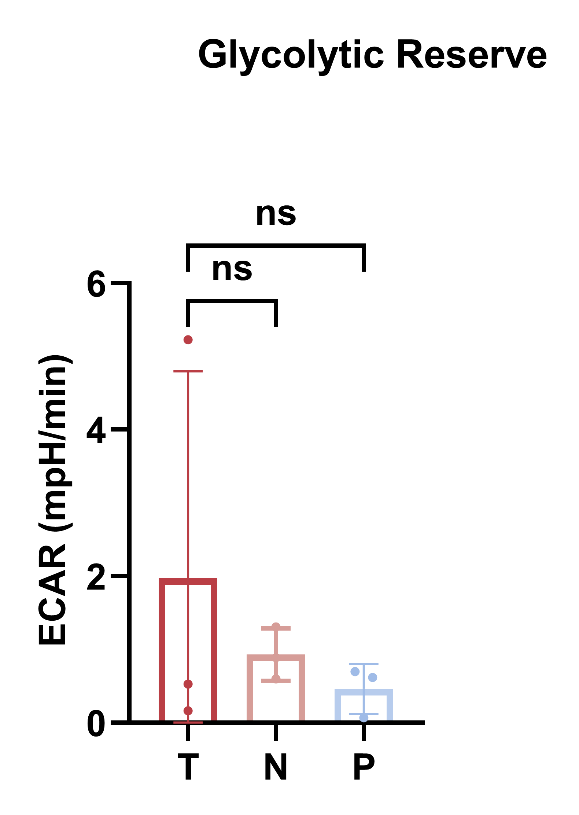


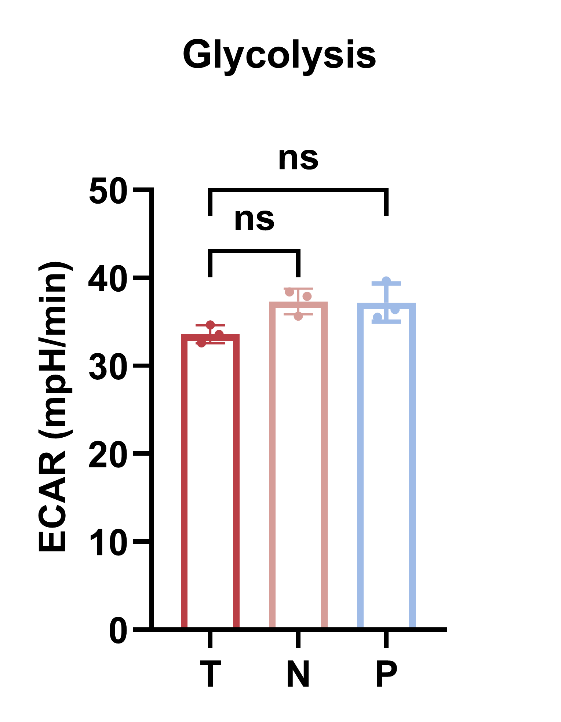

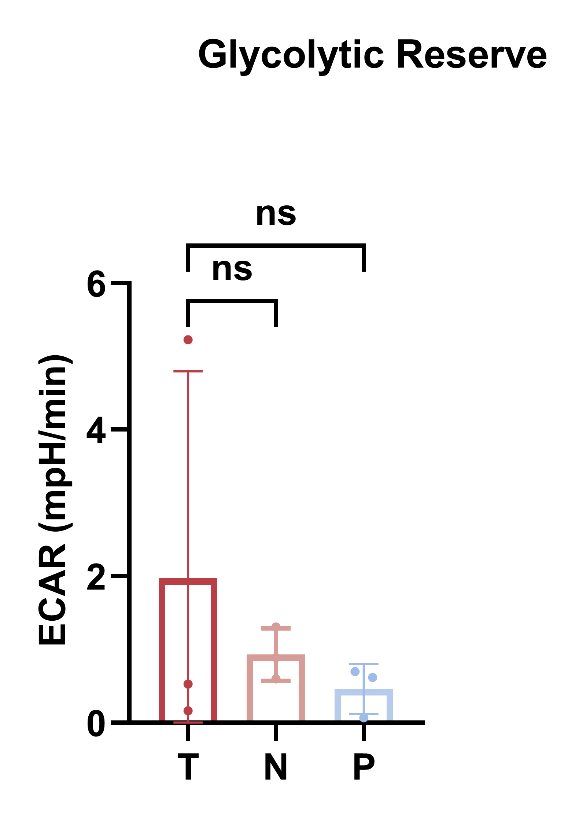


**Figure S28.** Glycolysis and Glycolytic Reserve of MCF-10A (ECAR). T: Tech-tetrahedron; N: Incomplete; P: PBS.


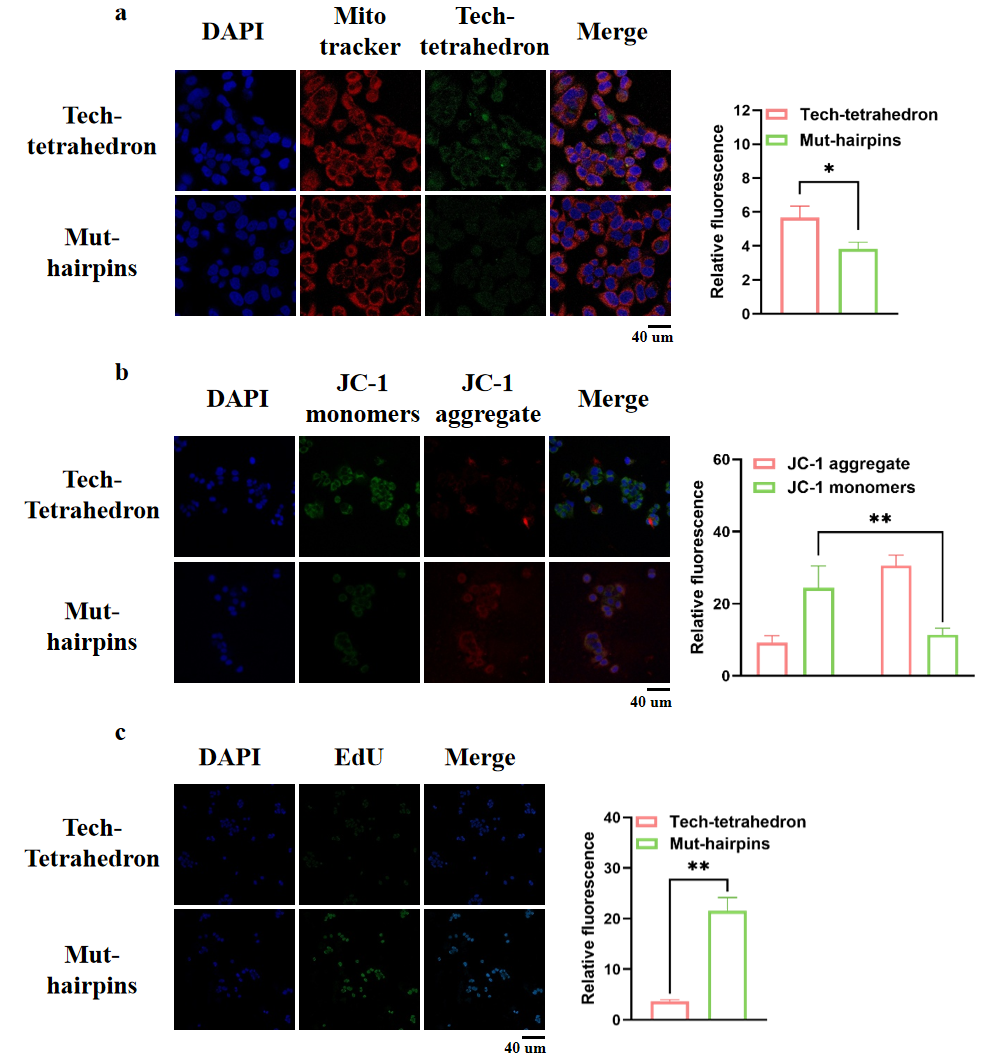


**Figure S29.** Mitochondrial localization and biological effect of mut-CHA.

(a) Effect of CHA-disabling hairpin mutation on mitochondrial localization behavior. The bar graph shows quantitative analysis of fluorescence intensity. Scale bar: 40 μm.

(b) Effect of CHA-disabling hairpin mutation on mitochondrial membrane potential. The bar graph shows the quantitative analysis of JC-1 fluorescence signals. Scale bar: 40 μm.

(c) Effect of CHA-disabling hairpin mutation on cell proliferation and mitochondrial function-related outcomes. The accompanying quantitative analysis compares the fluorescence signals between groups. Scale bar: 40 μm.

**
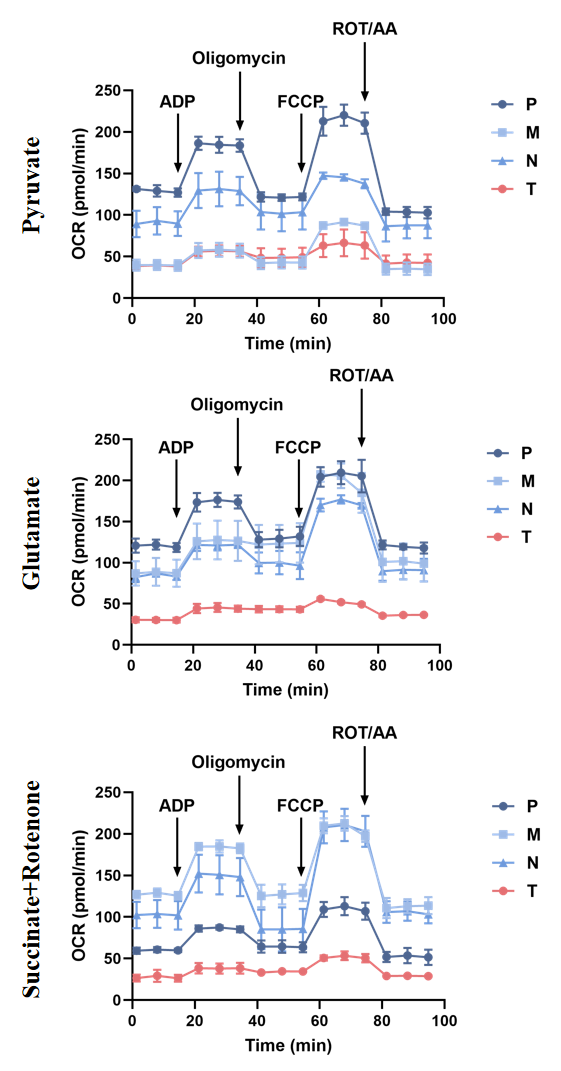
**

**Figure S30.** OCR kinetic traces under pyruvate-, succinate plus rotenone-, and glutamate-supported respiratory conditions in PBS (P), mutant hairpin control (2-mutCHA, M), incomplete system (N), and Tech-tetrahedron system (T). ADP, oligomycin, FCCP, and rotenone/antimycin A (ROT/AA) were sequentially injected as indicated.

**
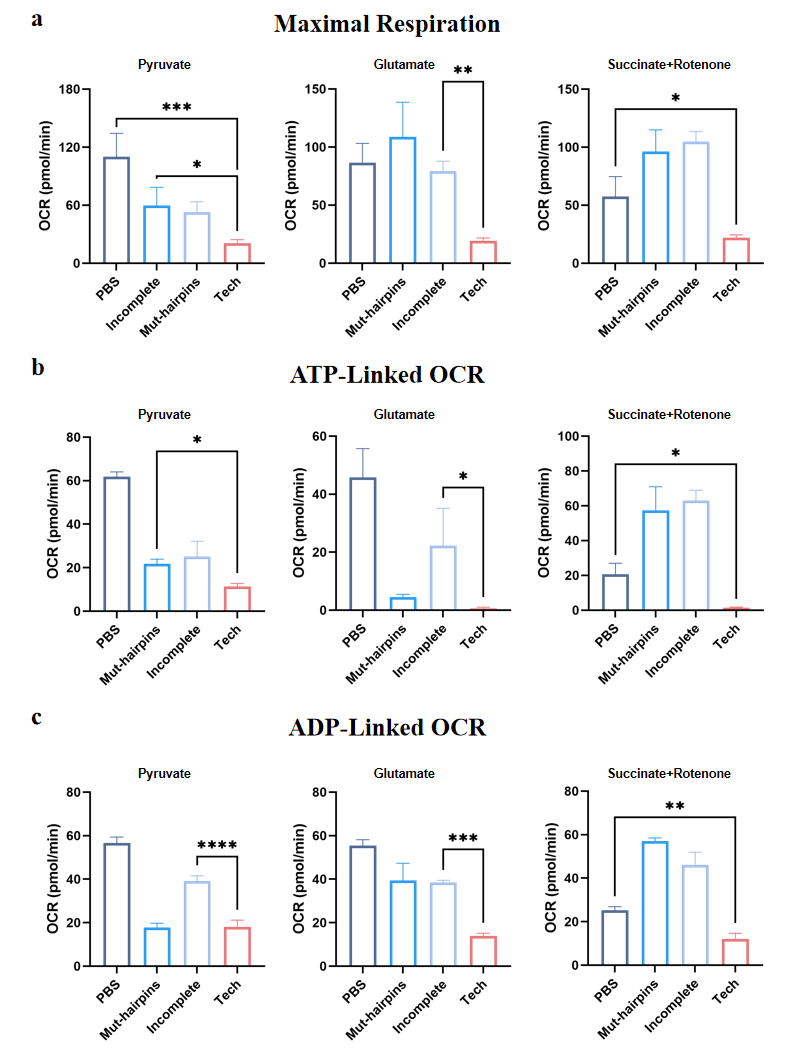
**

**Figure S31.** Quantification of maximal respiration (a), ATP-linked OCR (b), and ADP-linked OCR (c). The Tech-tetrahedron system showed the strongest suppression of mitochondrial respiration across all substrate conditions. Data are presented as mean ± SD. Statistical significance is indicated in the panels.


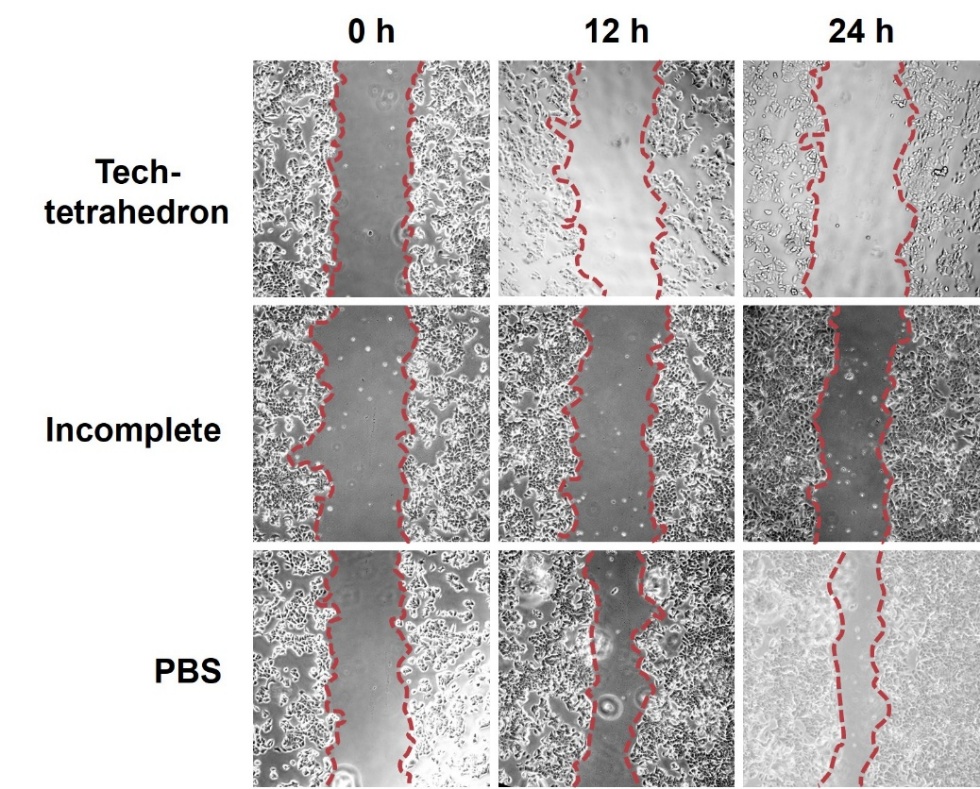


**Figure S32.** Cell scratch healing assay of MCF-7. MCF-7 cells were treated with Tech-tetrahedron, Incomplete Tech-tetrahedron, PBS, respectively. Cell migration was monitored at 0 h, 12 h, 24 h, respectively. For each gap, twelve measurement points are randomly selected to determine the width, and the mean value is calculated as the average width of the gap at the given time point.


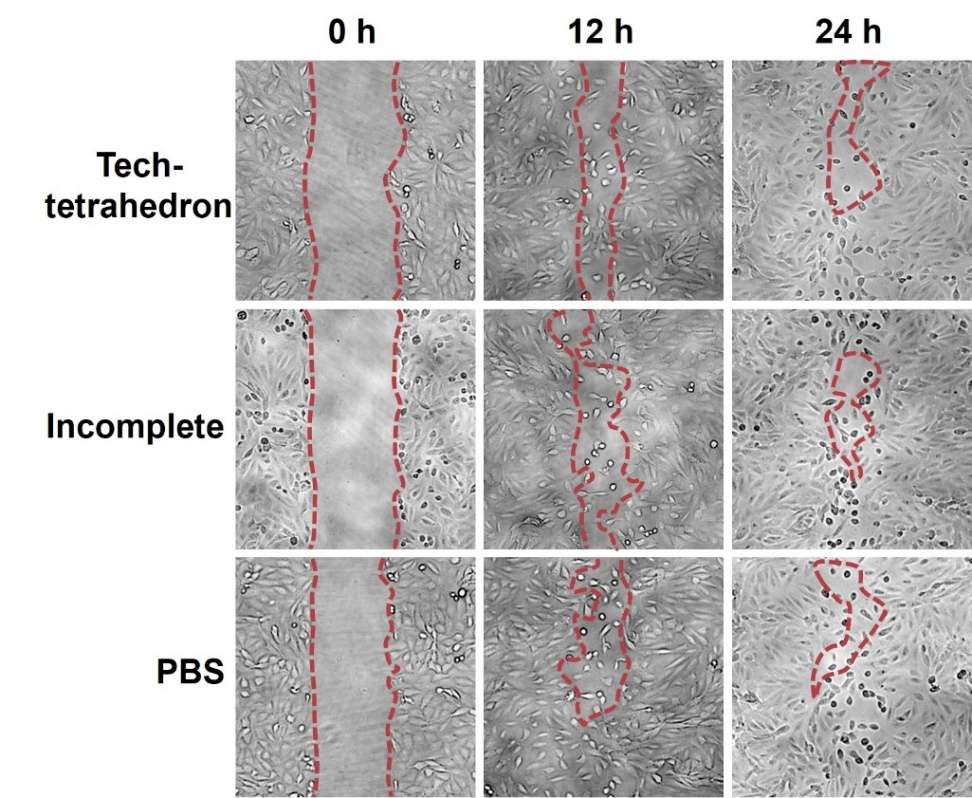


**Figure S33.** Cell scratch healing assay of MCF-10A. MCF-10A cells were treated with Tech-tetrahedron, Incomplete Tech-tetrahedron, PBS, respectively. Cell migration was monitored at 0 h, 12 h, 24 h, respectively. For each gap, twelve measurement points are randomly selected to determine the width, and the mean value is calculated as the average width of the gap at the given time point.


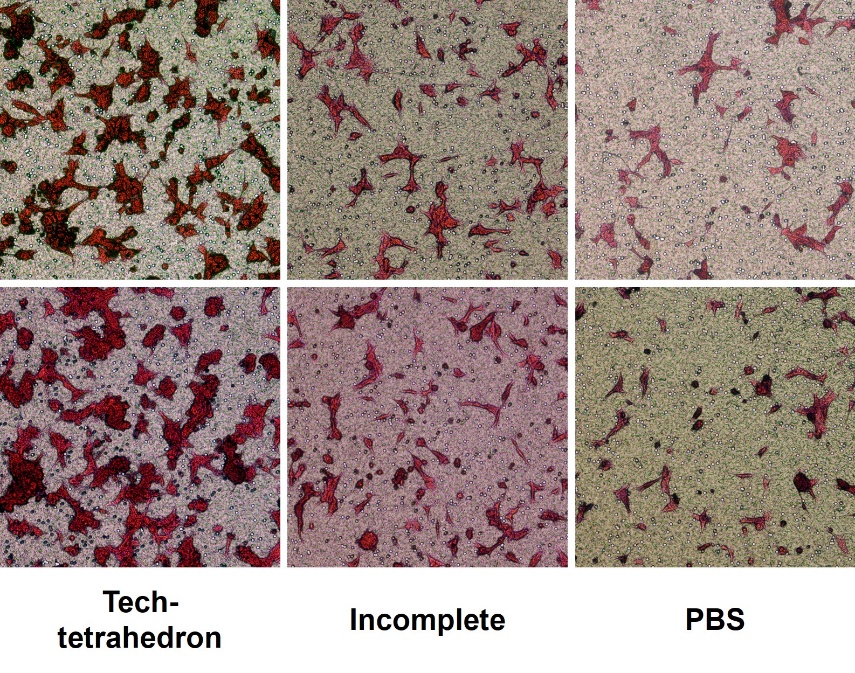


**Figure S34.** Transwell migration assays of MCF-7. MCF-7 cells were treated with Tech-tetrahedron, Incomplete Tech-tetrahedron, PBS, respectively. Calculate the average number of fully intact and successfully stained cells across all fields of view.


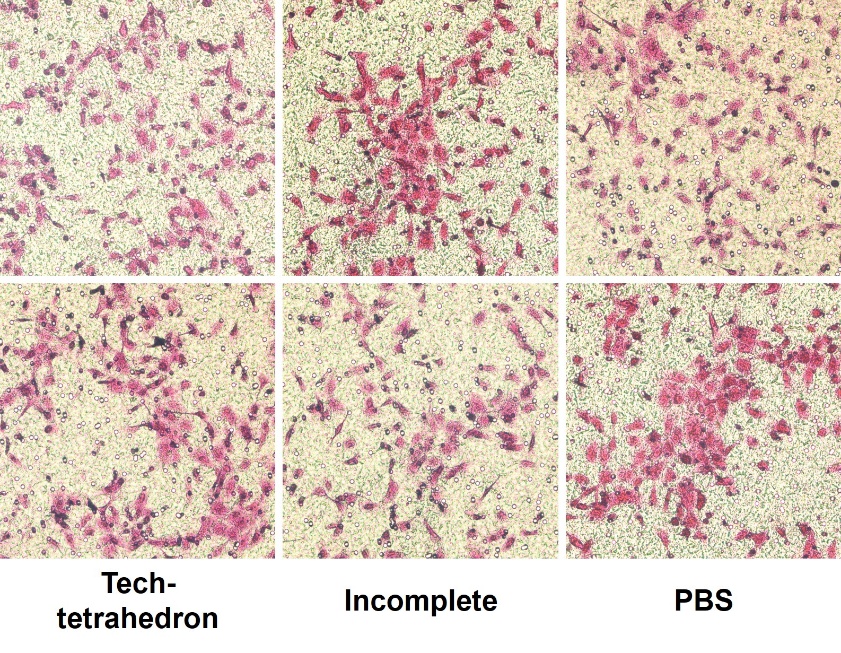


**Figure S35.** Transwell migration assays of MCF-10A. MCF-10A cells were treated with Tech-tetrahedron, Incomplete Tech-tetrahedron, PBS, respectively. Calculate the average number of fully intact and successfully stained cells across all fields of view.


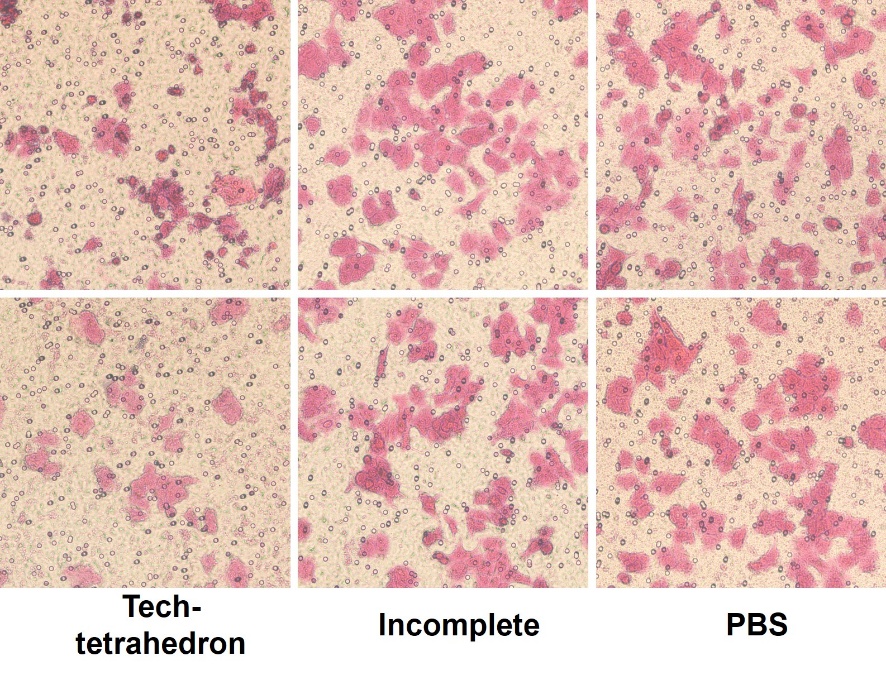


**Figure S36.** Transwell invasion assays of MCF-7. MCF-7 cells were treated with Tech-tetrahedron, Incomplete Tech-tetrahedron, PBS, respectively. Calculate the average number of fully intact and successfully stained cells across all fields of view.


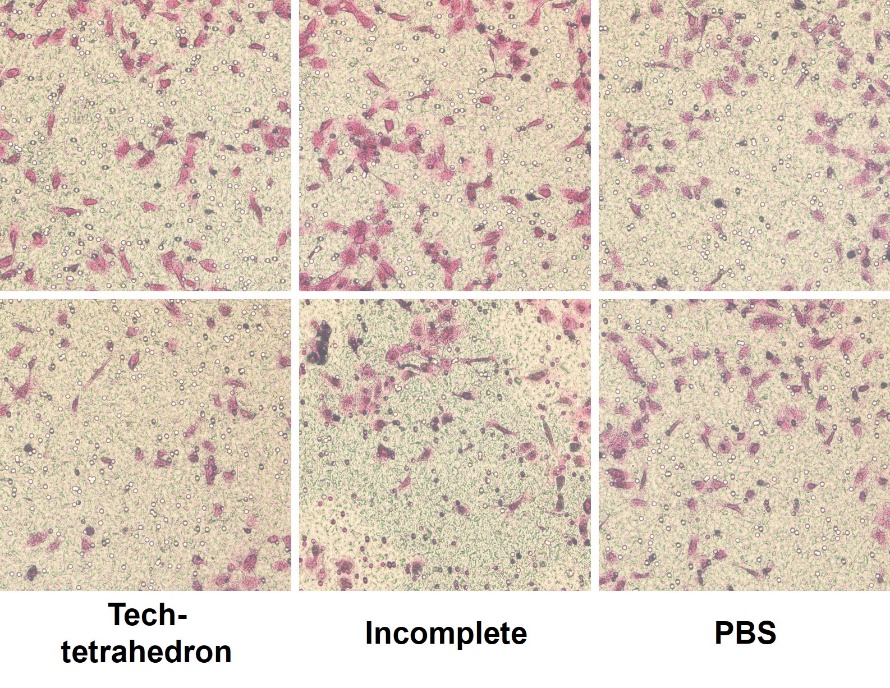


**Figure S37.** Transwell invasion assays of MCF-10A. MCF-10A cells were treated with Tech-tetrahedron, Incomplete Tech-tetrahedron, PBS, respectively. Calculate the average number of fully intact and successfully stained cells across all fields of view.


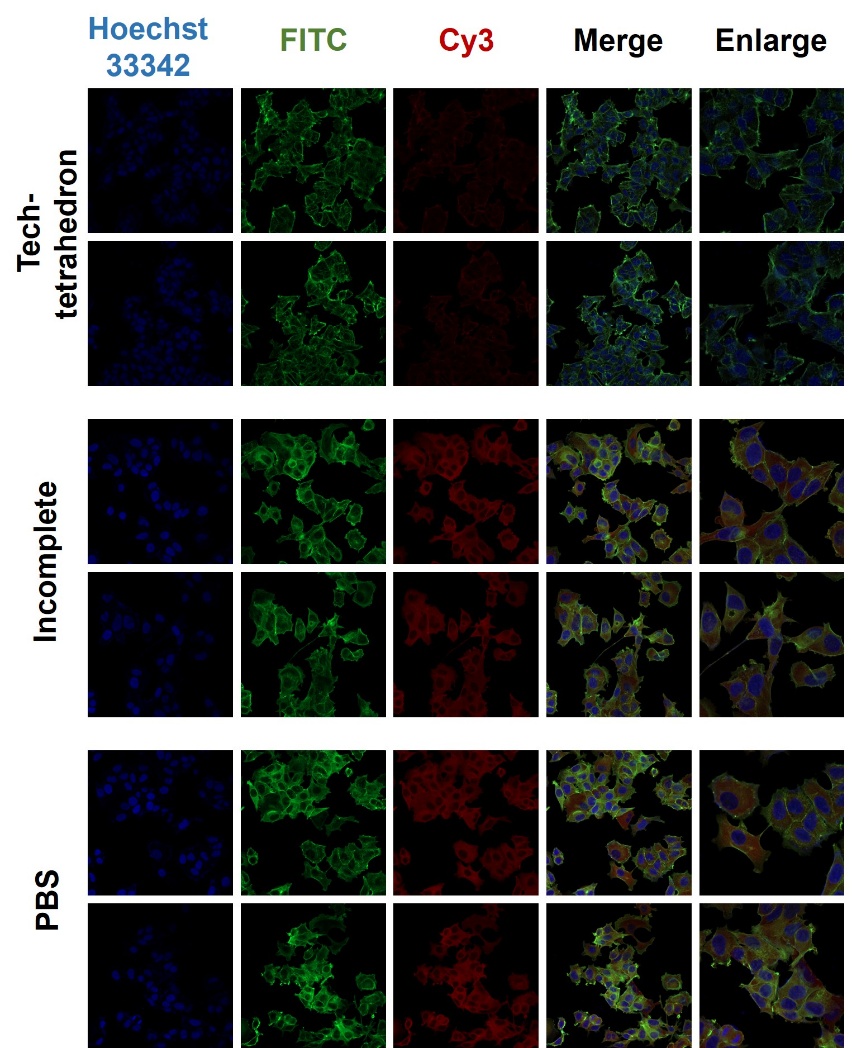


**Figure S38.** Confocal immunofluorescence of F-actin (FITC-phalloidin, green) and cortactin (Cy3, red) with nuclei counterstained using Hoechst 33342 (blue) in MCF-7 cells.


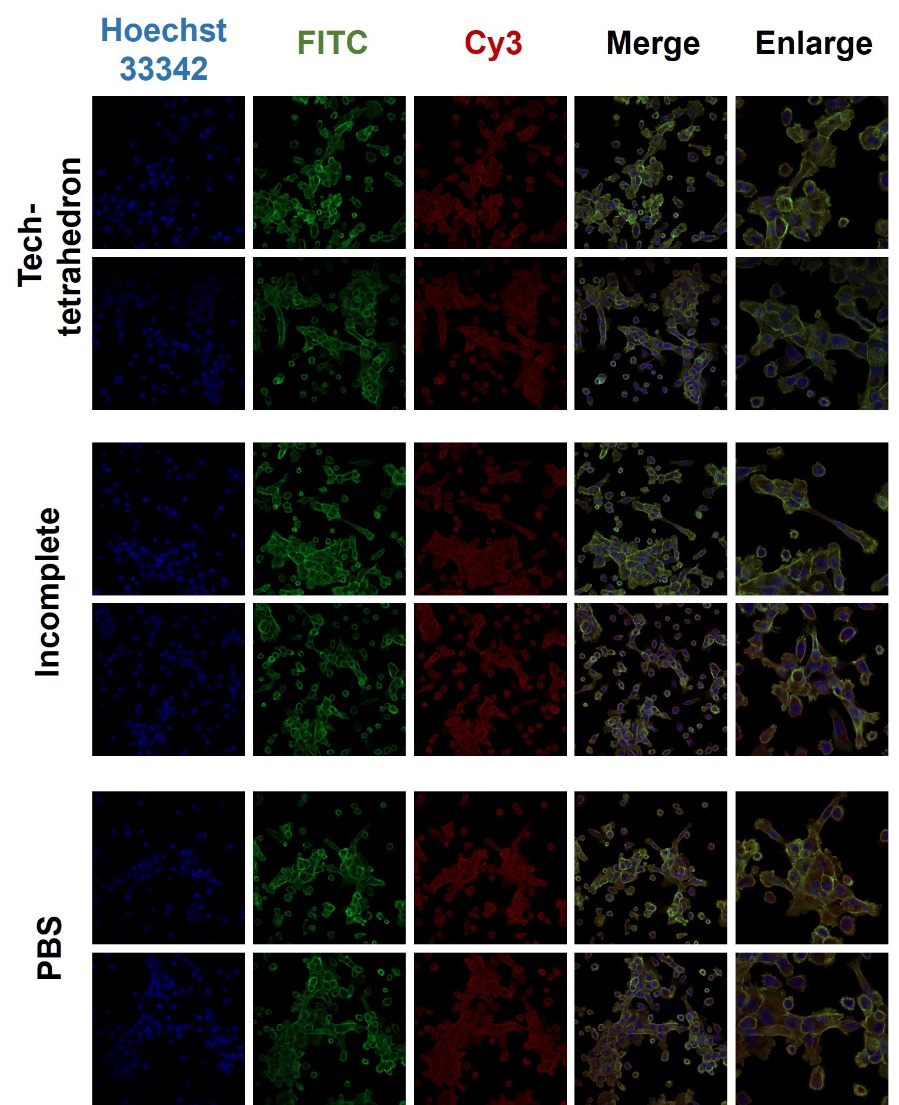


**Figure S39.** Confocal immunofluorescence of F-actin (FITC-phalloidin, green) and cortactin (Cy3, red) with nuclei counterstained using Hoechst 33342 (blue) in MCF-10A cells.


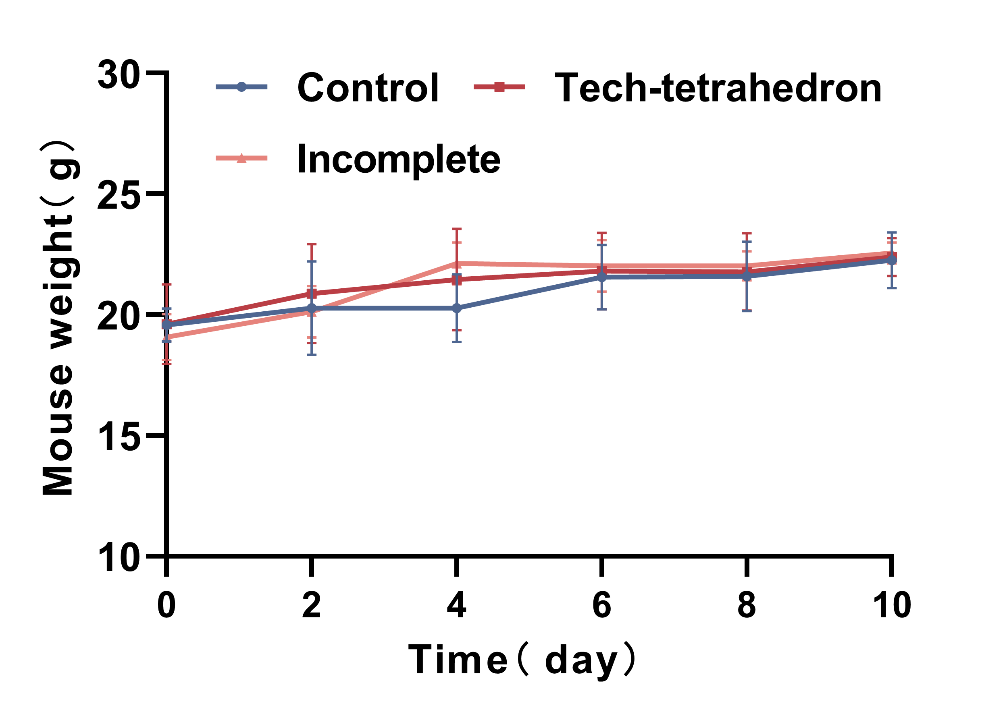


**Figure S40.** Time-dependent body weights alteration of tumor-bearing mice during the treatment periods. Data are presented as mean ± SD (n = 5).


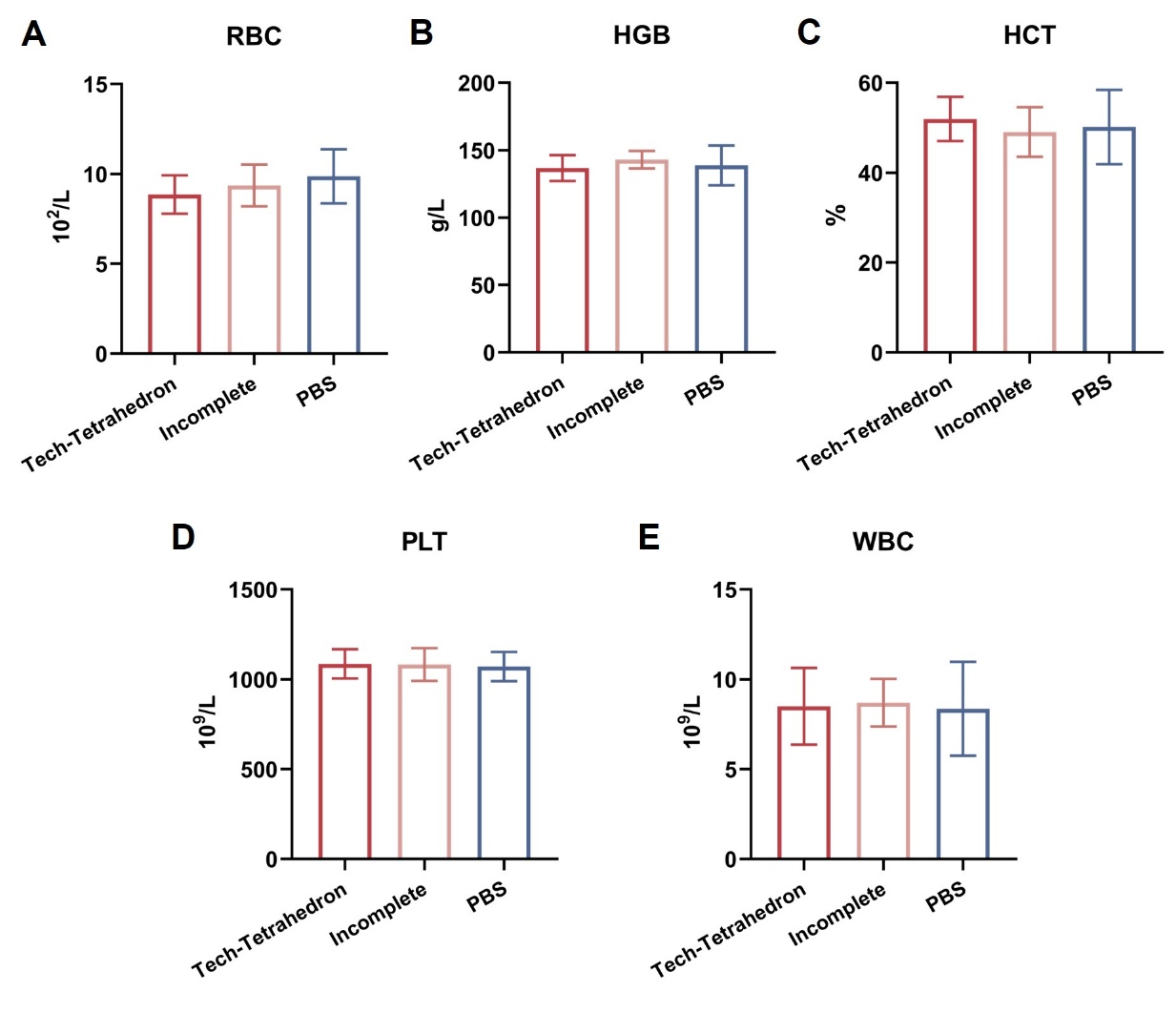


**Figure S41.** Blood routine analysis of mice with different treatments. (A) White blood cell count (RBC) (B) Red blood cell count (HGB) (C) hemoglobin (HCT) (D) hematocrit (PLT) (E) blood platelet count (WBC). Data are presented as mean ± SD (n = 5).


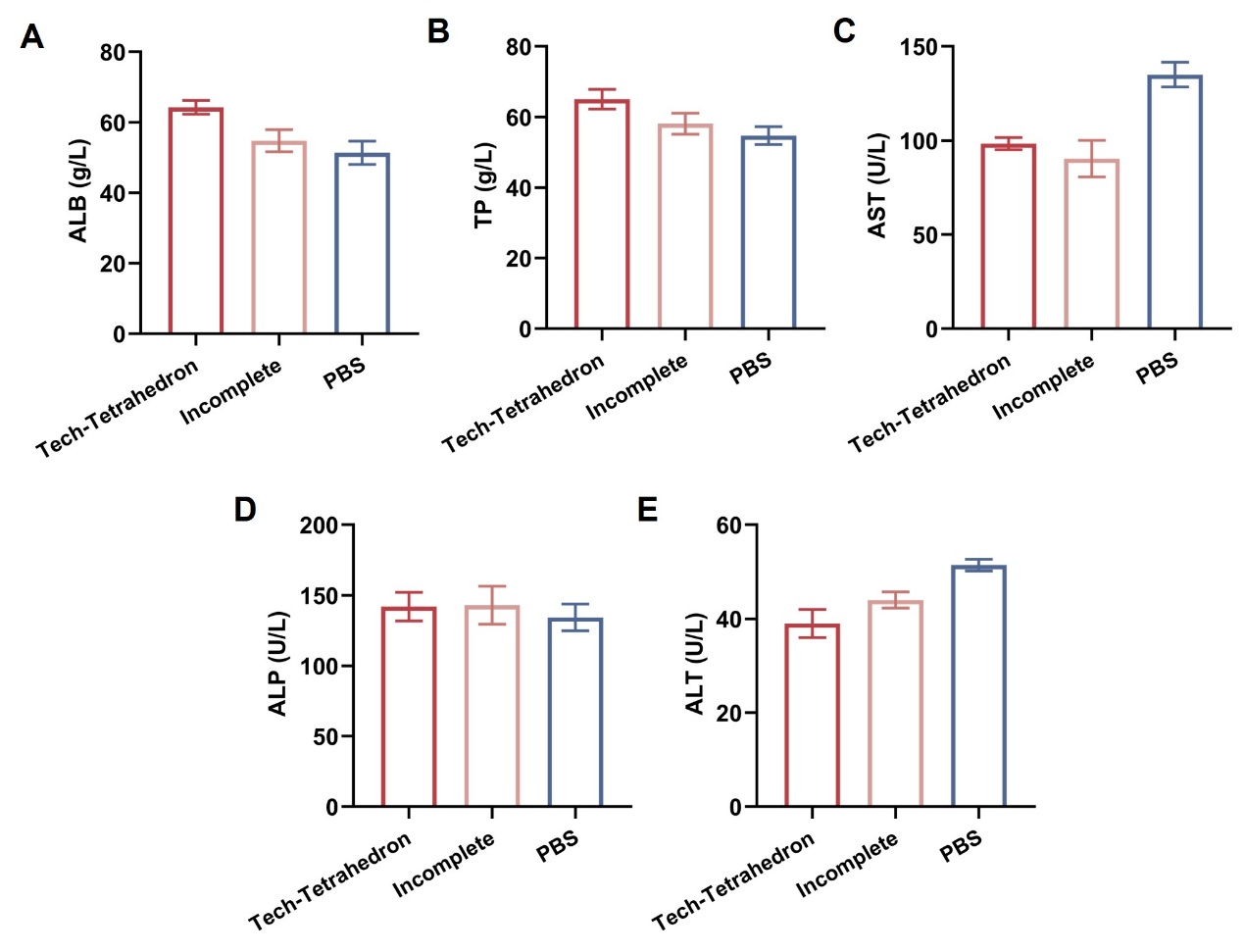
**Figure S42.** Serum Biochemical analysis of mice with different treatments. (A) Albumin (ALB) (B) Total protein (TP) (C) Aspartate aminotransferase (AST) (D) Alkaline phosphatase (ALP) (E) Alanine transferase (ALT). Data are presented as mean ± SD (n = 5).


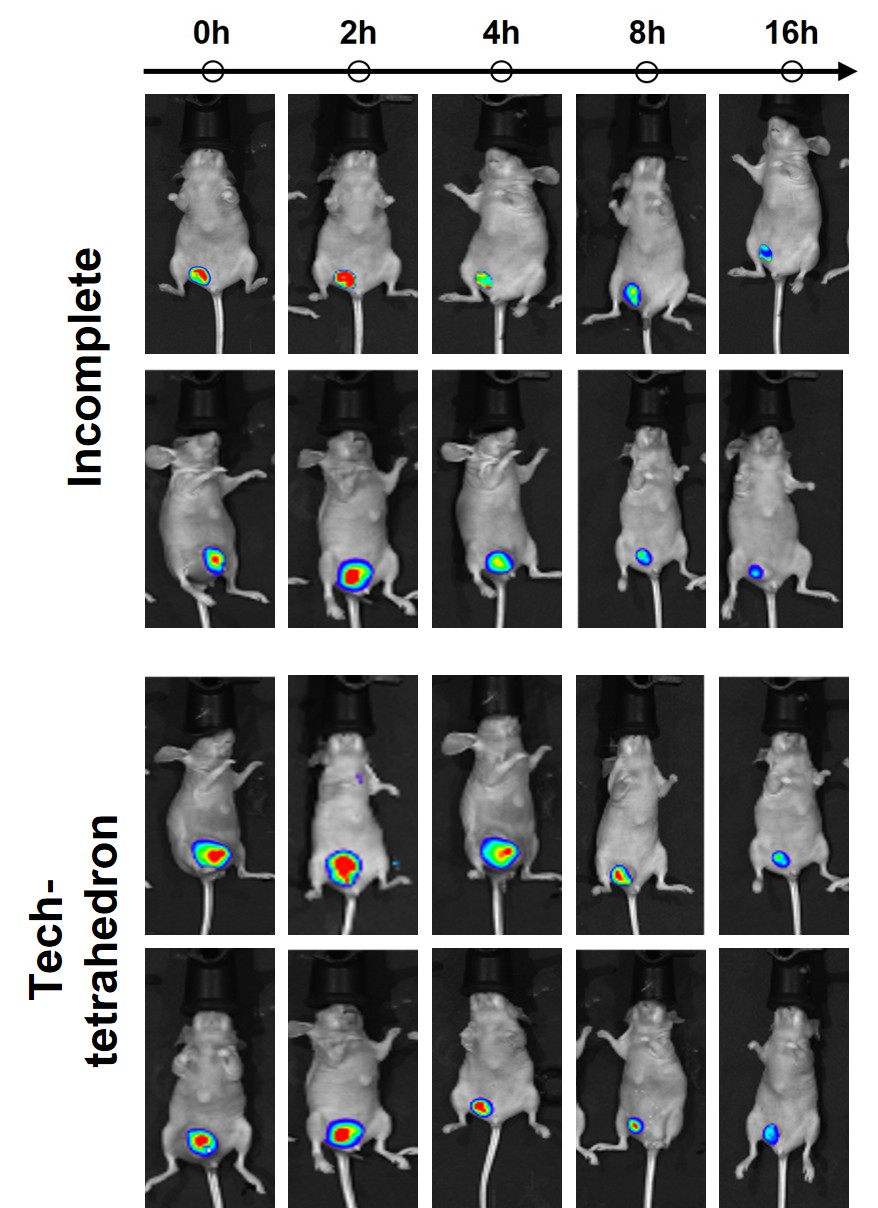


**Figure S43.** Time-dependent epi-ﬂuorescence monitoring results in living mice with diﬀerent treatments.


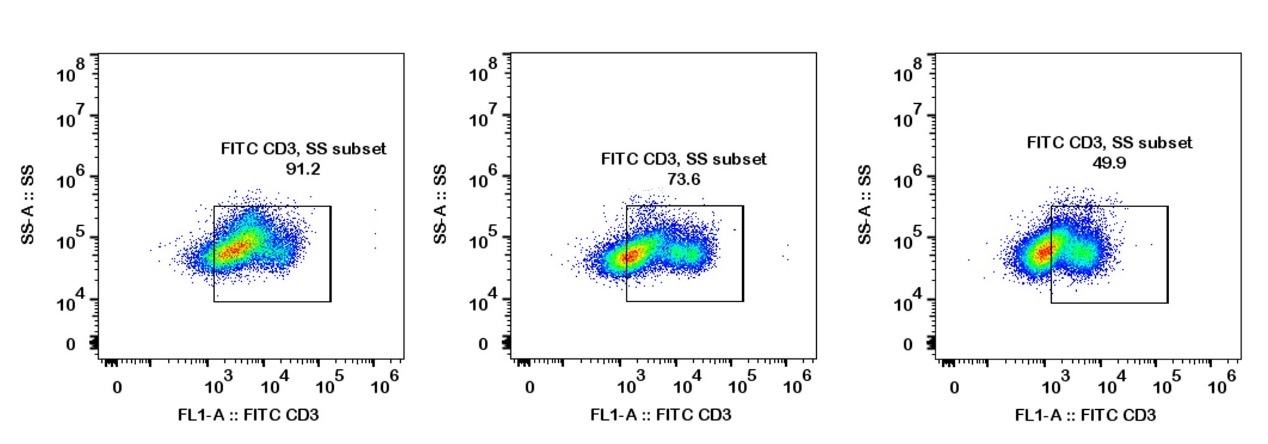
**Figure S44**. Representative gating strategy for splenic T lymphocytes collected on day 18. The rectangular region identifies CD3⁺ events, expressed as a percentage of total lymphocytes (value above each plot).


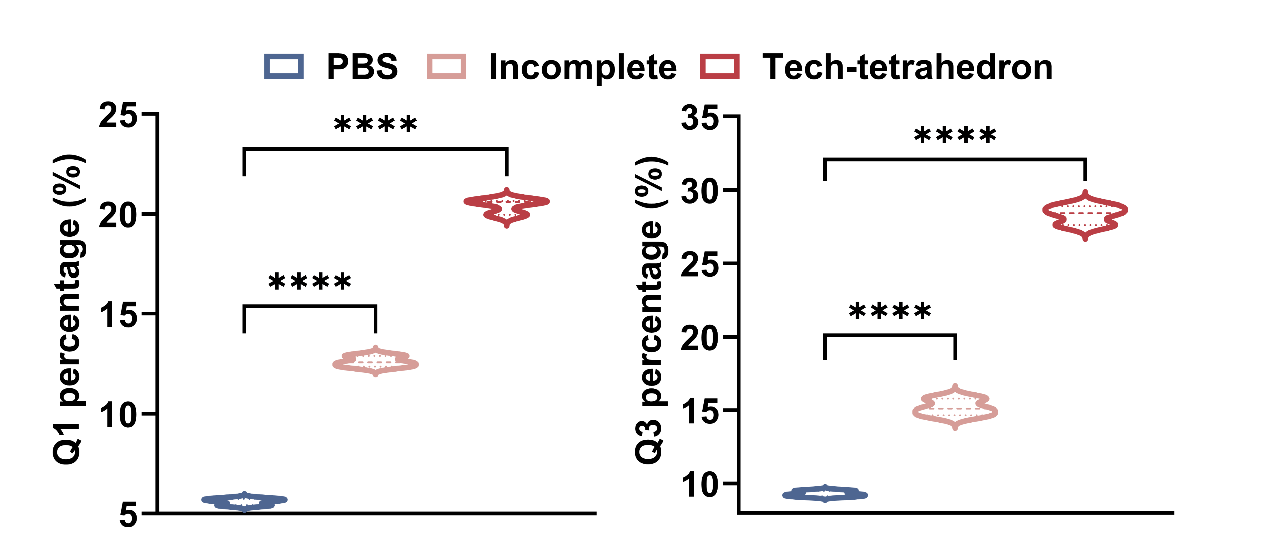


**Figure S45.** Quantification of splenic T cell populations (violin plots, n = 3).

**
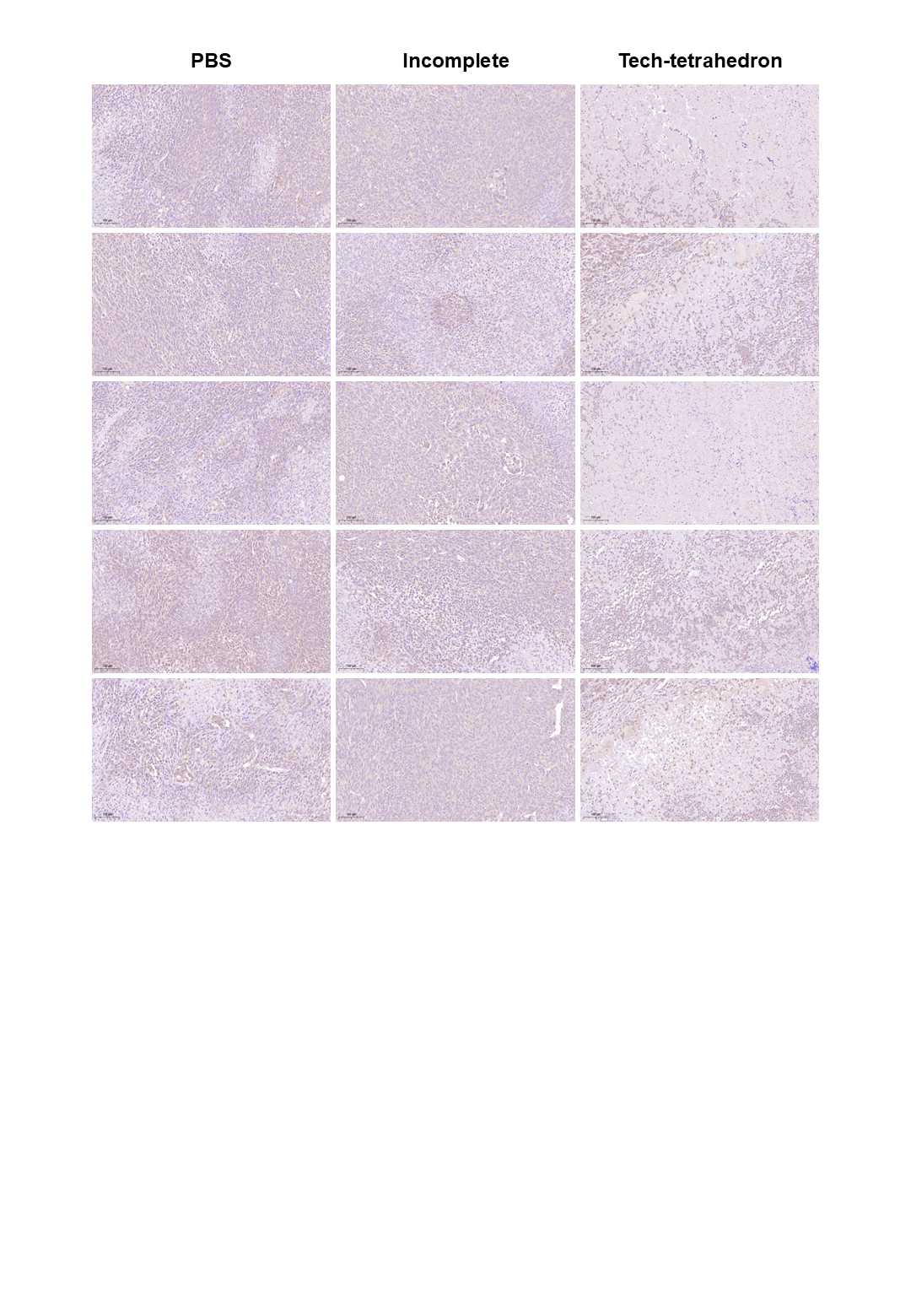
**

**Figure S46.** Representative immunohistochemical micrographs of EGFR membrane staining in day-18 MCF-7 tumors.

**
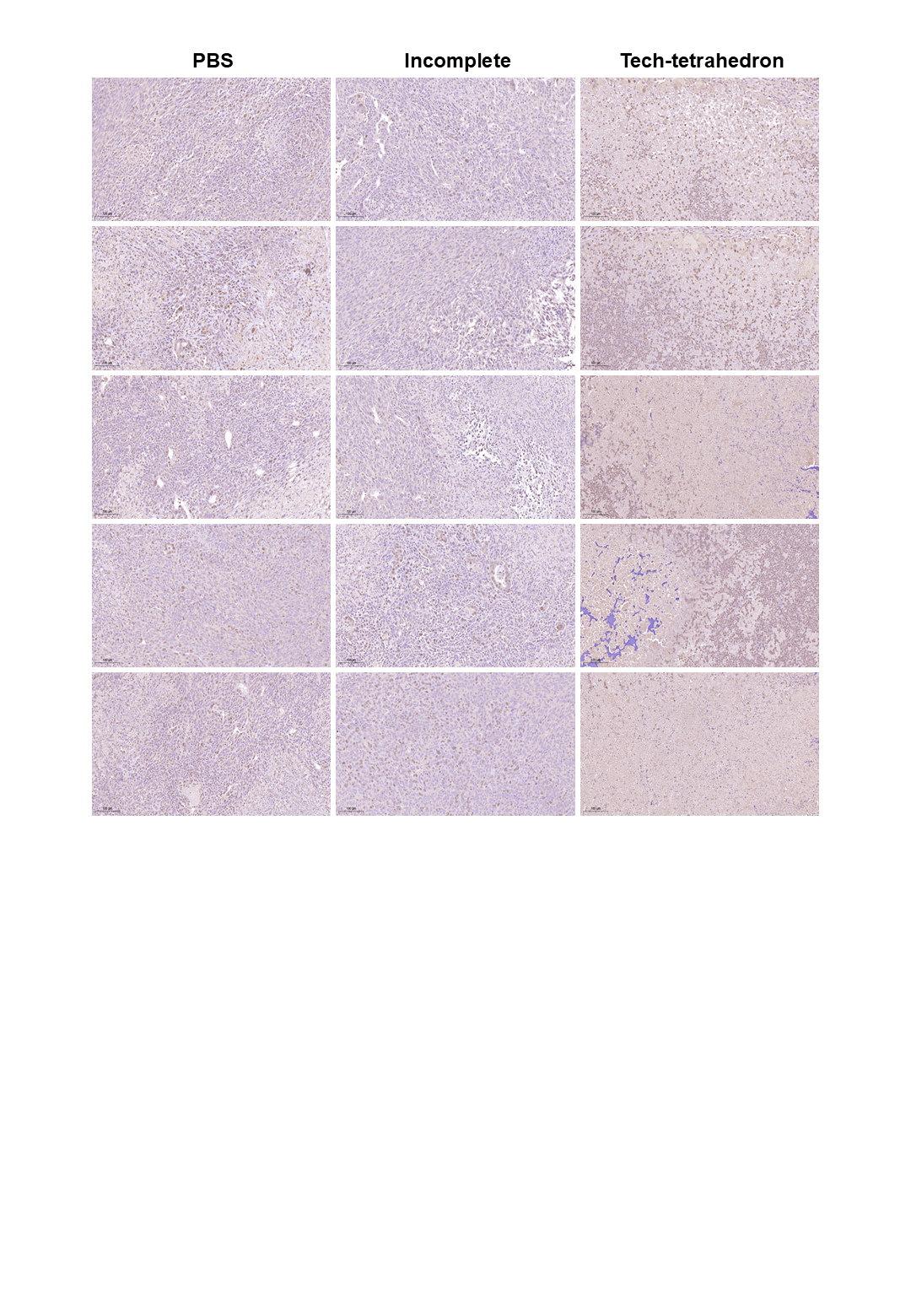
**

**Figure S47.** Representative immunohistochemical micrographs of Ki-67 in day-18 MCF-7 tumors.

**
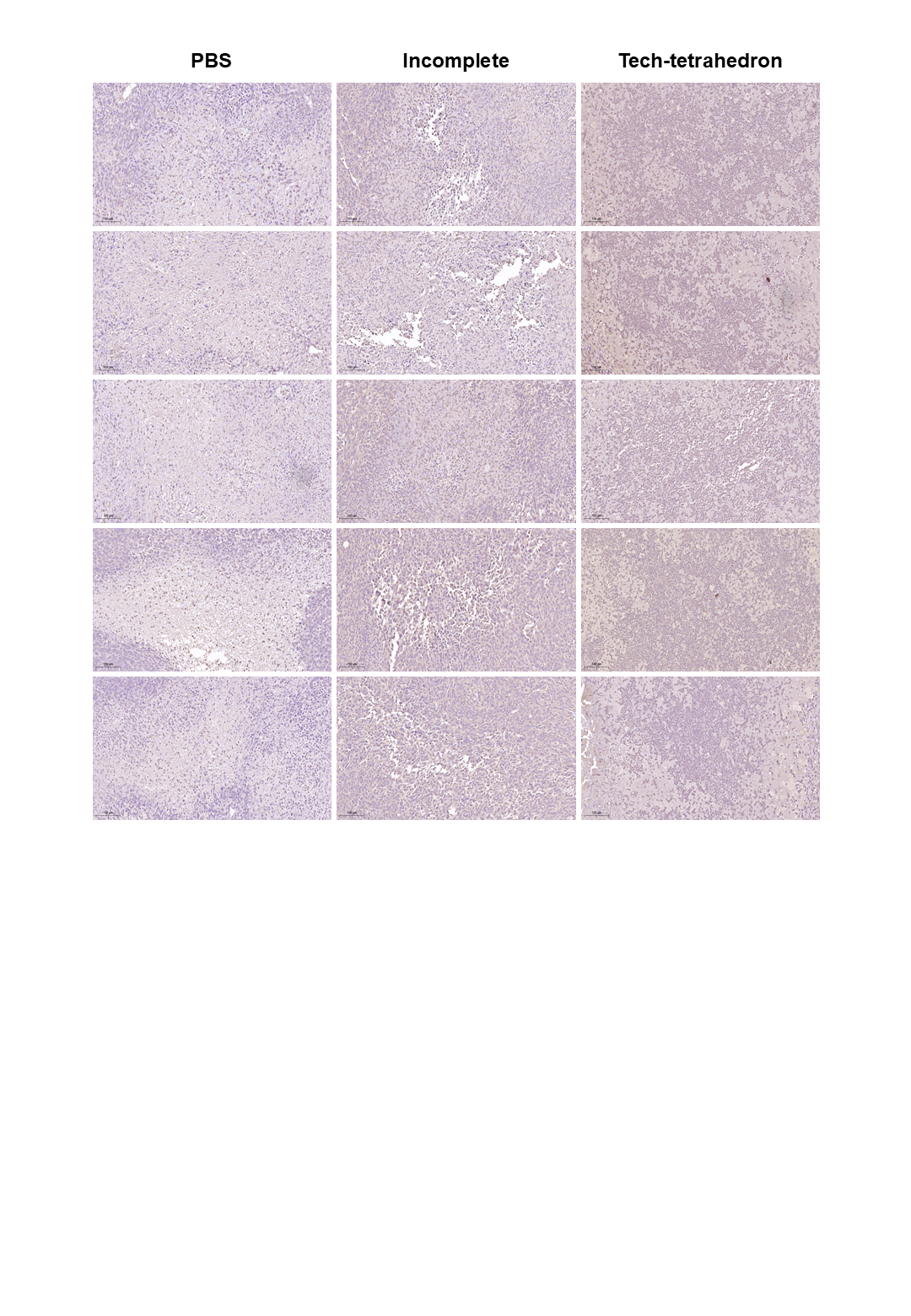
**

**Figure S48.** Representative immunohistochemical micrographs of AR in day-18 MCF-7 tumors.

**
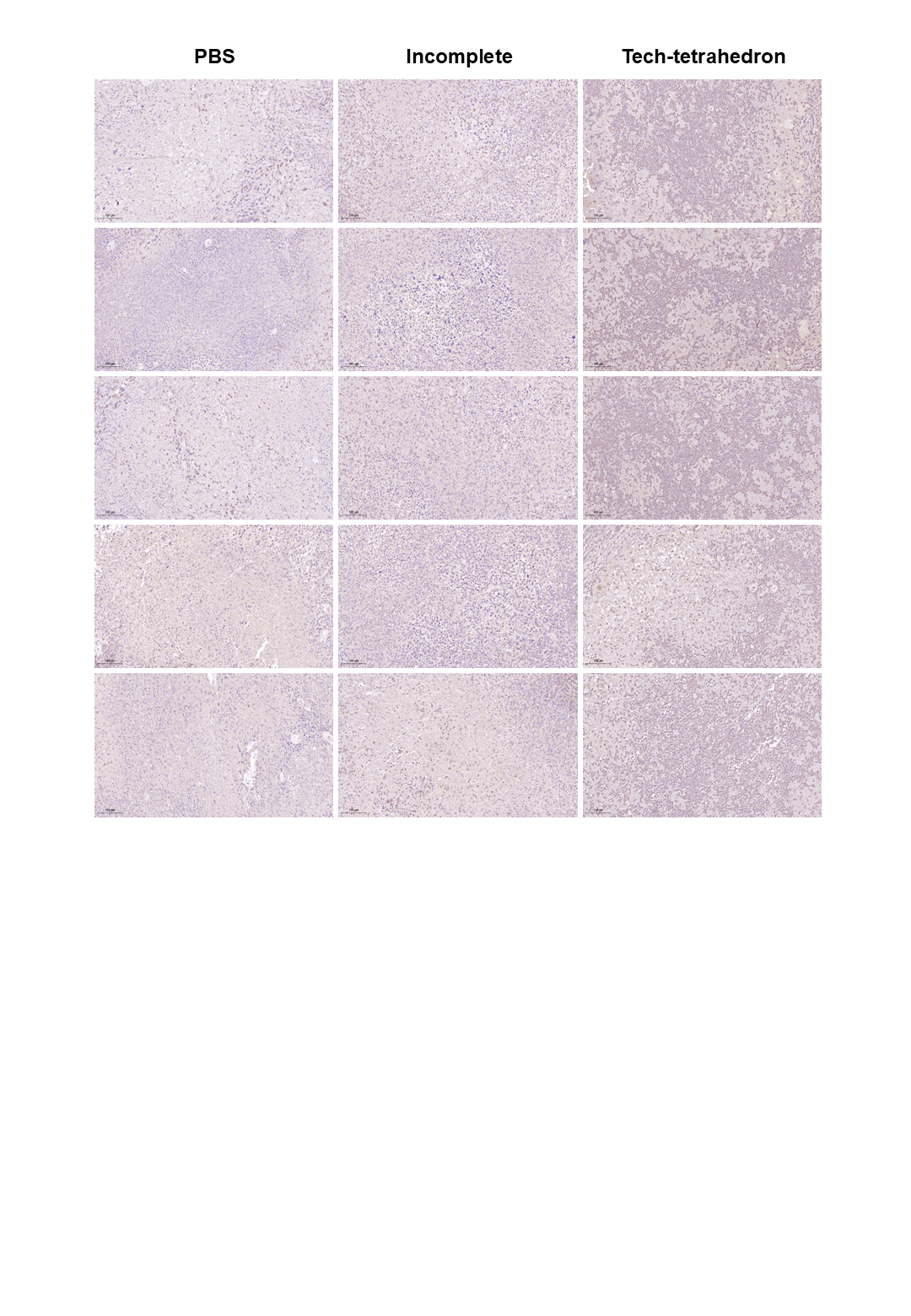
**

**Figure S49.** Representative immunohistochemical micrographs of PC-1 in day-18 MCF-7 tumors.

**
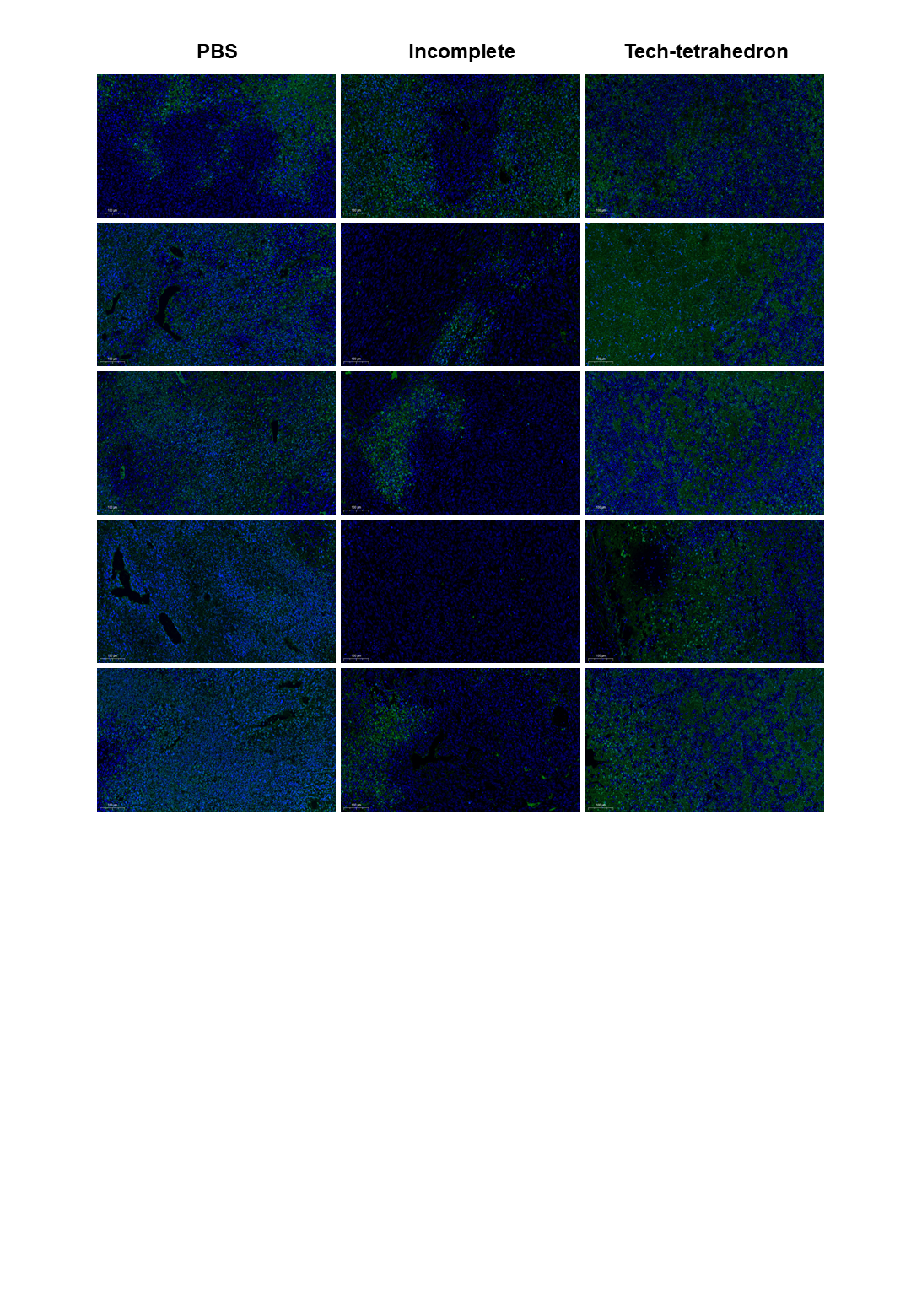
**

**Figure S50.** Representative immunohistochemical micrographs of TUNEL in day-18 MCF-7 tumors.

**Table S1.** Sequence of oligonucleotides used in Tech-tetrahedron.

| Oligonucleotides | Sequence (5'-3') |
| --- | --- |
| P1 | ATGATACCGCCGAGAAGAGCACATCGTTCGACATTACAAAGTCTGAATCCTTACAAAAAAAAAAAAAAAAAAAAAAACC |
| P2 | CATAACCTGGGAGCGTAGATAATGTCGAACGATGTGACAGTTGACGGACCACTATAAAAAAAAAAAAAAAAAAAAAACC |
| P3 | CTTCTCGGCGGTATCATCTAAGGGTGCATCACAGCAAAATAGTGGTCCGTCAACTAAAAAAAAAAAAAAAAAAAAAACC |
| P4 | TACGCTCCCAGGTTATGTTTGCTGTGATGCACCCTTCGTGTAAGGATTCAGACTTAAAAAAAATCCGTCGAGCAGAGTT |
| P5 | TAACCCTAACCCTAACCCTAACTCTGCTCGACGGATT |
| P6 | GGGTTAGGGTTATGTCAATGTC |
| H1 | GGGTTAGGGTTATGTTGAATCGACATTGACATAACCCTAACCCGGTTTTTTTTTTTTTTTTTTTTTT |
| H2 | GATTCAACATAACCCTAACCCCTGTAACGGGTTAGGGTTATGGGTTTTTTTTTTTTTTTTTTTTTT |
| H1b | GGG/iBHQ2dT/TAGGGTTATGTTGAATCGACATTGACATAACCC/i6FAMdT/AACCCGGTTTTTTTTTTTTTTTTTTTTTT |
| H2b | GATTCAACA/iBHQ2dT/AACCCTAACCCCTGTAACGGGTTAGGGTTA/i6FAMdT/GGGTTTTTTTTTTTTTTTTTTTTTT |
| HS1-1 | GGG/iBHQ2dT/TAGGGTTATGTTGAATCGACATTGACATAACCC/i6FAMdT/AACCC |
| HS1-2 | GATTCAACA/iBHQ2dT/AACCCTAACCCCTGTAACGGGTTAGGGTTA/i6FAMdT/G |
| HS2-1 | GGG/iBHQ2dT/TAGGGTTATGTTGAATCGACATTGACATAACCC/i6FAMdT/AACCC |
| HS2-2 | GATTCACA/iBHQ2dT/AACCCTAACCCCTGTAACGGGTTAGGGTTA/i6FAMdT/GTG |
| HS3-1 | GGG/iBHQ2dT/TAGGGTTATGTTGAATCGACATTGACATAACCC/i6FAMdT/AACCC |
| HS3-2 | GAT/iBHQ2dT/CACATAACCCTAACCCCTGTAACGGGTTAGGGTTATG/i6FAMdT/GAA |
| HS4-1 | GGGT/iBHQ2dT/AGGGTTATGTCGAATCGACATTGACATAACCC/i6FAMdT/AACCC |
| HS4-2 | GATTCAACA/iBHQ2dT/AACCCTAACCCCTGTAACGGGTTAGGGTTA/i6FAMdT/G |
| HS5-1 | GGGT/iBHQ2dT/AGGGTTATGTCAAATCGACATTGACATAACCC/i6FAMdT/AACCC |
| HS5-2 | GATTCAACA/iBHQ2dT/AACCCTAACCCCTGTAACGGGTTAGGGTTA/i6FAMdT/G |
| HS6-1 | GGGT/iBHQ2dT/AGGGTTATGTCAATTCGACATTGACATAACCC/i6FAMdT/AACCC |
| HS6-2 | GATTCAACA/iBHQ2dT/AACCCTAACCCCTGTAACGGGTTAGGGTTA/i6FAMdT/G |
| PKD1-F | CTTCCGGTGGACCATCAACG |
| PKD1-R | GCCGCGCTCTGATAAATGAC |

^a)^ The primer sequence of the tetrahedron (TE) in P4 is highlighted in green

^b)^ The sequences in H1 that are complementary to P6 are highlighted in red.

^c)^ Underlined characters represent the stem regions in H1 and H2.

**Table S2.** Sequence of oligonucleotides used in linear double-hairpin DNA construct.

| Oligonucleotides | Sequence (5'-3') |
| --- | --- |
| H1 | GGGT/iBHQ2dT/AGGGTTATGTTGAATCGACATTGACATAACCC/i6FAMdT/AACCCGGTTTTT |
| H2 | GATTCAACATAACCCTAACCCCTGTAACGGGTTAGGGTTATGGGTTTTTTT - TPP |
| Activator | GGGTTAGGGTTATGTCAATGTC |

^a)^ The underlined sequences are the complementary sequences.

^b)^ The sequence in red is the binding site of H1 and Activator.

**Table S3:** Sequence of oligonucleotides used in mut-CHA.

| Oligonucleotides | Sequence (5'-3') |
| --- | --- |
| H1-mut | GGGT/iBHQ2dT/AGGGTTATGTTGAATCGACATTGACATAACCC/i6FAMdT/AACCCGGTTTTTTTTTTTTTTTTTTTTTT |
| H2-control | GATTCAACATAACCCTAACCCCTGTAACGGGTTAGGGTTATGGGTTTTTTTTTTTTTTTTTTTTTT |
| H2-mut | CTAAGTTCAATTCGCATTCGCCTGTAACGGGTTAGGGTTATGGGTTTTTTTTTTTTTTTTTTTTTT |

^a)^ The underlined sequences are the complement.

^b)^ The mutated bases are marked in red.

**Table S4.** Differentially expressed genes identified as primary factors.

**
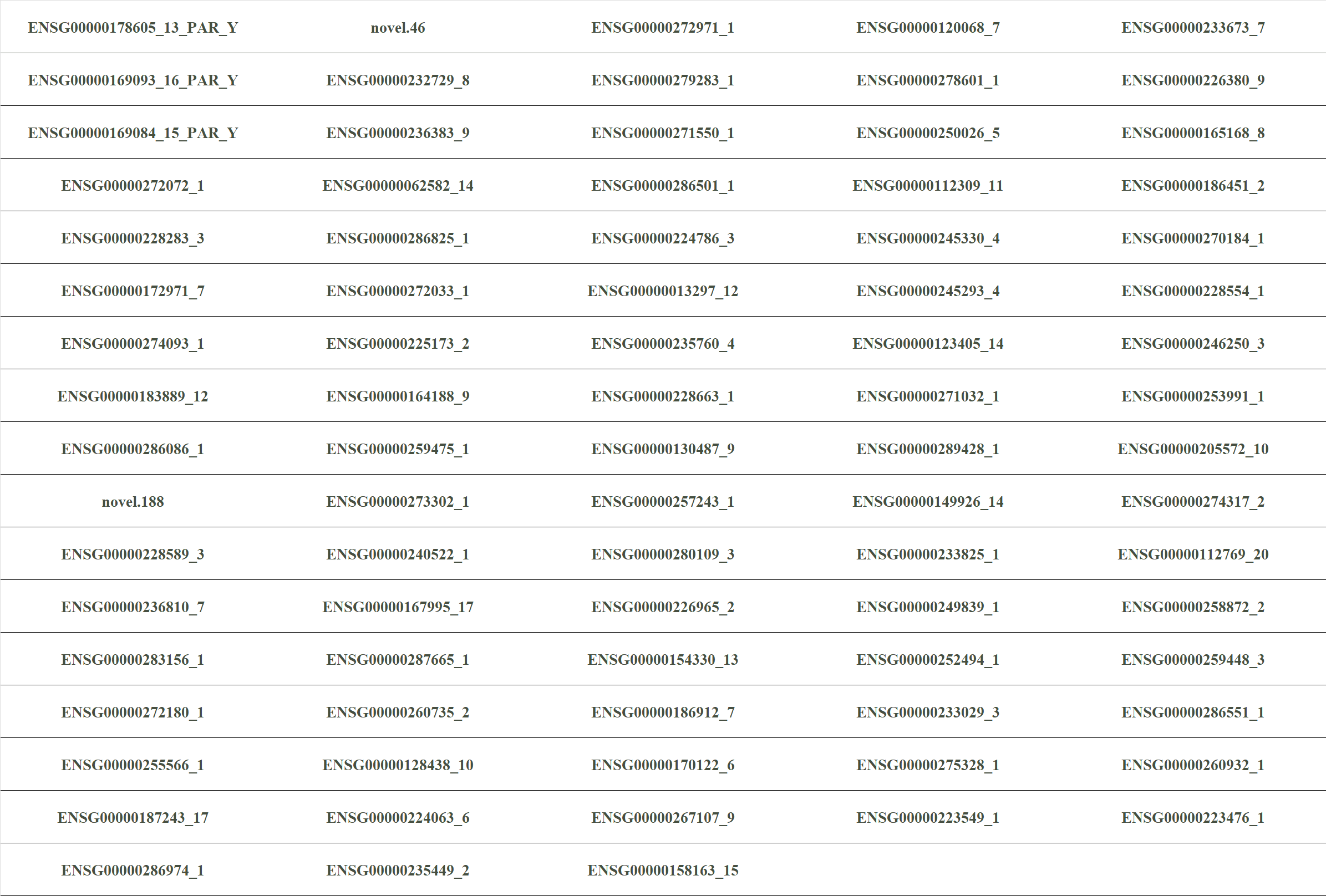
**

**Table S5.** Differentially expressed genes identified as confounding factors. **
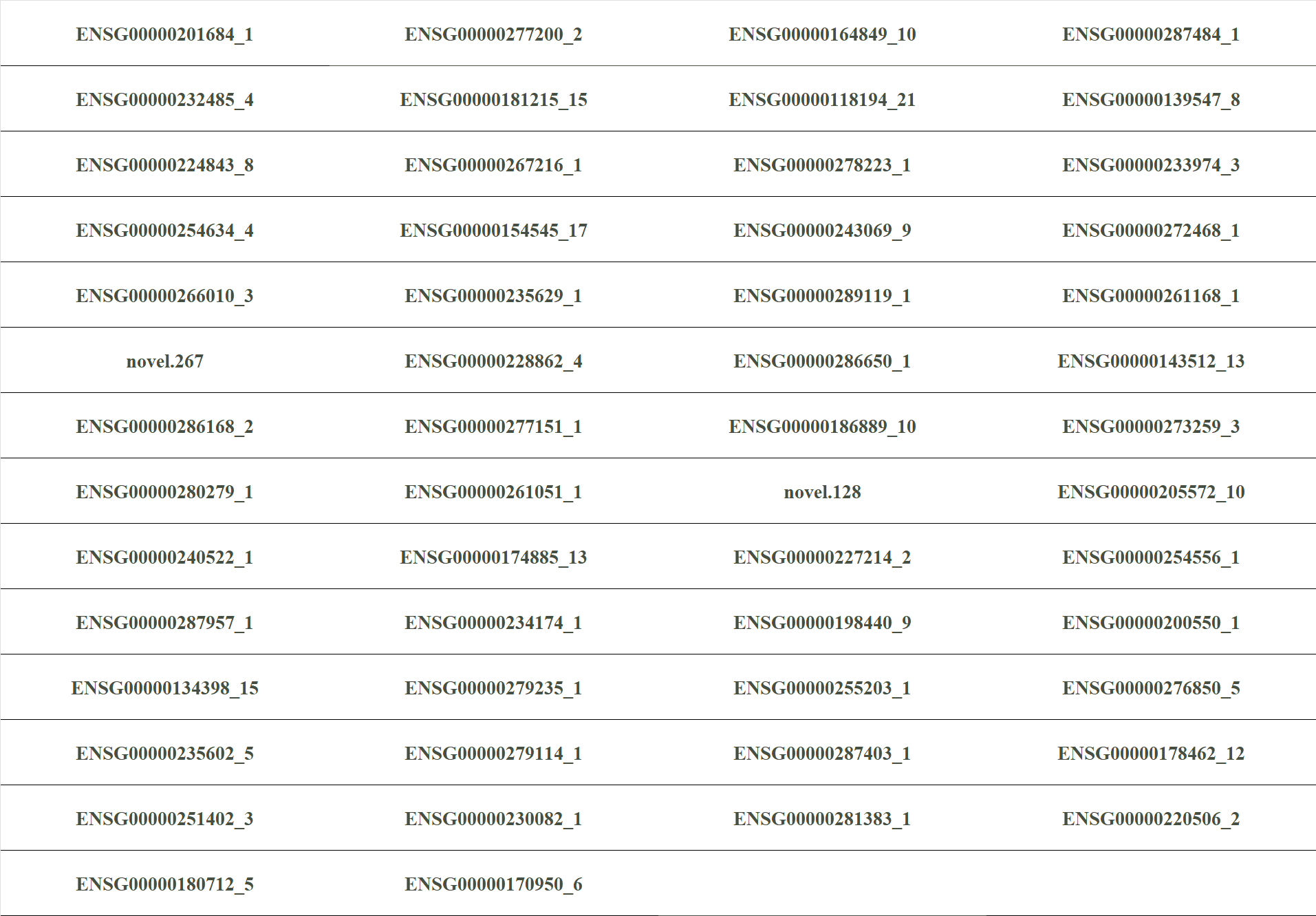
**

**Table S6.** Genes specifically responsive to the formation of complete aggregates. **
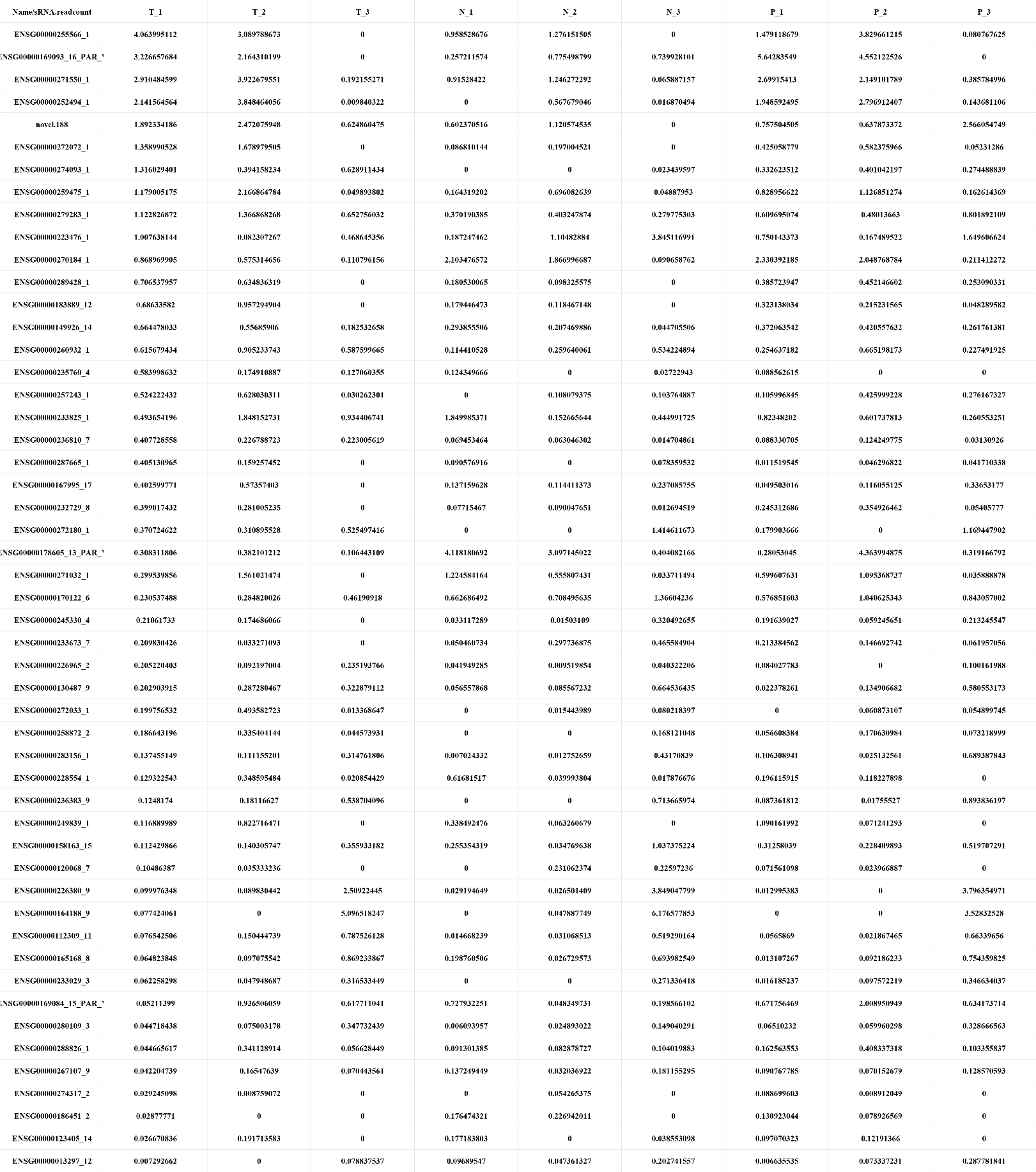
**
